# Potential Therapeutic Targets in Triple-Negative Breast Cancer Based on Gene Regulatory Network Analysis: A Comprehensive Systems Biology Approach

**DOI:** 10.1155/2024/8796102

**Published:** 2024-10-22

**Authors:** Maryam Ahmadi, Neda Barkhoda, Aida Alizamir, Amir Taherkhani

**Affiliations:** ^1^Clinical Research Development Unit of Fatemiyeh Hospital, Department of Gynecology, School of Medicine, Hamadan University of Medical Sciences, Hamadan, Iran; ^2^Department of Pathology, School of Medicine, Hamadan University of Medical Sciences, Hamadan, Iran; ^3^Research Center for Molecular Medicine, Hamadan University of Medical Sciences, Hamadan, Iran

**Keywords:** biomarkers, breast cancer, pathogenesis, prognosis, treatment

## Abstract

**Background:** Triple-negative breast cancer (TNBC) is an aggressive subtype with limited treatment options. This study is aimed at identifying potential therapeutic targets in TNBC using gene regulatory network analysis and a system biology approach.

**Methods**: The GSE38959 dataset was reanalyzed to identify differentially expressed genes (DEGs) in TNBC tissues compared to normal breast samples. Protein–protein interaction networks were constructed, and hub genes were identified. Survival analysis was performed using GEPIA2. Gene regulatory networks were built to identify upstream regulators. Cross-platform verification was conducted using an independent RNA-seq dataset (GSE58135). Expression analysis of prognostic markers in TNBC versus non-TNBC samples was performed using bc-GenExMiner.

**Results:** A total of 943 DEGs were identified in TNBC tissues. CHEK1 and PLK1 were identified as central hub genes, with overexpression correlating with poor prognosis. GABPB1 was identified as the most influential upstream regulator of CHEK1 and PLK1 through gene regulatory network analysis, while JUN exhibited the most interactions among regulators. A total of 302 consistently modulated genes were confirmed through cross-platform verification. The overexpression of CHEK1 and PLK1 in TNBC compared to non-TNBC samples was validated by expression analysis.

**Conclusion**: This study provides insights into the molecular mechanisms of TNBC and suggests CHEK1, PLK1, and their upstream regulators as potential therapeutic targets for TNBC treatment.

## 1. Introduction

Following the most recent Global Cancer Statistics (GLOBOCAN) 2020 estimates, breast cancer among females has exceeded lung cancer as the predominant cancer type in terms of global incidence. Approximately 2.3 million new cases were diagnosed in 2020 [1]. Triple-negative breast cancer (TNBC) is a highly aggressive subtype [2] characterized by the absence of estrogen receptor (ER), progesterone receptor (PR), and human epidermal growth factor receptor 2 (HER2) expression. The prevalence of TNBC varies across populations, as indicated by a systematic review and meta-analysis in Africa, estimating a pooled TNBC frequency of 27.0% (95% CI: 24.0%–30.2%). Notably, West Africa reported the highest prevalence at 45.7% (95% CI: 38.8%–52.8%), while Central Africa had the lowest at 14.9% (95% CI: 8.9%–24.1%) [3]. In Shaanxi Province, China, from 2010 to 2015, the prevalence of TNBC was reported as 29.57% among a cohort of 328 breast cancer patients [4]. A study in India registered a TNBC prevalence of 27%, highlighting a higher likelihood of TNBC in younger age groups, presenting with high grades and exhibiting lymph node positivity compared to non-TNBC [5]. Regarding mortality, a West African study emphasized a higher mortality rate for TNBC compared to other breast cancer subtypes [6]. In China, the 5-year survival rate for TNBC patients was recorded at 66.4% [4], underscoring the importance of these outcomes in comprehending and addressing TNBC. Examining genetic factors, a Japanese study identified deleterious mutations in the BRCA1 or BRCA2 genes in 20.0% of TNBC patients [7].

Due to the elevated prevalence and unfavorable prognosis associated with TNBC [8], it is imperative to promptly explore novel therapeutic strategies aimed at addressing this formidable medical condition [9, 10]. The promise of systems biology in treating TNBC is evident in its ability to identify crucial genes, pathways, and biological processes relevant to TNBC and its treatment. This study contributes to developing innovative therapeutic strategies, shedding light on potential novel targets, and enabling personalized therapy for TNBC and its resistance mechanisms [11, 12]. To further explore this potential, a comprehensive bioinformatics study was conducted to identify prognostic genes associated with poor outcomes in TNBC patients. Subsequent construction of a gene regulatory network (GRN) showcased the upstream regulators governing these prognostic markers. This investigation successfully achieved its objectives by reanalyzing the gene expression profile dataset GSE38959, initially curated by Komatsu et al. [13]. The focus of this reassessment was on discerning differentially expressed genes (DEGs) within TNBC tissues in comparison to the gene expression profile of normal breast specimens.

This study offers valuable insights into the molecular basis of TNBC. A comprehensive bioinformatic analysis of the GSE38959 dataset revealed 943 genes with differential expression in TNBC tissues compared to healthy breast samples. Notably, protein–protein interaction (PPI) network analysis identified CHEK1 and PLK1 as central hub genes with overexpression correlating with poor prognosis in invasive breast carcinoma. Functional analyses highlighted key pathways potentially involved in TNBC progression, including the activation of PKN1 stimulating the transcription of androgen receptor (AR)–regulated genes KLK2 and KLK3 and sirtuin-1's (SIRT1) negative regulation of rRNA expression. Furthermore, GRN analysis revealed GA–binding protein subunit beta-1 (GABPB1) as the most influential upstream regulator of CHEK1 and PLK1, while JUN exhibited the most interactions among the identified regulators. These findings suggest potential prognostic markers and illuminate the molecular mechanisms driving malignant transformation in TNBC. Consequently, therapeutic interventions targeting CHEK1, PLK1, and their upstream regulators, such as GABPB1 and JUN, could represent promising strategies for TNBC treatment.

## 2. Methods

### 2.1. Sample Collection

Komatsu et al. [13] procured 48 TNBC specimens, excluding 18 cases from DNA microarray analysis. Additionally, 13 normal mammary tissues were obtained with informed consent from patients treated at Tokushima Breast Care Clinic in Tokushima, Japan. The study protocol received approval from the Ethics Committee of The University of Tokushima. Clinical information was obtained from medical records, and pathologists confirmed the diagnosis of triple-negative tumors through immunohistochemical staining, indicating negativity for ER, PR, and HER2 with a score of 0 or 1+. Tissue samples were promptly embedded in the TissueTek OCT compound (Sakura, Tokyo, Japan), frozen, and stored at −80°C for subsequent analysis. The features of scanned image files containing Cy3-fluorescence signals from hybridized Agilent Microarrays were extracted using Agilent Feature Extraction software (Version 9.5) provided by Agilent Technologies.

### 2.2. Microarray Dataset Recovery and Statistical Analysis

A reanalysis of the gene expression dataset GSE38959 [13] was conducted, accessible through the following link: http://www.ncbi.nlm.nih.gov/geo [14]. The original dataset was generated using the GPL4133 platform (Agilent-014850 Whole Human Genome Microarray 4x44K G4112F), serving as the foundational platform for this study. The DEGs in TNBC tissues (*n* = 30) were discerned in comparison to normal breast samples (*n* = 13) using the orthogonal partial least squares (OPLS) method within the R programming environment Version 4.3.2. The identification process involved criteria of an adjusted *p* value < 0.01 and an absolute Log2 fold change (FC) exceeding 1.585. Additionally, the volcano plot of the dataset GSE38959 was presented through the Shiny server, accessible via https://huygens.science.uva.nl/ [15].

### 2.3. PPI and Functional Analyses

The interactions between DEGs were explored using the STRING Version 12.0 knowledge base, accessible at http://string-db.org [16]. Single proteins were systematically removed from the network using the setting tool to enhance the precision of our analysis [17]. Subsequently, the network file was imported into Cytoscape 3.10.1, which can be found at https://cytoscape.org/ [18]. This allowed us to visualize the protein–protein interaction network (PPIN) and compute the centrality of nodes within this protein graph. Hub genes were identified based on criteria such as degree centrality exceeding twice the average, and closeness and betweenness centralities surpassing the network's average.

The identified hub genes were subjected to further scrutiny to assess their potential role in influencing the prognosis of patients affected by TNBC. This analysis used the GEPIA2 web server, accessible at http://gepia2.cancer-pku.cn/#index [19].

Clusters within the PPIN were identified using the MCODE plugin to unravel the biological processes and pathways involved in the malignant transition from normal breast tissue to a TNBC state [20]. Prominent modules meeting specific criteria, such as an MCODE score surpassing three and a gene count exceeding 10, were considered noteworthy and selected for subsequent pathway and biological process analysis [21].

The examination of pathways and biological processes influenced by the identified clusters was done by utilizing the g:Profiler web server, accessible at https://biit.cs.ut.ee/gprofiler/gost [22]. Additionally, the discernment of molecular functions and cellular components affected in TNBC was accomplished by importing DEGs into the g:Profiler tool. Significance was established by adhering to a defined cut-off criterion, requiring a false discovery rate (FDR) < 0.05 and a minimum of 10 enriched genes within each specific term.

### 2.4. Kaplan–Meier and Boxplot Analyses

The evaluation of prognostic implications associated with hub genes in TNBC involved the generation of Kaplan–Meier's curves, conducted through the analytical capabilities of the GEPIA2 database, accessible at http://gepia2.cancer-pku.cn/#survival [23]. Prognostic relevance was determined via the Log-rank test, where genes with a hazard ratio (HR) *p* value below 0.05 were deemed to have a significant prognostic impact.

GEPIA2 employs advanced analytical techniques to scrutinize RNA sequencing data derived from The Cancer Genome Atlas [24] and the Genotype-Tissue Expression [25] databases. This meticulous approach ensures reliable and robust results, particularly in survival and box plot analyses, when comparing cancer patients to their healthy counterparts. Additionally, the expression patterns of prognostic hub genes within TNBC tissues and normal samples underwent a comprehensive assessment utilizing pertinent data sourced from the GEPIA2 database.

### 2.5. GRN

The primary objective of the current study was to demonstrate upstream regulators orchestrating the transcription of prognostic markers linked to adverse outcomes in TNBC patients [26]. To achieve this, the iRegulon plugin in the Cytoscape software was utilized to identify potential transcription factors governing the regulation of these prognostic genes. The iRegulon computes a normalized enrichment score (NES) for each transcription factor, and significance was attributed to those regulators with an NES value exceeding three, as stipulated by the established threshold [27].

### 2.6. Cross-Platform Verification

To reinforce the reliability of the initial microarray findings and validate the identified DEGs, a cross-platform validation strategy was implemented utilizing an independent RNA-sequencing (RNA-seq) dataset (GSE58135) procured from the GEO database. This dataset encompassed RNA-seq data from TNBC primary tumors (*n* = 42) and uninvolved breast tissue samples that were adjacent to TNBC primary tumors (*n* = 21) upon the GPL11154 platform (Illumina HiSeq 2000).

Conscious of potential biases inherent to any statistical approach, a distinct analytical method was deliberately chosen for the GSE58135 RNA-seq dataset compared to the OPLS analysis employed on the GSE38959 microarray dataset. The GEO2R tool, employing Benjamini & Hochberg normalization, was utilized for the GSE58135 analysis. This analysis identified DEGs based on a stringent threshold of adjusted *p* value < 0.01 and absolute Log2 FC exceeding 1.585.

Subsequently, a Venn diagram tool was employed to compare the DEGs obtained from both platforms. This facilitated the identification of genes consistently modulated across microarray and RNA-seq analyses. By leveraging distinct technological platforms and analytical methods, this cross-platform approach mitigated platform-specific and method-specific biases, thereby strengthening confidence in the validity of the results.

### 2.7. Expression Analysis of Prognostic Markers in TNBC Versus Non-TNBC Samples

An investigation into the expression levels of prognostic markers in TNBC tissues compared to control samples was undertaken. This exploration was facilitated by utilizing the well-established bc-GenExMiner V5.0 online platform. This platform is a statistical tool designed explicitly for analyzing published and annotated breast cancer transcriptomic data, encompassing DNA microarray and RNA-seq technologies [28, 29].

Within bc-GenExMiner V5.0, an analysis of mRNA expression levels of prognostic genes in TNBC tissues relative to non-TNBC samples was performed. This analysis leveraged the entirety of available datasets, including Affymetrix (*n* = 5183), METABRIC (*n* = 1980), TCGA RNA-seq (*n* = 743), and SCAN-B RNA-seq (*n* = 3678).

## 3. Results

### 3.1. Identification of DEGs in TNBC Through Microarray Analysis

**Figure 1:**
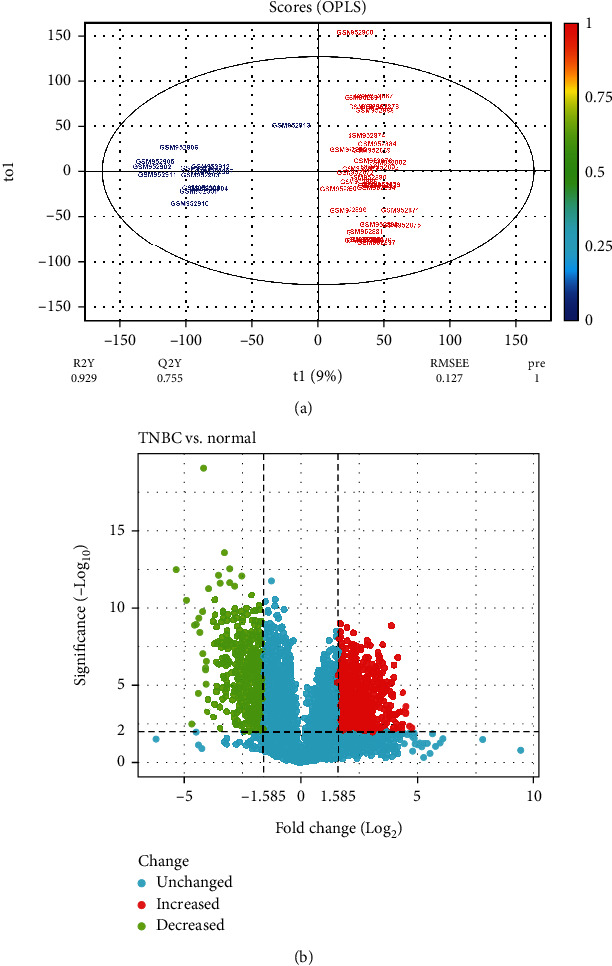
(a) The score plot delineates the predictive (*x*-axis) and orthogonal (*y*-axis) components of the microarray data obtained from tissue samples, employing the OPLS model. (b) The volcano plot illustrates the differential expression of genes in triple-negative breast cancer compared to normal breast tissues. OPLS, orthogonal partial least squares.

The multivariate statistical analysis of the GEO38959 dataset, comprising 43 samples (30 TNBC, 13 healthy) and 45,015 variables, revealed significant findings. The OPLS model effectively discriminated TNBC observations from normal breast samples, as indicated by the following parameters: R2X, 0.169; R2Y, 0.929; Q2Y, 0.755 (Figure 1(a)). Consequently, a total of 942 DEGs, encompassing 693 overexpressed and 249 downexpressed genes, met the criteria of *p* value < 0.01 and |Log2 FC| > 1.585 when comparing TNBC tissues to healthy breast samples (Table S1). The average values were analyzed in cases where genes exhibited multiple probes. The volcano plot illustrating the GEO38959 dataset is depicted in Figure 1(b).

### 3.2. PPIN and Functional Analyses

The graphical representation of interrelationships among genes linked to TNBC was accomplished using the STRING database, ensuring a robust confidence score ≥ 0.7. Nodes devoid of interactions were eliminated from the PPIN, creating a Cytoscape graph comprising 610 nodes and 4473 edges. Structural analysis revealed 19 genes with a degree surpassing twice the mean, along with betweenness and closeness values exceeding the average node features. Consequently, these nodes were identified as hub proteins within the PPIN associated with the tumorigenesis in TNBC, as detailed in Table 1. The average values for degree, betweenness, and closeness centrality were 14.666, 0.014, and 0.307, respectively.

**Figure 2:**
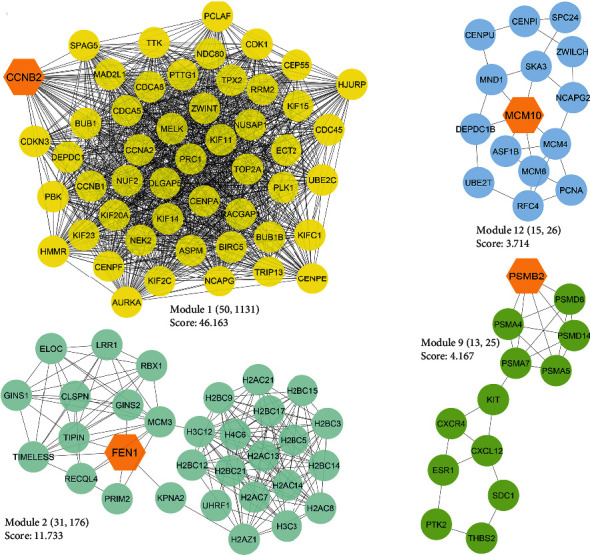
Four modules were identified using the MCODE plugin in a PPIN based on the DEGs in TNBC. The hexagons represent the seed nodes in each module. MCODE, molecular complex detection complex; PPIN, protein–protein interaction network; DEG, differentially expressed genes; TNBC, triple-negative breast cancer.

Four distinct modules were identified within the PPIN, each comprising more than 10 genes and possessing an MCODE score surpassing three. These clusters are denoted as Numbers 1, 2, 9, and 12, with Module 1 being the most substantial, encompassing 50 genes and 1131 edges (Figure 2).

**Figure 3:**
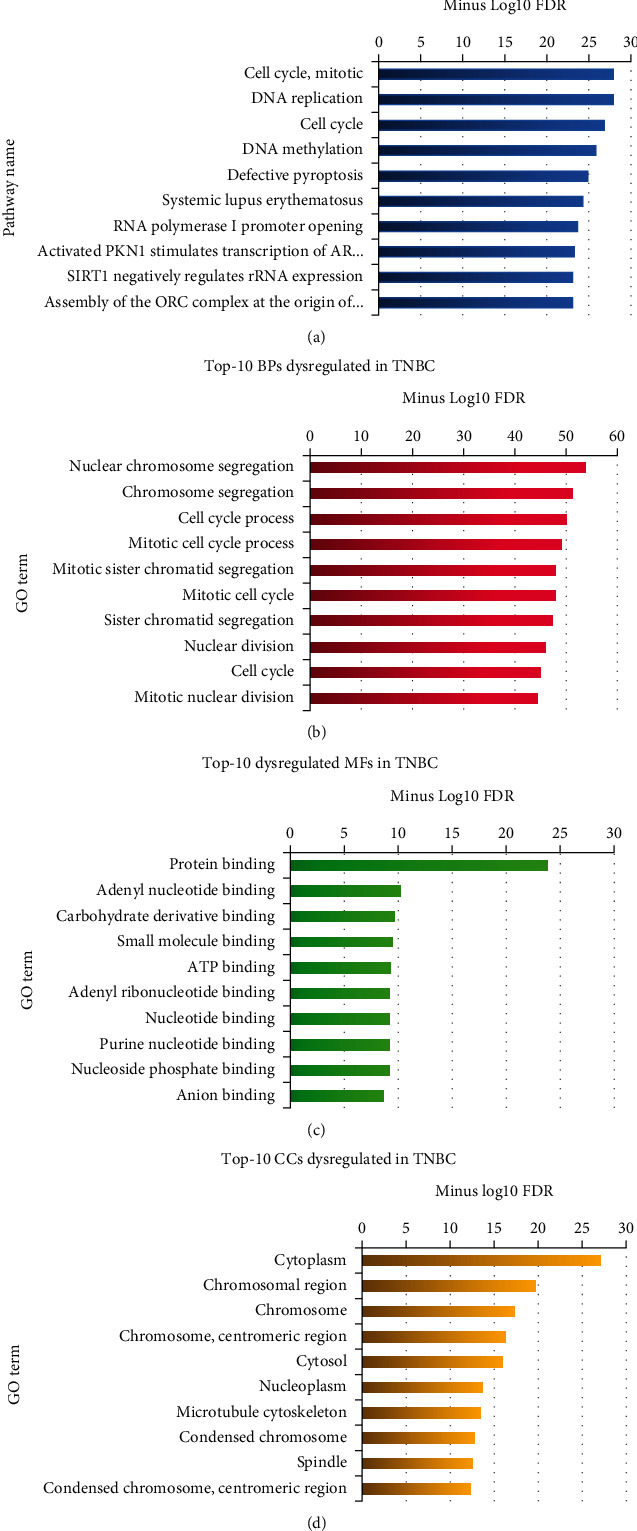
Top 10 (a) pathways, (b) biological processes, (c) molecular functions, and (d) cellular components involved in TNBC. TNBC, triple-negative breast cancer.

Functional analysis was then imposed on clusters to reveal pathways and biological processes exhibiting significant dysregulation in TNBC. The complete documentation of pathways and biological processes is meticulously presented in Tables S2 and S3, respectively. However, the top 10 terms are demonstrated in Figures 3(a) and 3(b) for a concise overview. Significantly, beyond pathways and biological processes linked to the mitotic division and cell cycle, it is noteworthy that pathways such as “activated PKN1 stimulates transcription of AR–regulated genes KLK2 and KLK3” (REAC: R-HSA-5625886) and “SIRT1 negatively regulates rRNA expression” (REAC: R-HSA-427359) demonstrated considerable enrichment in TNBC progression.

To elucidate the molecular functions and cellular components undergoing substantial perturbations in TNBC, a total of 943 DEGs were input into g:Profiler. The analysis unveiled that “protein binding” (GO: 0005515), “adenyl nucleotide binding” (GO: 0030554), and “carbohydrate derivative binding” (GO: 0097367) emerged as the most significantly enriched molecular functions in TNBC. Additionally, “cytoplasm” (GO: 0005737) and “chromosomal region” (GO: 0098687) were found to be the primary cellular components undergoing significant dysregulation in TNBC. Figures 3(c) and 3(d) illustrate the top 10 affected molecular functions and cellular components in TNBC, respectively. Tables S4 and S5 offer a detailed list of these enriched functions and components.

### 3.3. Survival and Boxplot Analyses

**Figure 4:**
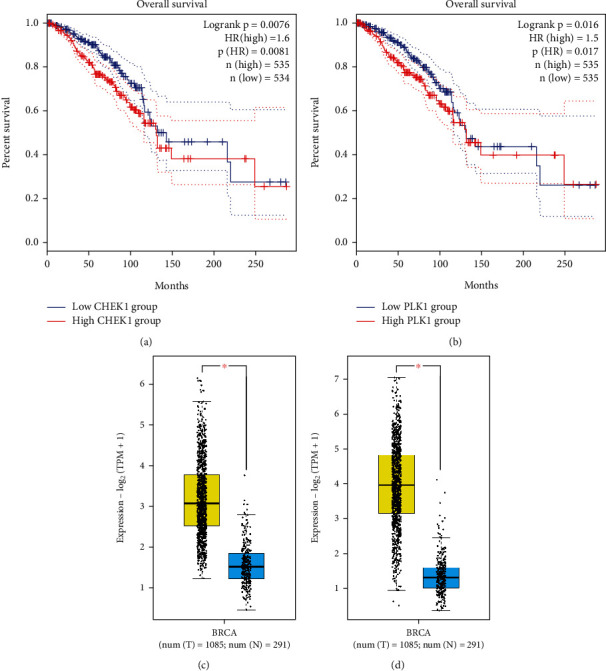
The prognostic significance of (a) CHEK1 and (b) PLK1 was notably indicated in patients diagnosed with breast invasive carcinoma. Kaplan–Meier survival curves were employed for graphical representation, with the *X*-axis indicating the survival time of breast invasive carcinoma patients and the *Y*-axis representing the corresponding survival probability. Additionally, the dotted lines in the graph delineate the 95% confidence intervals, offering a measure of the statistical reliability associated with the observed survival probabilities. The boxplot analysis, utilizing data from the GEPIA2 database, provides insights into the gene expression patterns of prognostic genes in breast invasive carcinoma. The analysis was conducted on a dataset comprising 1085 breast invasive carcinoma samples (highlighted in yellow) and 291 normal tissues (depicted in blue). These findings emphasize the differential gene expression of these markers, highlighting their potential role in breast invasive carcinoma, as cancerous samples consistently exhibit elevated expression levels compared to normal tissues for (c) CHEK1 and (d) PLK1.

The prognostic significance of the identified hub genes in TNBC was evaluated using Kaplan–Meier's curves generated through the GEPIA2 database. Among the 19 hub genes, significant associations were observed between upregulated expression of CHEK1 and PLK1 and poorer prognosis in patients with breast invasive carcinoma (Figures 4(a) and 4(b)). This association was supported by statistically significant results in the Log-rank test and HR *p* values (both < 0.05). No statistically significant correlations were found between dysregulation of the remaining hub genes and patient prognosis (Log-rank test or HR *p* values > 0.05).

Gene expression evaluation of CHEK1 and PLK1 was performed using boxplot analysis, utilizing data from the GEPIA2 database. The results of the boxplot analysis indicated overexpression of CHEK1 and PLK1 in breast invasive carcinoma compared to healthy controls, aligning with our findings (Figures 4(c) and 4(d)).

### 3.4. Upstream Regulators in TNBC

**Figure 5:**
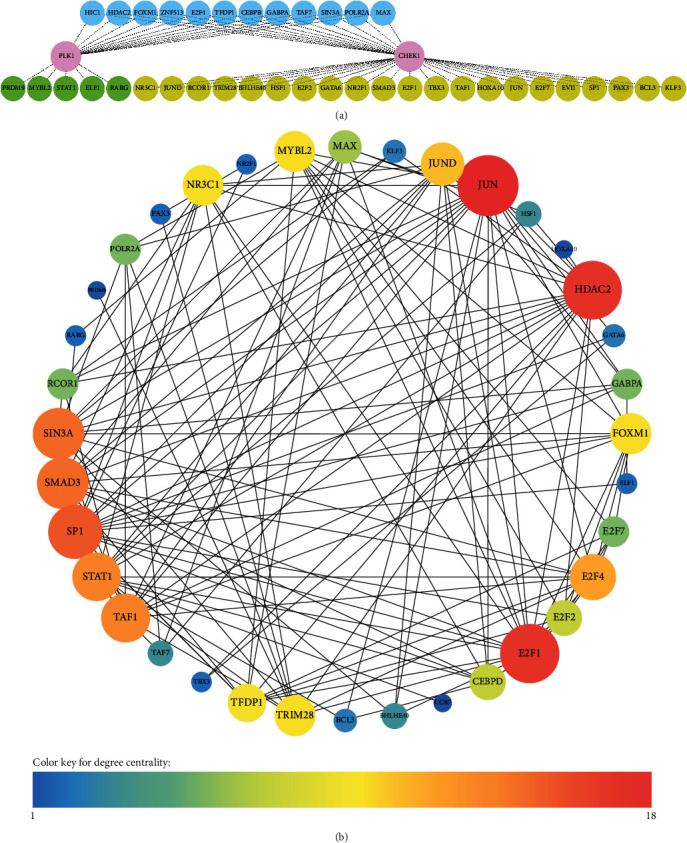
(a) A gene regulatory network encompassed negative markers in TNBC and their upstream regulators. (b) A protein interaction map was generated to visually illustrate the interactions between transcription factors regulating negative markers in TNBC. TNBC, triple-negative breast cancer.

To identify upstream regulators accountable for the expression of negative markers in TNBC, a GRN was meticulously constructed utilizing the iRegulon plugin, as depicted in Figure 5(a). Significance was attributed to transcription factors demonstrating an NES surpassing three. As a result, 38 genes were pinpointed as potential regulators of CHEK1 and/or PLK1, and their details are outlined in Table 2.

A novel protein interaction map (PIM) was systematically constructed, employing the identified transcription factors. The primary objective of this map was to visually illustrate the intricate interactions between these genes and investigate the most significant transcription factors among regulators, focusing on degree centrality. The graphical representation of this network is presented in Figure 5(b).

Regarding the NES, E2F7 exhibited the highest score at 14.09, directly influencing CHEK1 expression. Among regulators affecting CHEK1 and PLK1, GABP1 emerged as the most significant, with an NES of 13.477. Additionally, regarding degree centrality within the PIM, JUN demonstrated the highest number of connections among other regulators.

### 3.5. Concordant DEGs Across Microarray and RNA-Seq Platforms in TNBC

Analysis of the independent RNA-seq dataset GSE58135 using the GEO2R analysis tool identified a total of 3895 DEGs in TNBC tissues compared to healthy control samples (FDR < 0.01 and |Log2 FC| > 1.585) (Table S6). Among these DEGs, 2370 genes were upregulated, while 1525 were downregulated.

**Figure 6:**
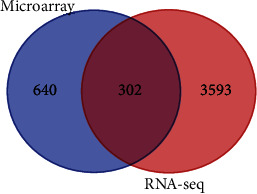
Identifying common DEGs in TNBC compared to healthy controls from microarray (GSE38959) and RNA-sequencing (GSE58135) datasets. A total of 942 and 3895 DEGs were indicated in TNBC versus healthy control samples by analyzing microarray and RNA-seq datasets, respectively. Of these, 302 were common between the two platforms. DEG, differentially expressed genes; TBNC, triple-negative breast cancer.

Notably, 302 genes exhibited overlap between the DEGs identified in the microarray dataset GSE38959 and the RNA-seq dataset GSE58135 (Figure 6 and Table 3). This overlap serves to strengthen the validity of the initial findings.

### 3.6. Expression Analysis of CHEK1 and PLK1 in TNBC Versus Non-TNBC Samples

The publicly available bc-GenExMiner V4.8 web–based tool was employed to investigate the specific expression patterns of CHEK1 and PLK1 in TNBC compared to other breast cancer subtypes. This database integrates gene expression data from various studies, facilitating robust comparative analyses across different breast cancer subtypes. The tool utilizes statistical methods to compare gene expression between groups and generates boxplots and statistical significance values.

**Figure 7:**
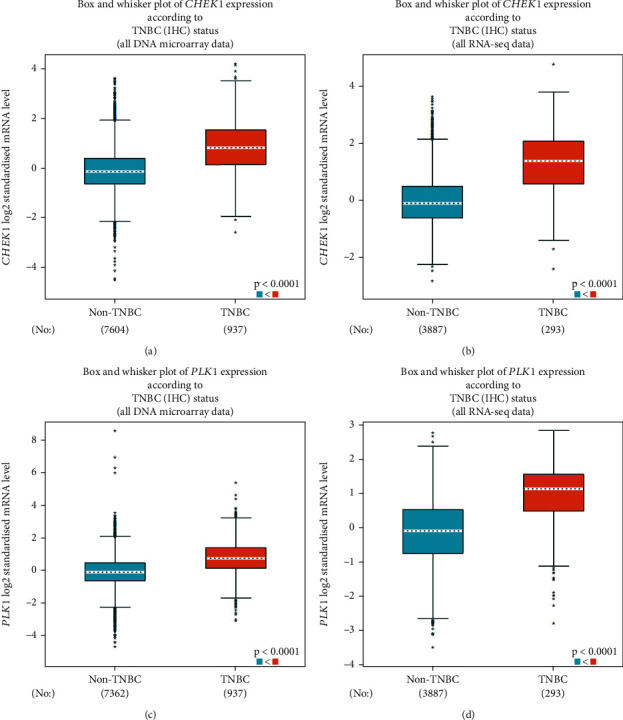
The expression patterns of CHEK1 and PLK1 were evaluated in TNBC tissues compared to other breast cancer subtypes based on two platforms, including microarray and RNA-seq datasets. This was performed using the bc-GenExMiner V4.8 database. Boxplot analyses demonstrated significantly higher expression of CHEK1 in TNBC samples (red) compared to non-TNBC tissues (blue) based on (a) microarray and (b) RNA-sequencing datasets. Similarly, PLK1 expression was also elevated in TNBC samples, as evidenced by the boxplots for both (c, d) microarray and RNA-sequencing data.

This analysis sought to confirm the specific upregulation of CHEK1 and PLK1 in TNBC, as suggested by the initial microarray findings and cross-platform verification analysis, and to contextualize their expression within the broader landscape of breast cancer subtypes. The results obtained from this analysis serve as further evidence supporting the potential role of CHEK1 and PLK1 as biomarkers or therapeutic targets, specifically in TNBC. The microarray and RNA-seq data indicated a statistically significant upregulation (*p* value < 0.0001) of these genes in TNBC tissues compared to non-TNBC samples (Figure 7), suggesting their potential involvement in TNBC development or progression.

## 4. Discussion

Breast cancer constitutes a pervasive malignancy afflicting women worldwide. Approximately 15% of all breast cancer cases are categorized as TNBC. Patients grappling with TNBC encounter a therapeutic challenge, being ineligible for endocrine or HER2-targeted therapies, thus making chemotherapy the primary avenue for treatment [30, 31]. This underscores the compelling necessity to explore and identify innovative therapeutic alternatives that have the potential to yield favorable prognoses for individuals diagnosed with TNBC.

The current study's findings highlight the substantial involvement of CHEK1 and PLK1 in shaping the prognosis of TNBC patients. Elevated expression levels of these markers are associated with unfavorable outcomes in breast–invasive carcinoma individuals. In addition, the present results revealed a significant enrichment of pathways and biological processes related to the mitotic cell cycle, DNA replication, and nuclear division in TNBC tumorigenesis. Notably, CHEK1 and PLK1, both extensively studied for their crucial roles in cell cycle regulation, emerge as potential therapeutic targets in oncology. CHEK1, a serine/threonine-protein kinase, is pivotal in the DNA damage response, acting as a cell cycle checkpoint regulator that halts progression at the G2/M phase in response to DNA damage, thus allowing for repair before mitosis **[**32**]**.

Similarly, PLK1, another serine/threonine-protein kinase, is essential for multiple mitotic stages, including spindle formation, chromosome segregation, and cytokinesis **[**33**]**. The upregulation of these genes in TNBC supports the observed enrichment of mitosis and DNA replication pathways. Clinical trials have demonstrated the effectiveness of PLK1 inhibitors in reducing tumor growth, although challenges such as resistance mechanisms and off-target effects remain. For instance, a randomized controlled trial involving advanced solid tumors showed promising antitumor activity of the PLK1 inhibitor BI 2536 but highlighted hematologic toxicities, underscoring the need for selective targeting strategies [34, 35]. Conversely, studies involving ovarian cancer patients have shown that combining CHEK1 inhibitors with standard chemotherapy improved progression-free survival, suggesting potential for therapeutic synergy **[**36**]**.

In a study by Yu et al. [37], a weighted gene coexpression network analysis (WGCNA) conducted on a dataset of breast cancer patients identified CHEK1 as a hub gene associated with the progression of breast cancer, particularly in the luminal A subtype. Furthermore, Wu et al. [38] reported higher mRNA levels of CHEK1 in breast cancer tissues compared to noncancerous tissues, correlating with clinical stages and overall survival in breast cancer patients. Immunohistochemistry analysis confirmed the overexpression of CHEK1 protein in breast cancer tissues, suggesting potential diagnostic and prognostic significance for CHEK1 in breast cancer. Additionally, in a study by Yang et al. [39], investigating the expression of RNF126, an E3 ligase, in invasive breast cancer, it was found that RNF126 was highly expressed in intrusive breast cancer tissues and was an independent predictor of poor prognosis. The study demonstrated that breast cancer cells expressing elevated levels of RNF126 exhibited heightened replication stress and increased sensitivity to CHEK1 inhibitors, providing further insights into the intricate associations between CHEK1, RNF126, and breast cancer outcomes.

PLK1 has emerged as a promising therapeutic target in breast cancer, particularly in TNBC, where successful molecular-targeted therapies are currently lacking [40]. A study by Ueda et al. [40] employed siRNA–mediated knockdown screening, identifying PLK1 as a potential therapeutic target for TNBC. Knockdown of PLK1 induced G2/M arrest and apoptosis in multiple TNBC cell lines. Moreover, PLK1 exhibited significant overexpression in TNBC patient tissues compared to normal mammary glands and benign breast tumors, as reported by Ueda et al. [40]. Ren et al. [41] identified PLK1 as a hub gene associated with TNBC, and subsequent cell and animal experiments confirmed that PLK1 promotes the proliferation, invasion, migration, and clone formation of breast cancer cells. Additionally, Patel et al. [42] demonstrated the antiproliferative and antimigratory properties of a novel allosteric inhibitor targeting PLK1, RK-10, in TNBC cells. Treatment with RK-10 reduced phospho-PLK1 levels, inhibited cell viability, attenuated wound healing, and induced cell cycle arrest in TNBC cells. Combining insights from Giordano et al. [43], the synergistic effects of PLK1 inhibitors with taxanes in TNBC cells and in vivo models have been observed. ATP-competitive PLK1 inhibitors, when combined with taxanes, demonstrated a reduction in clonogenic potential and tumorsphere formation in TNBC cells. In a xenograft model, combining a PLK1 inhibitor with paclitaxel significantly decreased tumor volume compared to single-agent paclitaxel. These collective findings underscore the potential efficacy of combining PLK1 inhibitors as a promising strategy for the treatment of TNBC.

As per the GRN analysis, GABP1 has been identified as the most significant upstream regulator influencing CHEK1 and PLK1. Additionally, JUN has been noted for demonstrating the highest number of interactions with other regulators in the network. These findings underscore the central regulatory roles of GABP1 and the extensive interactive network involving JUN in the modulation of critical elements such as CHEK1 and PLK1.

GABP1, also known as GABPB1, has been implicated in various cancer types, such as non-small cell lung cancer (NSCLC). In a study conducted by Sun et al. [44], the focus was on HOMER3, a protein–promoting NSCLC growth and metastasis, which was found to upregulate the expression of GABPB1. GABPB1, in turn, is involved in mitochondrial biogenesis and function. Sun et al. [44] demonstrated that reduced levels of HOMER3 and GABPB1 resulted in mitochondrial dysfunction, leading to decreased proliferation and invasive activity of lung cancer cells. In a separate investigation by Xie et al. [45], a long noncoding RNA named GABPB1-IT1 was identified and observed to be downregulated in NSCLC tissues. The diminished expression of GABPB1-IT1 was correlated with a poor prognosis in NSCLC patients. Functional analysis revealed the involvement of GABPB1-IT1 in regulating cell cycle-associated biological processes. This study provides valuable insights into the potential of GABPB1-IT1 as both a prognostic biomarker and a prospective therapeutic target in the context of NSCLC treatment. It could be suggested that similar mechanisms may underlie the role of GABPB1 in both NSCLC and TNBC, although confirmation through further research is warranted.

Gao et al. [46] investigated the inhibitory effects of icariin (ICA) on TNBC through the JNK/c-Jun signaling pathway. Using network pharmacology and in vitro experiments, they demonstrated ICA's dose-dependent inhibition of TNBC cell functions, including proliferation, migration, and invasion. ICA induced redox-induced apoptosis and suppressed vital proteins in the JNK/c-Jun pathway, suggesting its potential as an effective inhibitor of TNBC cell proliferation and invasion. Furthermore, emphasizing the role of JUN as a critical protein in the JNK/c-Jun pathway underscores its potential as a target in treating TNBC patients. This insight provides a promising avenue for therapeutic intervention in TNBC.

Gene set enrichment analysis revealed the involvement of pathways such as “activated PKN1 stimulates transcription of AR (androgen receptor) regulated genes KLK2 and KLK3” (REAC: R-HSA-5625886) and “SIRT1 negatively regulates rRNA expression” (REAC: R-HSA-427359) in the progression of TNBC.

According to a study by Zhang et al. [47], the significance of PAK1 (serine/threonine-protein kinase N1 (PKN1)) in TNBC is noteworthy. The dual-targeting PAK1/HDAC6 inhibitor, ZMF-23, demonstrated potent inhibitory activity against PAK1 and HDAC6, exhibiting antiproliferative effects in TNBC cells. ZMF-23 modulated PAK1-tubulin/HDAC6-stathmin signaling, inducing changes in microtubule structure, cell cycle arrest, apoptosis, and necroptosis. This suggests that PAK1 plays a crucial role in energy metabolism and epigenetic modification in TNBC. Targeting PAK1 emerges as a promising therapeutic strategy for this aggressive subtype of breast cancer.

Jiang et al. [48] demonstrated that NAD+ supplementation significantly limits TNBC metastasis. Jiang et al. [48] reported that activating the SIRT1-p66Shc axis by NAD+ supplementation inhibited epithelial-mesenchymal transition (EMT), a critical step in cancer metastasis. According to the study by Jiang et al. [48], it can be suggested that SIRT1 (NAD-dependent protein deacetylase SIRT1) plays a significant role in the EMT process in TNBC.

Several novel aspects were incorporated into this study, expanding upon prior bioinformatic investigations of TNBC. While earlier works by Chen et al. [49], Bissanum et al. [50], and Chen, Cai, and Wang [51] primarily concentrated on the identification of DEGs and their prognostic value, a comprehensive GRN was constructed in our approach. This network analysis facilitated the identification of critical genes like CHEK1 and PLK1 and their upstream regulators, including GABPB1 and JUN. Consequently, this approach offers a more holistic understanding of the regulatory mechanisms underlying TNBC progression. Furthermore, our study employed a robust multivariate statistical approach (OPLS) for DEG identification, differentiating it from past studies that primarily relied on methods like WGCNA or standard differential expression analysis. This methodological distinction likely enhances the findings' accuracy and reliability. Pathway analysis was conducted, revealing novel insights into TNBC progression. Pathways such as “activated PKN1 stimulates transcription of AR–regulated genes KLK2 and KLK3” and “SIRT1 negatively regulates rRNA expression” were highlighted, which were not emphasized in previous studies. This sheds light on potentially significant mechanisms in TNBC development. While genes like FERMT1, CENPW, HORMAD1, BUB1, and CCNA2 were identified as potential biomarkers in prior works, our study's focus on CHEK1 and PLK1 offers new perspectives on therapeutic targets. This shift in focus could lead to the development of more effective treatment strategies for TNBC. This study adopted a more comprehensive systems biology approach to understanding TNBC by integrating PPI networks, survival analysis, and gene regulatory networks. This approach can potentially pave the way for developing novel targeted therapies for this aggressive cancer subtype.

While this study provides valuable insights into the molecular landscape of TNBC through comprehensive bioinformatics analyses, it is essential to acknowledge its limitations. Although we employed multiple bioinformatics tools and platforms, including cross-platform verification and gene expression analysis using bc-GenExMiner, to confirm the overexpression of CHEK1 and PLK1 in TNBC, these findings are primarily based on in silico analyses of publicly available datasets. While such approaches offer robust statistical power and the ability to integrate large-scale data, they may not fully capture the biological complexity and heterogeneity of TNBC. Experimental validation using local patient samples is crucial to enhancing our results' quality and reliability. This could involve techniques such as quantitative PCR, Western blotting, or immunohistochemistry to confirm the overexpression of CHEK1 and PLK1 at both mRNA and protein levels in TNBC tissues compared to normal breast tissues and other breast cancer subtypes. Additionally, functional studies in TNBC cell lines and animal models would provide valuable insights into the biological roles of these genes in TNBC progression. These experimental validations would substantiate our bioinformatics findings and pave the way for translating these results into potential diagnostic or therapeutic applications for TNBC patients.

## 5. Conclusion

This comprehensive system biology approach to understanding TNBC has provided valuable insights into the molecular landscape of this aggressive cancer subtype. CHEK1 and PLK1 were identified as crucial prognostic markers and potential therapeutic targets in TNBC. Their significance in TNBC progression is underscored by their overexpression, confirmed through multiple bioinformatics approaches and datasets. Identifying GABPB1 and JUN as critical upstream regulators offers a deeper understanding of the regulatory mechanisms governing CHEK1 and PLK1 expression in TNBC. This knowledge opens up new avenues for therapeutic interventions that could potentially target these regulatory pathways. This study contributes to the growing understanding of TNBC biology and presents CHEK1, PLK1, and their regulatory network as promising targets for therapeutic intervention. As the complex molecular architecture of TNBC continues to be unravelled, integrative approaches like the one employed in this study will be crucial in identifying effective strategies to combat this challenging disease.

## Figures and Tables

**Table 1:** Nineteen nodes were assigned as hub genes in a PPIN associated with TNBC pathogenesis. These genes have been sorted based on their degree score.

**Gene symbol**	**Degree**	**Betweenness**	**Closeness**
CDK1	131	0.072	0.388
CCNB1	112	0.04	0.378
CCNA2	108	0.018	0.36
PLK1	91	0.025	0.354
KIF11	90	0.017	0.344
CENPA	89	0.018	0.351
CDC45	88	0.024	0.352
RAD51	79	0.082	0.373
CHEK1	77	0.038	0.365
RRM2	72	0.028	0.348
NDC80	68	0.016	0.342
AURKA	67	0.069	0.362
H4C6	65	0.038	0.348
KIF23	64	0.015	0.334
H3C12	63	0.112	0.375
TYMS	55	0.047	0.342
FN1	31	0.104	0.325
E2F1	31	0.034	0.361
BARD1	31	0.024	0.345

Abbreviations: PPIN, protein–protein interaction network; TNBC, triple–negative breast cancer.

**Table 2:** A total of 38 transcription factors significantly affect CHEK1 and/or PLK1 expression.

**TF**	**NES**	**Targets**
E2F7	14.092	CHEK1
E2F1	13.626	CHEK1
GABPA	13.477	CHEK1, PLK1
ELF1	13.286	PLK1
STAT1	13.01	PLK1
RARG	12.968	PLK1
HIC1	12.098	CHEK1, PLK1
KLF3	11.737	CHEK1
PAX3	10.783	CHEK1
SP1	10.74	CHEK1
ZNF513	10.252	CHEK1, PLK1
TFDP1	8.262	CHEK1, PLK1
PRDM9	7.494	PLK1
HSF1	7.197	CHEK1
BHLHE40	5.451	CHEK1
JUN	5.399	CHEK1
POLR2A	5.241	CHEK1, PLK1
SMAD3	5.223	CHEK1
FOXM1	5.145	CHEK1, PLK1
TAF1	5.127	CHEK1
HDAC2	5.022	CHEK1, PLK1
HOXA10	5.004	CHEK1
RCOR1	4.978	CHEK1
E2F4	4.864	CHEK1, PLK1
SIN3A	4.864	CHEK1, PLK1
MYBL2	4.803	PLK1
JUND	4.768	CHEK1
NR3C1	4.619	CHEK1
BCL3	4.339	CHEK1
E2F2	4.076	CHEK1
GATA6	4.076	CHEK1
TRIM28	3.875	CHEK1
CEBPD	3.682	CHEK1, PLK1
TAF7	3.533	CHEK1, PLK1
NR2F1	3.498	CHEK1
MAX	3.463	CHEK1, PLK1
TBX3	3.27	CHEK1
EVI1	3.06	CHEK1

Abbreviation: NES, normalized enrichment score.

**Table 3:** A total of 302 differentially expressed genes were common in two datasets, including GSE38959 and GSE58135.

**Datasets**	**Total**	**Genes**
Microarray (GSE38959) and RNA-seq (GSE58135)	302	KLHL13 TMSB10 CCNB1 PDE1C GEN1 NOXO1 RELT CCNE1 EN1 IRS1 FGF1 KIF14 SMC6 MAD2L1 CRYBA1 SEMA3G NTN4 SQLE MCM10 CBS IL20RA TYMS MELK VEGFA NDC80 MME SOCS3 OIP5 CCNA2 TMEM132A LAMA3 SCGB1D2 STMN1 CKS2 KIF23 DEPDC1 ZNF695 SLC39A4 LMNB1 NRN1 SPAG5 NTRK2 PAX6 CXCL2 CHML CCNB2 PRC1 FUT3 PIF1 LRP8 APOLD1 SPATA18 CCNE2 LRRCC1 GRPR MCM4 GRIK3 HBB LIN9 PGR SRPX ADAM8 CDCA2 EXO1 PDGFRA CSTB CENPI CENPK BCL6B FAM47E CX3CR1 SERPINA5 NEK2 FANCD2 AFF3 CDC25A XRCC2 ERBB4 PTN DEPDC1B ITIH5 CLDN11 RERG EZH2 CHEK1 KIF11 KIF18B GPRASP2 SCN4A POU4F1 KIFC1 FADS2 TSHZ2 LTBP2 CHL1 KIF21A EDNRB SCD MYH11 EGFLAM GSDMC PPP1R16A PYCR1 CENPA RNFT2 CDCA7 PLK4 RFC4 BUB1 POLQ EGR3 TRIP13 CDC7 NUP210 ABCG2 UBE2S INPP4B CDO1 IFI6 UBE2C MND1 SDC1 PSAT1 FOS CXCL12 RRM2 TOP2A HELLS FANCI CCL28 RAD54L SPARCL1 PKMYT1 KIF18A RHBDL2 RBMS3 FAM83D IL33 CIT SCUBE2 CHAF1B TMEFF1 STC2 SPARC IL20RB PPFIA4 THSD4 TRIM59 UHRF1 CDKN2A SCN2B RECQL4 MAMDC2 ADAMTS5 CDKN3 E2F3 CENPM CENPF NDN CDCA8 IGF1 TPX2 ANP32E GINS1 CNN1 KPNA2 ANLN BIRC5 CHRNA5 BOP1 RACGAP1 OXTR IKZF3 CLSTN2 CDC6 AURKA FANCA KCND3 KIT SCN4B BARD1 SH3BGRL2 RUNX1T1 IL4I1 ESR1 FMOD LRIG1 ZWINT NUF2 PDK4 NEK10 SAMD5 PTTG1 CDCA5 UBE2T SLC2A1 BCL2A1 CTTNBP2 ECT2 DIAPH3 GGH CENPE PLCH1 DKK3 PPP1R14B TACC3 PLAT MEX3A CLEC7A GPRIN1 CEP55 CBX7 RAI2 LRP2 SEMA6D GLDC POP1 DLGAP5 RAD51AP1 RNASE1 CLSPN NAV3 AK5 SLC35F2 NMU RSPO3 GRIA4 FAM111B TMTC1 AMIGO2 KCNJ8 GINS2 SPC24 DONSON E2F8 ARHGAP11A HMGB3 HOMER3 AGR3 SYNPO2 DACH1 PLK1 FOXC1 ASF1B E2F1 PIGR ZNF707 MAPT SMC4 DNALI1 ANO1 ZIC1 EDN3 BLM KIF2C TBX1 AGTR1 DCDC2 PBK TROAP TK1 PDE2A ASPM TMSB15A TMSB15B ACADSB AFAP1L2 INHBA GPR137C IGF2 ATAD2 SLC7A5 LIFR PFDN6 COL14A1 PRKD1 SLC16A3 ZNF367 WDHD1 KIF15 TMEM144 ITM2A BUB1B HJURP RNASEH2A DTL RAD51 HMMR CCDC150 RGS10 NPY2R KIF20A PPP1R3C SOX11 SEMA5A VIPR1 NRG1 TTK NCAPG BOLA2B TP63 NUSAP1

## Data Availability

The datasets used and/or analyzed during the current study are available from the corresponding author upon reasonable request.

## Supporting Information

Supporting Information Additional supporting information can be found online in the Supporting Information section. Table S1. The OPLS model indicated 943 DEGs in TNBC compared to healthy tissue with a criterion of *p* value < 0.01 and |Log2 FC| > 1.585. Table S2. Pathways involved in TNBC. Table S3. Biological processes involved in TNBC. Table S4. Molecular functions involved in TNBC. Table S5. Cellular components involved in TNBC. Table S6. The GEO2R indicated 3895 DEGs in primary TNBC compared to healthy tissues with a criterion of *p* value < 0.01 and |Log2 FC| > 1.585.
