# Supplementary material for: Potential Therapeutic Targets in Triple-Negative Breast Cancer Based on Gene Regulatory Network Analysis: A Comprehensive Systems Biology Approach
Source: Int J Breast Cancer. 2024 Oct 22;2024:8796102. doi: 10.1155/2024/8796102 (PMC11521586; doi:10.1155/2024/8796102)
Supplement: Supporting Information — Additional supporting information can be found online in the Supporting Information section. Table S1. The OPLS model indicated 943 DEGs in TNBC compared to healthy tissue with a criterion of p value < 0.01 and |Log2 FC| > 1.585. Table S2. Pathways involved in TNBC. Table S3. Biological processes involved in TNBC. Table S4. Molecular functions involved in TNBC. Table S5. Cellular components involved in TNBC. Table S6. The GEO2R indicated 3895 DEGs in primary TNBC compared to healthy tissues with a criterion of p value < 0.01 and |Log2 FC| > 1.585. [file 8796102.f1.docx]

**Supplementary Table 1.** The OPLS model indicated 943 DEGs in TNBC compared to healthy tissue with a criterion of p-value < 0.01 and |Log2 FC| > 1.585.

| **Symbol** | **p-value** | **FC** | **Log2.FC** | **\|Log2 FC\|** |
| --- | --- | --- | --- | --- |
| NMU | 0.00457 | 25.63 | 4.68 | 4.68 |
| FAM111B | 3.11E-05 | 20.59 | 4.36 | 4.36 |
| ABCC4 | 0.00299 | 20.33 | 4.35 | 4.35 |
| MGC12935 | 0.00397 | 19.33 | 4.27 | 4.27 |
| CDKN2A | 6.51E-04 | 18.03 | 4.14 | 4.14 |
| TPX2 | 1.66E-07 | 17.66 | 4.14 | 4.14 |
| CTHRC1 | 0.00291 | 17.11 | 4.10 | 4.10 |
| FN1 | 8.49E-03 | 16.67 | 4.05 | 4.05 |
| CCDC67 | 0.00695 | 16.31 | 4.03 | 4.03 |
| DEPDC1 | 4.94E-07 | 15.80 | 3.98 | 3.98 |
| FAM83D | 3.13E-06 | 15.75 | 3.98 | 3.98 |
| PAX6 | 0.0015 | 15.17 | 3.92 | 3.92 |
| BIRC5 | 4.21E-05 | 14.90 | 3.90 | 3.90 |
| HJURP | 5.94E-06 | 14.86 | 3.89 | 3.89 |
| ANLN | 1.46E-09 | 14.82 | 3.89 | 3.89 |
| SOX11 | 0.000216 | 14.51 | 3.86 | 3.86 |
| ZIC1 | 0.00102 | 13.65 | 3.77 | 3.77 |
| CENPA | 0.00032 | 13.53 | 3.76 | 3.76 |
| CDC45L | 0.000199 | 12.41 | 3.63 | 3.63 |
| CXorf61 | 0.00139 | 12.30 | 3.62 | 3.62 |
| KIF18A | 2.43E-05 | 12.05 | 3.59 | 3.59 |
| KIF20A | 9.80E-06 | 11.98 | 3.57 | 3.57 |
| MLF1IP | 7.86E-07 | 11.95 | 3.58 | 3.58 |
| CENPM | 0.00275 | 11.86 | 3.57 | 3.57 |
| SPC24 | 9.90E-05 | 11.50 | 3.52 | 3.52 |
| CDC6 | 3.75E-05 | 11.20 | 3.49 | 3.49 |
| CDC2 | 1.91E-05 | 11.18 | 3.47 | 3.47 |
| NEK2 | 3.98E-05 | 10.98 | 3.46 | 3.46 |
| CASC5 | 2.96E-07 | 10.65 | 3.41 | 3.41 |
| GLDC | 0.00656 | 10.58 | 3.40 | 3.40 |
| IL4I1 | 0.00518 | 10.56 | 3.40 | 3.40 |
| PRC1 | 3.86E-06 | 10.33 | 3.37 | 3.37 |
| SPC25 | 4.76E-05 | 10.30 | 3.36 | 3.36 |
| TTK | 1.42E-07 | 10.10 | 3.34 | 3.34 |
| LMNB1 | 1.47E-05 | 9.92 | 3.31 | 3.31 |
| TMEM56 | 0.00143 | 9.91 | 3.31 | 3.31 |
| C6orf150 | 6.43E-05 | 9.86 | 3.30 | 3.30 |
| CCNE1 | 8.20E-08 | 9.81 | 3.29 | 3.29 |
| DOLK | 0.0029 | 9.54 | 3.25 | 3.25 |
| EN1 | 0.0035 | 9.45 | 3.24 | 3.24 |
| KIF23 | 0.000152 | 9.35 | 3.22 | 3.22 |
| RAD54L | 2.61E-06 | 9.34 | 3.22 | 3.22 |
| KIFC1 | 0.000103 | 9.23 | 3.21 | 3.21 |
| GINS1 | 1.23E-06 | 9.23 | 3.21 | 3.21 |
| C18orf24 | 0.00275 | 9.21 | 3.20 | 3.20 |
| CENPK | 7.21E-07 | 9.11 | 3.19 | 3.19 |
| ECT2 | 3.61E-05 | 8.97 | 3.14 | 3.14 |
| PBK | 1.19E-05 | 8.96 | 3.16 | 3.16 |
| MICB | 0.00552 | 8.93 | 3.16 | 3.16 |
| THBS2 | 0.00015655 | 8.91 | 3.14 | 3.14 |
| GGH | 0.00181 | 8.90 | 3.15 | 3.15 |
| ZNF695 | 0.000214 | 8.85 | 3.15 | 3.15 |
| E2F1 | 1.43E-06 | 8.68 | 3.08 | 3.08 |
| FADS2 | 2.85E-05 | 8.65 | 3.11 | 3.11 |
| CDCA5 | 1.25E-06 | 8.63 | 3.11 | 3.11 |
| COL1A1 | 9.55E-03 | 8.59 | 3.10 | 3.10 |
| MELK | 1.91E-06 | 8.52 | 3.09 | 3.09 |
| HMMR | 0.00028 | 8.48 | 3.08 | 3.08 |
| ASPM | 2.81E-06 | 8.36 | 3.03 | 3.03 |
| PMEPA1 | 0.00054 | 8.31 | 3.05 | 3.05 |
| RHBDL2 | 0.0020635 | 8.25 | 2.88 | 2.88 |
| MND1 | 0.00277 | 8.23 | 3.04 | 3.04 |
| CENPF | 9.99E-06 | 8.18 | 3.00 | 3.00 |
| KIF14 | 7.23E-05 | 8.13 | 3.02 | 3.02 |
| GSDMC | 0.0052 | 8.09 | 3.02 | 3.02 |
| C6orf173 | 1.39E-04 | 8.04 | 3.01 | 3.01 |
| EPR1 | 3.37E-05 | 7.98 | 3.00 | 3.00 |
| MDK | 0.00207 | 7.98 | 3.00 | 3.00 |
| FAM64A | 1.66E-03 | 7.91 | 2.98 | 2.98 |
| NCAPG | 1.08E-06 | 7.89 | 2.98 | 2.98 |
| CDCA2 | 0.00183 | 7.89 | 2.98 | 2.98 |
| NUSAP1 | 2.63E-08 | 7.84 | 2.97 | 2.97 |
| POU4F1 | 0.00713 | 7.83 | 2.97 | 2.97 |
| RRM2 | 1.34E-05 | 7.81 | 2.83 | 2.83 |
| RBL1 | 5.78E-05 | 7.76 | 2.96 | 2.96 |
| HIST1H1A | 0.000941 | 7.73 | 2.95 | 2.95 |
| CDKN3 | 7.40E-06 | 7.67 | 2.94 | 2.94 |
| BUB1 | 9.10E-07 | 7.39 | 2.89 | 2.89 |
| PTTG1 | 0.000728 | 7.38 | 2.88 | 2.88 |
| CLEC7A | 0.00926 | 7.36 | 2.88 | 2.88 |
| UHRF1 | 1.65E-07 | 7.35 | 2.88 | 2.88 |
| FNDC1 | 0.00848 | 7.35 | 2.88 | 2.88 |
| GPRC5A | 5.44E-03 | 7.34 | 2.72 | 2.72 |
| CEP55 | 1.59E-05 | 7.33 | 2.87 | 2.87 |
| ZWINT | 4.22E-06 | 7.27 | 2.86 | 2.86 |
| UBE2C | 1.35E-08 | 7.18 | 2.84 | 2.84 |
| GPR137C | 0.00145 | 7.17 | 2.84 | 2.84 |
| HIST1H2AG | 2.73E-08 | 7.08 | 2.82 | 2.82 |
| SGOL2 | 4.63E-05 | 7.07 | 2.82 | 2.82 |
| CENPI | 0.000129 | 6.98 | 2.80 | 2.80 |
| KIF11 | 0.000118695 | 6.95 | 2.76 | 2.76 |
| HRASLS | 0.00339 | 6.91 | 2.79 | 2.79 |
| UBE2T | 4.21E-07 | 6.87 | 2.78 | 2.78 |
| EZH2 | 4.01E-06 | 6.86 | 2.78 | 2.78 |
| TFDP3 | 3.60E-06 | 6.86 | 2.78 | 2.78 |
| FADS1 | 7.68E-05 | 6.85 | 2.76 | 2.76 |
| KIF2C | 3.06E-06 | 6.85 | 2.78 | 2.78 |
| SMC4 | 0.000061677 | 6.84 | 2.71 | 2.71 |
| FANCA | 0.0003741 | 6.83 | 2.77 | 2.77 |
| DTL | 8.68E-05 | 6.75 | 2.75 | 2.75 |
| NDC80 | 4.15E-04 | 6.72 | 2.62 | 2.62 |
| CCNB1 | 1.71E-07 | 6.72 | 2.75 | 2.75 |
| C15orf42 | 0.000452 | 6.67 | 2.74 | 2.74 |
| C13orf3 | 3.20E-05 | 6.61 | 2.72 | 2.72 |
| COL1A2 | 0.008768889 | 6.60 | 2.72 | 2.72 |
| VANGL1 | 1.12E-05 | 6.60 | 2.72 | 2.72 |
| EXO1 | 3.85E-04 | 6.56 | 2.68 | 2.68 |
| CDCA7 | 0.000365 | 6.56 | 2.71 | 2.71 |
| C18orf56 | 0.00184 | 6.55 | 2.71 | 2.71 |
| LRP8 | 2.13E-05 | 6.55 | 2.71 | 2.71 |
| SCD | 0.002593 | 6.55 | 2.70 | 2.70 |
| KIAA0101 | 0.000143 | 6.45 | 2.69 | 2.69 |
| HIST1H3B | 4.18E-06 | 6.42 | 2.68 | 2.68 |
| TMPO | 0.00193 | 6.40 | 2.68 | 2.68 |
| HIST1H2AM | 0.000192018 | 6.39 | 2.61 | 2.61 |
| OIP5 | 3.16E-07 | 6.36 | 2.67 | 2.67 |
| CDCA8 | 3.10E-05 | 6.36 | 2.67 | 2.67 |
| ASB9 | 0.00105 | 6.32 | 2.66 | 2.66 |
| HIST1H3I | 0.000156 | 6.28 | 2.65 | 2.65 |
| GSG2 | 0.0012625 | 6.26 | 2.65 | 2.65 |
| LOX | 0.005871111 | 6.25 | 2.64 | 2.64 |
| HIST2H2AA4 | 2.72E-05 | 6.24 | 2.63 | 2.63 |
| E2F8 | 0.000276 | 6.24 | 2.64 | 2.64 |
| HN1 | 0.000125 | 6.24 | 2.63 | 2.63 |
| CHRNA5 | 0.000296 | 6.24 | 2.64 | 2.64 |
| TOP2A | 5.20E-05 | 6.22 | 2.64 | 2.64 |
| TK1 | 0.00171 | 6.20 | 2.63 | 2.63 |
| PIF1 | 0.000243 | 6.19 | 2.63 | 2.63 |
| BRI3BP | 1.55E-05 | 6.17 | 2.63 | 2.63 |
| CENPN | 0.001511 | 6.16 | 2.57 | 2.57 |
| TROAP | 4.00E-05 | 6.11 | 2.61 | 2.61 |
| HIST1H2AE | 9.88E-06 | 6.09 | 2.61 | 2.61 |
| HIST1H2BG | 3.69E-08 | 5.96 | 2.57 | 2.57 |
| SERPINH1 | 0.00571 | 5.94 | 2.57 | 2.57 |
| MTHFD1L | 0.0057 | 5.93 | 2.57 | 2.57 |
| TMSB15A | 0.00505 | 5.91 | 2.56 | 2.56 |
| NRN1 | 0.00678 | 5.91 | 2.56 | 2.56 |
| RAD51AP1 | 7.53E-06 | 5.89 | 2.56 | 2.56 |
| FANCI | 0.00009954 | 5.86 | 2.55 | 2.55 |
| CDKN2B | 0.00703 | 5.86 | 2.55 | 2.55 |
| FABP5 | 0.00063875 | 5.85 | 2.54 | 2.54 |
| ELF4 | 0.000836 | 5.85 | 2.55 | 2.55 |
| LOXL2 | 0.007973 | 5.85 | 2.54 | 2.54 |
| RNASE1 | 0.0082 | 5.84 | 2.55 | 2.55 |
| RPL39L | 0.00191 | 5.83 | 2.54 | 2.54 |
| ORC6L | 1.98E-05 | 5.82 | 2.54 | 2.54 |
| PLOD2 | 0.000118 | 5.82 | 2.54 | 2.54 |
| SLC7A5 | 0.00291 | 5.79 | 2.49 | 2.49 |
| ZNF367 | 2.44E-04 | 5.79 | 2.53 | 2.53 |
| DEPDC1B | 0.000166 | 5.78 | 2.53 | 2.53 |
| CKS2 | 7.00E-07 | 5.75 | 2.52 | 2.52 |
| SLC16A3 | 8.99E-05 | 5.75 | 2.52 | 2.52 |
| CCNYL1 | 0.00497 | 5.75 | 2.52 | 2.52 |
| H2AFX | 0.000739 | 5.74 | 2.52 | 2.52 |
| TACC3 | 0.000204 | 5.73 | 2.52 | 2.52 |
| CSTB | 4.57E-05 | 5.70 | 2.51 | 2.51 |
| CDKN2C | 0.000688 | 5.70 | 2.51 | 2.51 |
| ATP1B3 | 0.000264 | 5.67 | 2.50 | 2.50 |
| HIST2H2AB | 1.96E-05 | 5.67 | 2.45 | 2.45 |
| DHCR7 | 0.00104 | 5.66 | 2.50 | 2.50 |
| AURKA | 0.000381 | 5.65 | 2.50 | 2.50 |
| GPC4 | 0.00717 | 5.63 | 2.49 | 2.49 |
| LOC387763 | 0.00515 | 5.59 | 2.48 | 2.48 |
| SPON2 | 0.00161 | 5.58 | 2.48 | 2.48 |
| HMGB3 | 3.35E-05 | 5.53 | 2.47 | 2.47 |
| COL5A2 | 0.00513 | 5.52 | 2.47 | 2.47 |
| CENPE | 3.15E-05 | 5.51 | 2.45 | 2.45 |
| UGT8 | 0.00295 | 5.47 | 2.45 | 2.45 |
| HIST1H2BH | 2.01E-06 | 5.47 | 2.45 | 2.45 |
| LY96 | 0.00935 | 5.46 | 2.45 | 2.45 |
| SLC2A1 | 0.00013 | 5.45 | 2.45 | 2.45 |
| IL32 | 0.00764 | 5.45 | 2.45 | 2.45 |
| ARHGAP11A | 0.000225295 | 5.44 | 2.44 | 2.44 |
| HIST1H2AL | 0.000166 | 5.41 | 2.44 | 2.44 |
| TRIP13 | 3.97E-05 | 5.40 | 2.43 | 2.43 |
| MRPL47 | 7.39E-06 | 5.39 | 2.43 | 2.43 |
| KIF15 | 5.64E-06 | 5.37 | 2.42 | 2.42 |
| NUDCD1 | 0.004808889 | 5.34 | 2.41 | 2.41 |
| SEPT3 | 0.00966 | 5.32 | 2.41 | 2.41 |
| CIT | 2.92E-05 | 5.31 | 2.41 | 2.41 |
| RAD51 | 3.67E-06 | 5.31 | 2.41 | 2.41 |
| TYMS | 0.000173 | 5.30 | 2.41 | 2.41 |
| KIF18B | 4.75E-05 | 5.30 | 2.41 | 2.41 |
| BLM | 0.000139 | 5.30 | 2.41 | 2.41 |
| FAM119A | 0.00032 | 5.29 | 2.40 | 2.40 |
| STMN1 | 5.35E-05 | 5.26 | 2.40 | 2.40 |
| MRPL13 | 0.000239 | 5.25 | 2.39 | 2.39 |
| GPRIN1 | 0.00331 | 5.24 | 2.39 | 2.39 |
| CBS | 0.00333 | 5.24 | 2.39 | 2.39 |
| SLC16A1 | 0.00235 | 5.23 | 2.39 | 2.39 |
| MCM10 | 1.34E-03 | 5.22 | 2.39 | 2.39 |
| SOAT1 | 5.01E-05 | 5.22 | 2.39 | 2.39 |
| ARMC9 | 0.00245 | 5.21 | 2.38 | 2.38 |
| CD99 | 4.42E-04 | 5.21 | 2.38 | 2.38 |
| XRCC2 | 0.000466 | 5.20 | 2.38 | 2.38 |
| RACGAP1 | 6.97E-07 | 5.19 | 2.38 | 2.38 |
| PLCH1 | 5.87E-04 | 5.18 | 2.26 | 2.26 |
| C20orf24 | 8.82E-07 | 5.18 | 2.37 | 2.37 |
| HIST1H2BI | 1.91E-07 | 5.17 | 2.37 | 2.37 |
| SLFN11 | 0.00237 | 5.17 | 2.37 | 2.37 |
| MCM2 | 1.06E-06 | 5.17 | 2.37 | 2.37 |
| RNFT2 | 0.0012815 | 5.15 | 2.33 | 2.33 |
| GRPEL2 | 0.000117 | 5.13 | 2.36 | 2.36 |
| MARS | 2.32E-06 | 5.13 | 2.36 | 2.36 |
| NUF2 | 0.000127 | 5.11 | 2.35 | 2.35 |
| CTSC | 6.16E-03 | 5.09 | 2.34 | 2.34 |
| BUB1B | 2.78E-07 | 5.06 | 2.34 | 2.34 |
| BCL6B | 0.000712 | 5.05 | 2.34 | 2.34 |
| THOC4 | 5.26E-06 | 5.05 | 2.34 | 2.34 |
| HIST1H2BM | 4.60E-07 | 5.03 | 2.33 | 2.33 |
| HIST1H2BJ | 5.95E-04 | 5.03 | 2.33 | 2.33 |
| TTYH3 | 0.00376 | 5.03 | 2.33 | 2.33 |
| B4GALT2 | 0.000158 | 5.01 | 2.32 | 2.32 |
| HIST1H1D | 1.05E-05 | 5.00 | 2.32 | 2.32 |
| CCNA2 | 2.63E-06 | 4.99 | 2.32 | 2.32 |
| CCDC150 | 0.00025 | 4.98 | 2.32 | 2.32 |
| FAM33A | 1.08E-04 | 4.95 | 2.29 | 2.29 |
| FOXD2 | 0.00133 | 4.95 | 2.31 | 2.31 |
| FUT3 | 0.00671 | 4.94 | 2.31 | 2.31 |
| MCM4 | 2.94E-06 | 4.94 | 2.30 | 2.30 |
| LOC54492 | 0.00988 | 4.92 | 2.30 | 2.30 |
| ATAD2 | 9.74E-05 | 4.91 | 2.29 | 2.29 |
| HIST1H2BB | 1.02E-06 | 4.89 | 2.29 | 2.29 |
| COL5A1 | 0.00975 | 4.88 | 2.29 | 2.29 |
| HIST1H2AB | 8.41E-07 | 4.88 | 2.29 | 2.29 |
| TAP1 | 0.00412 | 4.87 | 2.29 | 2.29 |
| HIST1H2BO | 2.23E-07 | 4.87 | 2.28 | 2.28 |
| CLSPN | 1.40E-06 | 4.82 | 2.27 | 2.27 |
| HPSE | 0.00755 | 4.80 | 2.26 | 2.26 |
| RAD54B | 5.40E-04 | 4.79 | 2.25 | 2.25 |
| TBX1 | 0.00839 | 4.77 | 2.25 | 2.25 |
| C16orf75 | 3.08E-06 | 4.77 | 2.25 | 2.25 |
| HIST1H2BN | 2.39E-08 | 4.76 | 2.25 | 2.25 |
| EPPK1 | 0.000241 | 4.75 | 2.25 | 2.25 |
| CHEK1 | 7.02E-05 | 4.74 | 2.24 | 2.24 |
| CCNE2 | 0.000499 | 4.72 | 2.24 | 2.24 |
| HIST1H2BL | 1.51E-07 | 4.70 | 2.23 | 2.23 |
| PDK1 | 0.000863 | 4.67 | 2.22 | 2.22 |
| NUB1 | 0.00502 | 4.67 | 2.22 | 2.22 |
| C16orf61 | 5.02E-05 | 4.66 | 2.22 | 2.22 |
| HIST1H2BE | 4.42E-08 | 4.65 | 2.22 | 2.22 |
| HIST1H2AJ | 1.87E-05 | 4.63 | 2.15 | 2.15 |
| GCUD2 | 9.20E-05 | 4.63 | 2.21 | 2.21 |
| IDH2 | 0.00197 | 4.62 | 2.21 | 2.21 |
| CHAF1B | 0.0010434 | 4.60 | 2.20 | 2.20 |
| IFI30 | 0.000925 | 4.60 | 2.20 | 2.20 |
| ACER3 | 0.00126 | 4.59 | 2.20 | 2.20 |
| STK38L | 0.00253 | 4.59 | 2.20 | 2.20 |
| HIST1H2BF | 1.63E-07 | 4.59 | 2.20 | 2.20 |
| PRDX4 | 1.77E-06 | 4.57 | 2.19 | 2.19 |
| CDC7 | 3.18E-05 | 4.57 | 2.19 | 2.19 |
| DSN1 | 5.81E-05 | 4.56 | 2.19 | 2.19 |
| HIST1H2AD | 1.60E-06 | 4.56 | 2.19 | 2.19 |
| HIST1H2BK | 0.000477 | 4.55 | 2.19 | 2.19 |
| HMGB3L1 | 3.27E-06 | 4.54 | 2.18 | 2.18 |
| CDKN2D | 0.00118 | 4.53 | 2.18 | 2.18 |
| ECE2 | 0.000134 | 4.53 | 2.18 | 2.18 |
| HIST2H2BE | 2.72E-08 | 4.52 | 2.18 | 2.18 |
| FOXC1 | 0.00719 | 4.52 | 2.18 | 2.18 |
| MLF1 | 4.25E-05 | 4.51 | 2.17 | 2.17 |
| PTTG2 | 0.000207 | 4.49 | 2.17 | 2.17 |
| TRIM59 | 0.000406 | 4.49 | 2.17 | 2.17 |
| GPSM2 | 0.00484 | 4.46 | 2.16 | 2.16 |
| PMCHL1 | 0.000332 | 4.45 | 2.15 | 2.15 |
| IKZF3 | 0.00423 | 4.45 | 2.15 | 2.15 |
| LMO4 | 0.0023 | 4.43 | 2.15 | 2.15 |
| ANKS6 | 0.00484 | 4.41 | 2.14 | 2.14 |
| BID | 0.000732 | 4.37 | 2.13 | 2.13 |
| HIST3H2BB | 2.55E-07 | 4.37 | 2.11 | 2.11 |
| RECQL4 | 2.31E-06 | 4.36 | 2.13 | 2.13 |
| HIST1H1E | 2.47E-05 | 4.36 | 2.13 | 2.13 |
| PLK1 | 0.000176 | 4.36 | 2.13 | 2.13 |
| LOC729983 | 0.00017 | 4.36 | 2.12 | 2.12 |
| HIST1H2BC | 1.21E-07 | 4.36 | 2.12 | 2.12 |
| RAB35 | 0.00561 | 4.33 | 2.11 | 2.11 |
| GPX7 | 0.005 | 4.33 | 2.11 | 2.11 |
| SLC39A4 | 0.000333 | 4.33 | 2.11 | 2.11 |
| CDCA4 | 0.000122 | 4.31 | 2.11 | 2.11 |
| C9orf40 | 1.03E-03 | 4.30 | 2.08 | 2.08 |
| HIST1H1C | 0.00122 | 4.29 | 2.10 | 2.10 |
| MGC10981 | 0.00762 | 4.29 | 2.10 | 2.10 |
| DLGAP5 | 0.000725 | 4.28 | 2.10 | 2.10 |
| HOMER3 | 8.65E-05 | 4.28 | 2.10 | 2.10 |
| C13orf27 | 0.000356 | 4.28 | 2.10 | 2.10 |
| POLQ | 0.00144 | 4.28 | 2.10 | 2.10 |
| UQCRH | 2.08E-04 | 4.27 | 2.09 | 2.09 |
| C1orf51 | 3.85E-05 | 4.26 | 2.09 | 2.09 |
| LAPTM4B | 3.50E-05 | 4.25 | 2.08 | 2.08 |
| WARS | 0.00476 | 4.25 | 2.09 | 2.09 |
| GMPS | 5.15E-07 | 4.25 | 2.09 | 2.09 |
| HELLS | 0.000175 | 4.24 | 2.08 | 2.08 |
| RFC4 | 0.000149 | 4.24 | 2.08 | 2.08 |
| PSMD14 | 5.24E-06 | 4.24 | 2.08 | 2.08 |
| IMPA2 | 0.00527 | 4.24 | 2.08 | 2.08 |
| TIMM8A | 2.57E-05 | 4.23 | 2.08 | 2.08 |
| VCAN | 0.00939 | 4.23 | 2.08 | 2.08 |
| MRPL11 | 0.00969 | 4.22 | 2.08 | 2.08 |
| HSPA13 | 0.000177 | 4.21 | 2.07 | 2.07 |
| ARHGDIA | 0.000204 | 4.21 | 2.07 | 2.07 |
| MARK1 | 0.00685 | 4.21 | 2.07 | 2.07 |
| TMEM132A | 4.14E-05 | 4.21 | 2.07 | 2.07 |
| LGALS1 | 0.00118 | 4.21 | 2.07 | 2.07 |
| PSAT1 | 0.000887 | 4.20 | 2.07 | 2.07 |
| PIR | 0.000464 | 4.20 | 2.07 | 2.07 |
| ASAP1 | 0.001473333 | 4.19 | 2.06 | 2.06 |
| CEP76 | 0.000596 | 4.18 | 2.07 | 2.07 |
| PYCR1 | 0.000215 | 4.18 | 2.06 | 2.06 |
| SRGAP2 | 0.00131 | 4.17 | 2.06 | 2.06 |
| IQCG | 0.00251 | 4.16 | 2.06 | 2.06 |
| C12orf48 | 1.11E-04 | 4.15 | 2.01 | 2.01 |
| TPI1 | 1.24E-05 | 4.14 | 2.05 | 2.05 |
| IL20RB | 0.00881 | 4.12 | 2.04 | 2.04 |
| NP | 1.23E-05 | 4.11 | 2.04 | 2.04 |
| LOC646993 | 3.05E-05 | 4.10 | 2.03 | 2.03 |
| HIST1H4C | 0.000288 | 4.08 | 2.03 | 2.03 |
| ADFP | 0.00289 | 4.08 | 2.03 | 2.03 |
| ALDH1L2 | 0.00393 | 4.07 | 2.03 | 2.03 |
| ATP6V1C2 | 0.00756 | 4.07 | 2.03 | 2.03 |
| MRPS17 | 0.000377 | 4.06 | 2.02 | 2.02 |
| ANP32E | 5.89E-04 | 4.05 | 2.02 | 2.02 |
| MCM6 | 3.18E-05 | 4.05 | 2.02 | 2.02 |
| LTBP1 | 0.000335 | 4.04 | 2.01 | 2.01 |
| SUV39H2 | 0.0008578 | 4.04 | 1.99 | 1.99 |
| ICAM1 | 1.01E-03 | 4.04 | 2.01 | 2.01 |
| B3GNT5 | 0.000152 | 4.03 | 2.01 | 2.01 |
| PKMYT1 | 0.000245 | 4.02 | 2.01 | 2.01 |
| H2AFZ | 1.12E-06 | 4.01 | 1.99 | 1.99 |
| GEN1 | 8.67E-05 | 4.00 | 2.00 | 2.00 |
| UBE2S | 0.000114 | 3.97 | 1.99 | 1.99 |
| TMEM70 | 2.18E-05 | 3.97 | 1.99 | 1.99 |
| FEN1 | 3.44E-05 | 3.97 | 1.99 | 1.99 |
| PRDX1 | 0.000315 | 3.96 | 1.98 | 1.98 |
| NDRG1 | 0.00164 | 3.96 | 1.98 | 1.98 |
| TIMELESS | 1.93E-04 | 3.94 | 1.96 | 1.96 |
| YWHAG | 0.000335 | 3.94 | 1.98 | 1.98 |
| PSIP1 | 0.000305 | 3.94 | 1.98 | 1.98 |
| SEC61G | 0.000589 | 3.92 | 1.97 | 1.97 |
| HIST1H4L | 0.000209 | 3.91 | 1.97 | 1.97 |
| VMA21 | 1.44E-06 | 3.91 | 1.97 | 1.97 |
| VEGFA | 0.001273818 | 3.91 | 1.97 | 1.97 |
| CTPS | 8.54E-06 | 3.90 | 1.96 | 1.96 |
| CD163 | 0.00738 | 3.90 | 1.96 | 1.96 |
| MTCH2 | 5.93E-05 | 3.90 | 1.96 | 1.96 |
| HMOX1 | 0.0008962 | 3.89 | 1.96 | 1.96 |
| PPIL1 | 0.00253 | 3.89 | 1.96 | 1.96 |
| ANAPC11 | 0.000147 | 3.89 | 1.96 | 1.96 |
| FAM108C1 | 0.00434 | 3.89 | 1.96 | 1.96 |
| HIST3H2A | 3.22E-07 | 3.88 | 1.96 | 1.96 |
| PIGX | 0.000771 | 3.88 | 1.96 | 1.96 |
| DEK | 0.00369 | 3.88 | 1.95 | 1.95 |
| LAGE3 | 9.92E-06 | 3.87 | 1.95 | 1.95 |
| TP53INP2 | 0.00247 | 3.87 | 1.95 | 1.95 |
| LOC441795 | 2.61E-06 | 3.87 | 1.95 | 1.95 |
| LOC728198 | 2.54E-05 | 3.86 | 1.95 | 1.95 |
| MAD2L1 | 1.24E-05 | 3.86 | 1.95 | 1.95 |
| GTPBP4 | 3.55E-06 | 3.85 | 1.94 | 1.94 |
| FGFR3 | 0.00682 | 3.85 | 1.94 | 1.94 |
| SAC3D1 | 5.68E-05 | 3.84 | 1.94 | 1.94 |
| ZWILCH | 0.0021 | 3.84 | 1.94 | 1.94 |
| NDUFA6 | 9.83E-05 | 3.84 | 1.94 | 1.94 |
| E2F3 | 3.89E-05 | 3.84 | 1.94 | 1.94 |
| DIAPH3 | 2.21E-03 | 3.83 | 1.94 | 1.94 |
| TMSB15B | 0.0019 | 3.82 | 1.93 | 1.93 |
| C6orf167 | 0.000922 | 3.82 | 1.93 | 1.93 |
| CCT5 | 0.00018 | 3.81 | 1.93 | 1.93 |
| PIM1 | 0.00121 | 3.81 | 1.93 | 1.93 |
| COQ2 | 0.00634 | 3.80 | 1.93 | 1.93 |
| NCAPG2 | 8.90E-05 | 3.79 | 1.92 | 1.92 |
| BCL2A1 | 0.00256 | 3.79 | 1.92 | 1.92 |
| HIST1H4F | 4.11E-05 | 3.78 | 1.92 | 1.92 |
| LOC441666 | 0.0048 | 3.78 | 1.92 | 1.92 |
| TOMM22 | 2.69E-06 | 3.77 | 1.92 | 1.92 |
| OPN3 | 0.00955 | 3.76 | 1.91 | 1.91 |
| C1orf59 | 1.95E-05 | 3.76 | 1.91 | 1.91 |
| WDHD1 | 1.76E-05 | 3.76 | 1.91 | 1.91 |
| TMEM123 | 0.000782 | 3.76 | 1.91 | 1.91 |
| LDHA | 5.36E-05 | 3.76 | 1.91 | 1.91 |
| TNFRSF21 | 0.00636 | 3.76 | 1.91 | 1.91 |
| PRICKLE1 | 0.00203 | 3.75 | 1.91 | 1.91 |
| RNF168 | 0.00462 | 3.75 | 1.91 | 1.91 |
| CXCR4 | 5.94E-03 | 3.75 | 1.91 | 1.91 |
| CHML | 4.36E-03 | 3.74 | 1.88 | 1.88 |
| SDC1 | 0.00453 | 3.74 | 1.90 | 1.90 |
| CYTSB | 0.00209 | 3.74 | 1.90 | 1.90 |
| CYHR1 | 0.00108 | 3.73 | 1.90 | 1.90 |
| CCDC109B | 0.000167 | 3.73 | 1.90 | 1.90 |
| INHBA | 0.00926 | 3.73 | 1.90 | 1.90 |
| STIP1 | 5.17E-05 | 3.72 | 1.90 | 1.90 |
| PGM2 | 0.000971 | 3.72 | 1.89 | 1.89 |
| RANBP1 | 8.16E-07 | 3.71 | 1.89 | 1.89 |
| ENSA | 5.41E-06 | 3.71 | 1.89 | 1.89 |
| TFRC | 3.26E-03 | 3.71 | 1.89 | 1.89 |
| EIF2C2 | 0.000124 | 3.70 | 1.89 | 1.89 |
| NUP210 | 0.0091 | 3.70 | 1.89 | 1.89 |
| CDC25A | 0.000315 | 3.70 | 1.89 | 1.89 |
| C8orf33 | 0.000588 | 3.70 | 1.89 | 1.89 |
| MRPL15 | 0.000259 | 3.69 | 1.88 | 1.88 |
| TALDO1 | 0.000203 | 3.69 | 1.88 | 1.88 |
| tcag7.1015 | 2.08E-05 | 3.69 | 1.88 | 1.88 |
| MEX3A | 3.05E-03 | 3.67 | 1.87 | 1.87 |
| SHMT2 | 0.00051785 | 3.67 | 1.88 | 1.88 |
| TTC13 | 0.000392 | 3.66 | 1.87 | 1.87 |
| PSMA7 | 8.50E-05 | 3.66 | 1.87 | 1.87 |
| PPIL5 | 0.000703 | 3.66 | 1.87 | 1.87 |
| STRA13 | 0.00174 | 3.66 | 1.87 | 1.87 |
| SCARB1 | 6.62E-03 | 3.66 | 1.85 | 1.85 |
| GPR126 | 0.002683 | 3.66 | 1.87 | 1.87 |
| PSMB2 | 4.10E-06 | 3.66 | 1.87 | 1.87 |
| OLFML2B | 6.07E-03 | 3.65 | 1.87 | 1.87 |
| CNIH4 | 1.80E-05 | 3.64 | 1.86 | 1.86 |
| TCEB1P3 | 1.14E-05 | 3.64 | 1.86 | 1.86 |
| IFI6 | 0.00369 | 3.63 | 1.86 | 1.86 |
| TPMT | 0.000337 | 3.63 | 1.86 | 1.86 |
| GLRX | 0.000257 | 3.63 | 1.86 | 1.86 |
| TIPIN | 0.000235 | 3.63 | 1.86 | 1.86 |
| MRPL37 | 0.000246 | 3.62 | 1.85 | 1.85 |
| HIST1H3C | 4.33E-05 | 3.61 | 1.85 | 1.85 |
| ATP11B | 0.000163 | 3.61 | 1.85 | 1.85 |
| GINS2 | 0.00241 | 3.61 | 1.85 | 1.85 |
| C19orf53 | 1.26E-07 | 3.61 | 1.85 | 1.85 |
| NNT | 0.00335 | 3.60 | 1.85 | 1.85 |
| KIAA1804 | 0.00916 | 3.59 | 1.85 | 1.85 |
| MTHFD2 | 4.34E-06 | 3.59 | 1.84 | 1.84 |
| C16orf59 | 0.000376 | 3.59 | 1.84 | 1.84 |
| PLK4 | 0.00107 | 3.58 | 1.84 | 1.84 |
| FAM152A | 0.0019705 | 3.58 | 1.83 | 1.83 |
| MASTL | 1.00E-03 | 3.58 | 1.84 | 1.84 |
| WHSC1 | 4.79E-05 | 3.57 | 1.84 | 1.84 |
| FANCE | 0.00154 | 3.57 | 1.84 | 1.84 |
| ENAH | 0.000481 | 3.57 | 1.83 | 1.83 |
| SPAG5 | 0.000175 | 3.56 | 1.83 | 1.83 |
| FBXO31 | 0.0031 | 3.56 | 1.83 | 1.83 |
| ASS1 | 0.000304 | 3.56 | 1.83 | 1.83 |
| RAD21 | 0.000877 | 3.56 | 1.83 | 1.83 |
| PPIF | 0.00105 | 3.56 | 1.83 | 1.83 |
| RGS16 | 0.00944 | 3.55 | 1.83 | 1.83 |
| BOLA2B | 3.22E-05 | 3.55 | 1.83 | 1.83 |
| PDXDC1 | 1.09E-05 | 3.55 | 1.83 | 1.83 |
| TMEM65 | 0.000201 | 3.55 | 1.83 | 1.83 |
| CPVL | 0.00998 | 3.53 | 1.82 | 1.82 |
| SLMO1 | 0.000638 | 3.53 | 1.82 | 1.82 |
| FOXK2 | 0.000101 | 3.53 | 1.82 | 1.82 |
| PFN2 | 0.000188 | 3.53 | 1.82 | 1.82 |
| ADCY7 | 0.000389 | 3.52 | 1.82 | 1.82 |
| LBR | 0.000566 | 3.51 | 1.81 | 1.81 |
| DNAJB11 | 0.000121 | 3.50 | 1.81 | 1.81 |
| RMI1 | 1.27E-05 | 3.49 | 1.80 | 1.80 |
| BST2 | 0.00258 | 3.49 | 1.80 | 1.80 |
| CKS1B | 1.66E-05 | 3.48 | 1.79 | 1.79 |
| SEPT11 | 0.000211 | 3.47 | 1.80 | 1.80 |
| SOCS3 | 0.00106 | 3.47 | 1.79 | 1.79 |
| ILF2 | 0.000266 | 3.47 | 1.79 | 1.79 |
| RP11-129H15.2 | 8.90E-07 | 3.46 | 1.79 | 1.79 |
| HIST1H4H | 0.000165 | 3.46 | 1.79 | 1.79 |
| RABIF | 0.000382 | 3.45 | 1.79 | 1.79 |
| RELT | 0.00105 | 3.45 | 1.79 | 1.79 |
| STAT1 | 0.000788364 | 3.45 | 1.79 | 1.79 |
| POP1 | 0.00797 | 3.45 | 1.79 | 1.79 |
| TCEB1 | 1.23E-03 | 3.45 | 1.78 | 1.78 |
| CTSL1 | 0.0018 | 3.44 | 1.78 | 1.78 |
| TIMP2 | 0.00415 | 3.44 | 1.78 | 1.78 |
| CHAC2 | 0.00353 | 3.44 | 1.78 | 1.78 |
| TTLL4 | 0.0034 | 3.44 | 1.78 | 1.78 |
| MCM3 | 4.81E-06 | 3.43 | 1.78 | 1.78 |
| PPFIA4 | 0.00425 | 3.43 | 1.78 | 1.78 |
| IDI1 | 0.00243 | 3.43 | 1.78 | 1.78 |
| LRRCC1 | 2.31E-04 | 3.42 | 1.77 | 1.77 |
| UFD1L | 1.03E-05 | 3.42 | 1.77 | 1.77 |
| DYRK2 | 3.56E-03 | 3.41 | 1.76 | 1.76 |
| TMEM38B | 1.14E-03 | 3.41 | 1.76 | 1.76 |
| TUBA1C | 2.12E-08 | 3.41 | 1.76 | 1.76 |
| YEATS2 | 3.37E-05 | 3.41 | 1.77 | 1.77 |
| TUBA4A | 1.03E-07 | 3.41 | 1.77 | 1.77 |
| PARP1 | 1.27E-04 | 3.40 | 1.77 | 1.77 |
| SMC6 | 7.65E-05 | 3.40 | 1.76 | 1.76 |
| GNB4 | 2.83E-04 | 3.39 | 1.76 | 1.76 |
| SQLE | 0.00825 | 3.39 | 1.76 | 1.76 |
| LOC203510 | 3.91E-05 | 3.39 | 1.76 | 1.76 |
| ENY2 | 0.000208 | 3.39 | 1.76 | 1.76 |
| TOMM5 | 3.10E-05 | 3.38 | 1.76 | 1.76 |
| MAL2 | 0.00173 | 3.38 | 1.76 | 1.76 |
| BAIAP2L1 | 0.000399 | 3.38 | 1.76 | 1.76 |
| RGS10 | 0.00241 | 3.37 | 1.75 | 1.75 |
| TDP1 | 5.87E-05 | 3.37 | 1.75 | 1.75 |
| PDCD5 | 0.000196 | 3.37 | 1.75 | 1.75 |
| CKAP4 | 0.00364 | 3.37 | 1.75 | 1.75 |
| SLC35F2 | 0.00621 | 3.37 | 1.75 | 1.75 |
| TOR3A | 1.03E-05 | 3.37 | 1.75 | 1.75 |
| DPH3 | 0.000182 | 3.37 | 1.75 | 1.75 |
| HIST1H2AK | 1.25E-07 | 3.36 | 1.75 | 1.75 |
| LOC389842 | 7.70E-05 | 3.36 | 1.75 | 1.75 |
| NUDT1 | 0.000121 | 3.35 | 1.74 | 1.74 |
| SLC6A9 | 0.00749 | 3.35 | 1.74 | 1.74 |
| PDXK | 2.01E-03 | 3.35 | 1.74 | 1.74 |
| HIST1H2AH | 1.63E-07 | 3.35 | 1.74 | 1.74 |
| DDX39 | 1.50E-05 | 3.35 | 1.74 | 1.74 |
| ZUFSP | 0.00016 | 3.34 | 1.74 | 1.74 |
| PCNA | 4.48E-05 | 3.34 | 1.74 | 1.74 |
| FAM83H | 0.000419 | 3.33 | 1.74 | 1.74 |
| DERL1 | 0.000332 | 3.33 | 1.74 | 1.74 |
| TMSB10 | 2.60E-07 | 3.33 | 1.74 | 1.74 |
| YARS | 0.000435 | 3.33 | 1.74 | 1.74 |
| TMEFF1 | 0.00265 | 3.32 | 1.73 | 1.73 |
| PPP1R16A | 1.15E-05 | 3.31 | 1.73 | 1.73 |
| AMMECR1 | 0.00316 | 3.31 | 1.73 | 1.73 |
| MSH2 | 0.0002905 | 3.31 | 1.72 | 1.72 |
| TCF19 | 7.67E-05 | 3.30 | 1.72 | 1.72 |
| MTFR1 | 0.00233 | 3.30 | 1.72 | 1.72 |
| DPH2 | 0.00621 | 3.30 | 1.72 | 1.72 |
| C6orf115 | 5.18E-06 | 3.30 | 1.72 | 1.72 |
| ISG20 | 0.00455 | 3.29 | 1.72 | 1.72 |
| INTS7 | 0.00566 | 3.29 | 1.72 | 1.72 |
| SAMHD1 | 0.00852 | 3.28 | 1.72 | 1.72 |
| NDUFA4 | 0.0009925 | 3.28 | 1.71 | 1.71 |
| C1orf122 | 1.89E-05 | 3.28 | 1.71 | 1.71 |
| FOXRED2 | 0.00849 | 3.28 | 1.71 | 1.71 |
| KPNA2 | 5.44E-07 | 3.28 | 1.71 | 1.71 |
| TMEM106C | 0.000339 | 3.28 | 1.71 | 1.71 |
| C15orf23 | 7.78E-06 | 3.28 | 1.71 | 1.71 |
| HTATIP2 | 1.58E-03 | 3.27 | 1.71 | 1.71 |
| BARD1 | 4.65E-05 | 3.27 | 1.71 | 1.71 |
| PIK3R3 | 0.002 | 3.27 | 1.71 | 1.71 |
| NCAPD2 | 0.00134 | 3.27 | 1.71 | 1.71 |
| DHFR | 9.24E-06 | 3.27 | 1.71 | 1.71 |
| NDUFAB1 | 0.000139 | 3.26 | 1.71 | 1.71 |
| PTPLB | 0.00406 | 3.26 | 1.70 | 1.70 |
| GART | 7.80E-05 | 3.26 | 1.70 | 1.70 |
| PPM1G | 0.000382 | 3.25 | 1.70 | 1.70 |
| LIN9 | 5.41E-04 | 3.25 | 1.70 | 1.70 |
| HSPA14 | 8.54E-06 | 3.25 | 1.70 | 1.70 |
| RHOBTB1 | 0.00312 | 3.25 | 1.70 | 1.70 |
| NOXO1 | 0.00188 | 3.25 | 1.70 | 1.70 |
| ASF1B | 8.74E-09 | 3.25 | 1.70 | 1.70 |
| APOLD1 | 0.00379 | 3.24 | 1.70 | 1.70 |
| KIAA0746 | 0.000777 | 3.24 | 1.70 | 1.70 |
| MRPS25 | 0.000123 | 3.24 | 1.70 | 1.70 |
| PSMA5 | 8.16E-05 | 3.24 | 1.70 | 1.70 |
| C5orf13 | 2.53E-03 | 3.24 | 1.70 | 1.70 |
| ACTL6A | 0.000222 | 3.24 | 1.69 | 1.69 |
| TPM3 | 2.62E-06 | 3.24 | 1.69 | 1.69 |
| KLHL23 | 0.00163 | 3.24 | 1.69 | 1.69 |
| COX8A | 4.81E-05 | 3.23 | 1.69 | 1.69 |
| HIST2H4B | 0.000152 | 3.22 | 1.69 | 1.69 |
| SLC30A7 | 3.43E-05 | 3.22 | 1.69 | 1.69 |
| P4HB | 0.000199 | 3.22 | 1.69 | 1.69 |
| CCNB2 | 9.01E-05 | 3.21 | 1.68 | 1.68 |
| KIAA0513 | 0.00257 | 3.21 | 1.68 | 1.68 |
| CARHSP1 | 0.000386 | 3.21 | 1.68 | 1.68 |
| WSB2 | 0.00013 | 3.21 | 1.68 | 1.68 |
| PGK1 | 5.38E-06 | 3.21 | 1.68 | 1.68 |
| ARPC4 | 0.00749 | 3.21 | 1.68 | 1.68 |
| RPA3 | 0.000408 | 3.21 | 1.68 | 1.68 |
| FLJ20674 | 0.00652 | 3.20 | 1.68 | 1.68 |
| TBC1D7 | 6.77E-05 | 3.20 | 1.68 | 1.68 |
| EIF4E2 | 0.000423 | 3.19 | 1.67 | 1.67 |
| FAM96B | 0.000264 | 3.19 | 1.67 | 1.67 |
| CALU | 0.000294 | 3.19 | 1.67 | 1.67 |
| SAR1A | 2.98E-05 | 3.18 | 1.67 | 1.67 |
| GK | 0.00106 | 3.18 | 1.67 | 1.67 |
| GUK1 | 7.53E-06 | 3.18 | 1.67 | 1.67 |
| PTK2 | 0.000117 | 3.18 | 1.67 | 1.67 |
| SLC25A5 | 4.60E-07 | 3.18 | 1.67 | 1.67 |
| SENP5 | 5.51E-05 | 3.18 | 1.67 | 1.67 |
| VBP1 | 0.000369 | 3.18 | 1.67 | 1.67 |
| LMNB2 | 6.47E-05 | 3.17 | 1.66 | 1.66 |
| MPHOSPH9 | 3.58E-07 | 3.17 | 1.66 | 1.66 |
| FANCD2 | 5.06E-05 | 3.17 | 1.66 | 1.66 |
| BOP1 | 0.00023 | 3.17 | 1.66 | 1.66 |
| PAICS | 1.69E-05 | 3.17 | 1.66 | 1.66 |
| BOLA3 | 1.09E-04 | 3.17 | 1.66 | 1.66 |
| GLT25D1 | 0.000359 | 3.17 | 1.66 | 1.66 |
| HIST1H4I | 0.000144 | 3.16 | 1.66 | 1.66 |
| SSR3 | 0.00301 | 3.16 | 1.66 | 1.66 |
| COX4NB | 0.000465 | 3.16 | 1.66 | 1.66 |
| ARL6IP1 | 2.00E-04 | 3.16 | 1.66 | 1.66 |
| ZNF707 | 0.00597 | 3.16 | 1.66 | 1.66 |
| SOX12 | 0.00952 | 3.16 | 1.66 | 1.66 |
| TUBB | 1.88E-05 | 3.16 | 1.66 | 1.66 |
| SNRPD1 | 6.28E-05 | 3.16 | 1.66 | 1.66 |
| DTYMK | 0.000443 | 3.16 | 1.66 | 1.66 |
| ASCC1 | 0.00043 | 3.16 | 1.66 | 1.66 |
| ATP6AP2 | 1.31E-06 | 3.15 | 1.65 | 1.65 |
| SPARC | 0.00761 | 3.15 | 1.65 | 1.65 |
| CFL1 | 1.10E-07 | 3.15 | 1.65 | 1.65 |
| KIAA1609 | 0.00529 | 3.15 | 1.65 | 1.65 |
| PSMA4 | 0.00019 | 3.14 | 1.65 | 1.65 |
| NUDT21 | 4.61E-05 | 3.14 | 1.65 | 1.65 |
| AK2 | 8.97E-05 | 3.14 | 1.65 | 1.65 |
| MYD88 | 0.000219 | 3.14 | 1.65 | 1.65 |
| FXYD5 | 6.03E-05 | 3.13 | 1.65 | 1.65 |
| TMEM183B | 6.67E-05 | 3.13 | 1.65 | 1.65 |
| YBX1 | 0.00125 | 3.13 | 1.65 | 1.65 |
| NDUFB9 | 0.002145 | 3.13 | 1.65 | 1.65 |
| RBM8A | 5.77E-06 | 3.13 | 1.64 | 1.64 |
| PRKRIR | 0.000254 | 3.13 | 1.64 | 1.64 |
| SF3B14 | 0.000287 | 3.13 | 1.64 | 1.64 |
| LOC100128266 | 5.08E-06 | 3.12 | 1.64 | 1.64 |
| KIF21A | 0.00385 | 3.12 | 1.64 | 1.64 |
| AP2S1 | 0.00842 | 3.12 | 1.64 | 1.64 |
| COX6A1 | 0.00034 | 3.11 | 1.64 | 1.64 |
| TIMM17A | 4.07E-06 | 3.11 | 1.64 | 1.64 |
| ATP13A3 | 8.01E-05 | 3.11 | 1.64 | 1.64 |
| FAM123B | 0.000174 | 3.10 | 1.63 | 1.63 |
| LPCAT1 | 0.000528 | 3.10 | 1.63 | 1.63 |
| ADAM8 | 0.00117 | 3.10 | 1.63 | 1.63 |
| HSPB11 | 0.000391 | 3.10 | 1.63 | 1.63 |
| TBL1XR1 | 7.26E-05 | 3.09 | 1.63 | 1.63 |
| SLC2A4RG | 3.74E-05 | 3.09 | 1.63 | 1.63 |
| DOLPP1 | 0.00108 | 3.09 | 1.63 | 1.63 |
| CENPP | 4.37E-05 | 3.08 | 1.63 | 1.63 |
| NUS1 | 8.05E-05 | 3.08 | 1.62 | 1.62 |
| SAE1 | 0.000127 | 3.08 | 1.62 | 1.62 |
| PFDN6 | 6.58E-05 | 3.08 | 1.62 | 1.62 |
| YWHAZ | 3.27E-05 | 3.08 | 1.62 | 1.62 |
| DONSON | 0.00035 | 3.08 | 1.62 | 1.62 |
| CYCS | 3.92E-06 | 3.08 | 1.62 | 1.62 |
| GLT8D3 | 0.00231 | 3.07 | 1.62 | 1.62 |
| FAM49B | 1.40E-05 | 3.07 | 1.62 | 1.62 |
| PRIM2 | 4.06E-05 | 3.07 | 1.62 | 1.62 |
| STARD4 | 0.00875 | 3.07 | 1.62 | 1.62 |
| CHCHD2 | 0.004736667 | 3.06 | 1.61 | 1.61 |
| FUCA2 | 3.88E-05 | 3.06 | 1.61 | 1.61 |
| RAN | 5.41E-07 | 3.06 | 1.61 | 1.61 |
| TIMM10 | 0.0098 | 3.06 | 1.61 | 1.61 |
| APOL6 | 0.00688 | 3.06 | 1.61 | 1.61 |
| PRKDC | 0.00144 | 3.06 | 1.61 | 1.61 |
| RNASEH2A | 5.29E-06 | 3.06 | 1.61 | 1.61 |
| TMEM97 | 0.00142 | 3.06 | 1.61 | 1.61 |
| CACYBP | 6.11E-06 | 3.05 | 1.61 | 1.61 |
| PSMD8 | 7.24E-05 | 3.05 | 1.61 | 1.61 |
| FTL | 0.00191 | 3.05 | 1.61 | 1.61 |
| LIMK1 | 1.35E-05 | 3.05 | 1.61 | 1.61 |
| HSPD1 | 1.33E-05 | 3.04 | 1.61 | 1.61 |
| CEBPG | 0.000816 | 3.04 | 1.61 | 1.61 |
| NCOA7 | 0.0088 | 3.04 | 1.60 | 1.60 |
| TUBB4 | 7.49E-07 | 3.04 | 1.60 | 1.60 |
| KDELR2 | 1.32E-04 | 3.04 | 1.60 | 1.60 |
| MSN | 0.000734 | 3.04 | 1.60 | 1.60 |
| RIOK3 | 0.00597 | 3.03 | 1.60 | 1.60 |
| EPRS | 5.12E-05 | 3.03 | 1.60 | 1.60 |
| TIMM23 | 7.13E-05 | 3.03 | 1.60 | 1.60 |
| BPNT1 | 4.18E-05 | 3.03 | 1.60 | 1.60 |
| TUBB2A | 5.38E-07 | 3.03 | 1.60 | 1.60 |
| C13orf37 | 0.00393 | 3.03 | 1.60 | 1.60 |
| TUBB6 | 1.20E-07 | 3.02 | 1.60 | 1.60 |
| HPRT1 | 3.73E-05 | 3.02 | 1.60 | 1.60 |
| DCUN1D5 | 0.000537 | 3.02 | 1.60 | 1.60 |
| MED30 | 0.00432 | 3.02 | 1.60 | 1.60 |
| KTI12 | 6.75E-05 | 3.02 | 1.60 | 1.60 |
| RBX1 | 0.000393 | 3.02 | 1.59 | 1.59 |
| PUF60 | 0.000274 | 3.01 | 1.59 | 1.59 |
| MGST3 | 8.16E-05 | 3.01 | 1.59 | 1.59 |
| PPP1R14B | 0.000443 | 3.01 | 1.59 | 1.59 |
| MRPL19 | 7.77E-05 | 3.01 | 1.59 | 1.59 |
| CCDC44 | 0.00144 | 3.01 | 1.59 | 1.59 |
| NUDT19 | 0.00478 | 3.01 | 1.59 | 1.59 |
| POLR2I | 0.000388 | 3.01 | 1.59 | 1.59 |
| SHFM1 | 0.000417 | 3.01 | 1.59 | 1.59 |
| COX5A | 0.000896 | 3.00 | 1.59 | 1.59 |
| CKAP5 | 1.77E-05 | 3.00 | 1.59 | 1.59 |
| GOLT1B | 0.00023 | 3.00 | 1.59 | 1.59 |
| NSFL1C | 0.000406 | 3.00 | 1.59 | 1.59 |
| MRPL44 | 0.000924 | 3.00 | 1.59 | 1.59 |
| RP13-401N8.2 | 2.64E-07 | 0.33 | -1.59 | 1.59 |
| FBXL20 | 0.000294 | 0.33 | -1.59 | 1.59 |
| ARHGEF12 | 0.000498 | 0.33 | -1.59 | 1.59 |
| SLC25A26 | 2.33E-05 | 0.33 | -1.59 | 1.59 |
| ALS2CR4 | 0.000463 | 0.33 | -1.60 | 1.60 |
| ETV1 | 0.00236 | 0.33 | -1.60 | 1.60 |
| tcag7.1177 | 5.31E-05 | 0.33 | -1.60 | 1.60 |
| NANOG | 0.00323 | 0.33 | -1.61 | 1.61 |
| GPRASP2 | 9.56E-08 | 0.33 | -1.61 | 1.61 |
| RNF128 | 0.000939 | 0.33 | -1.61 | 1.61 |
| GRIK3 | 0.00932 | 0.33 | -1.62 | 1.62 |
| KCND3 | 7.64E-05 | 0.32 | -1.62 | 1.62 |
| ZSCAN18 | 5.05E-05 | 0.32 | -1.62 | 1.62 |
| PNPLA7 | 0.00359 | 0.32 | -1.63 | 1.63 |
| SLC25A30 | 0.00678 | 0.32 | -1.63 | 1.63 |
| KCNJ8 | 0.00089 | 0.32 | -1.63 | 1.63 |
| KIT | 1.77E-05 | 0.32 | -1.63 | 1.63 |
| C3orf18 | 0.000652 | 0.32 | -1.63 | 1.63 |
| ZMAT1 | 1.29E-07 | 0.32 | -1.64 | 1.64 |
| PLAT | 7.48E-06 | 0.32 | -1.64 | 1.64 |
| ZC3H6 | 7.51E-09 | 0.32 | -1.65 | 1.65 |
| IL11RA | 7.23E-07 | 0.32 | -1.65 | 1.65 |
| PLK2 | 1.16E-06 | 0.32 | -1.65 | 1.65 |
| EDNRB | 0.000369 | 0.32 | -1.65 | 1.65 |
| ZNF154 | 1.01E-06 | 0.32 | -1.65 | 1.65 |
| EGFLAM | 0.00816 | 0.32 | -1.65 | 1.65 |
| SPEF2 | 3.71E-05 | 0.32 | -1.65 | 1.65 |
| TRIM9 | 0.00153 | 0.32 | -1.65 | 1.65 |
| C21orf34 | 2.11E-08 | 0.32 | -1.66 | 1.66 |
| GSTM1 | 1.18E-06 | 0.32 | -1.66 | 1.66 |
| NAV2 | 3.37E-06 | 0.32 | -1.66 | 1.66 |
| EYS | 7.05E-05 | 0.31 | -1.67 | 1.67 |
| TRO | 0.000151 | 0.31 | -1.67 | 1.67 |
| C15orf40 | 0.00177 | 0.31 | -1.67 | 1.67 |
| CHIC1 | 0.000884 | 0.31 | -1.67 | 1.67 |
| MGP | 1.67E-06 | 0.31 | -1.68 | 1.68 |
| NCRNA00153 | 0.00129 | 0.31 | -1.68 | 1.68 |
| RBM43 | 2.03E-03 | 0.31 | -1.68 | 1.68 |
| CRYBA1 | 0.000319 | 0.31 | -1.68 | 1.68 |
| MRVI1 | 0.0078 | 0.31 | -1.68 | 1.68 |
| LRP2BP | 1.69E-06 | 0.31 | -1.68 | 1.68 |
| SLC4A7 | 0.00189 | 0.31 | -1.69 | 1.69 |
| RUNX1T1 | 0.00177 | 0.31 | -1.69 | 1.69 |
| ANKRD42 | 3.10E-05 | 0.31 | -1.69 | 1.69 |
| ECHDC2 | 2.43E-06 | 0.31 | -1.69 | 1.69 |
| SRPX | 0.000664 | 0.31 | -1.69 | 1.69 |
| LOC151878 | 6.84E-07 | 0.31 | -1.69 | 1.69 |
| TTC23L | 0.0075 | 0.31 | -1.70 | 1.70 |
| LPCAT2 | 1.37E-06 | 0.31 | -1.70 | 1.70 |
| F2RL2 | 0.000368 | 0.31 | -1.70 | 1.70 |
| LAYN | 0.000117 | 0.31 | -1.71 | 1.71 |
| NAIP | 1.24E-07 | 0.31 | -1.71 | 1.71 |
| AASS | 8.71E-07 | 0.31 | -1.71 | 1.71 |
| CDC14A | 3.93E-06 | 0.31 | -1.71 | 1.71 |
| DMKN | 0.00245 | 0.30 | -1.71 | 1.71 |
| NEDD4L | 5.16E-06 | 0.30 | -1.72 | 1.72 |
| PDE1C | 3.24E-05 | 0.30 | -1.73 | 1.73 |
| PPP1R14A | 2.98E-06 | 0.30 | -1.73 | 1.73 |
| SH3BGRL2 | 1.30E-06 | 0.30 | -1.73 | 1.73 |
| ANKRA2 | 8.12E-10 | 0.30 | -1.73 | 1.73 |
| LRP2 | 0.00549 | 0.30 | -1.73 | 1.73 |
| FMOD | 1.20E-05 | 0.30 | -1.74 | 1.74 |
| SUGT1L1 | 0.000819 | 0.30 | -1.74 | 1.74 |
| MYH3 | 2.27E-05 | 0.30 | -1.74 | 1.74 |
| XKR6 | 0.000312 | 0.30 | -1.74 | 1.74 |
| PDGFRA | 9.28E-06 | 0.29 | -1.77 | 1.77 |
| FANK1 | 7.99E-09 | 0.29 | -1.78 | 1.78 |
| NDN | 2.89E-06 | 0.29 | -1.78 | 1.78 |
| HEMK1 | 3.68E-09 | 0.29 | -1.78 | 1.78 |
| ARHGAP19 | 2.54E-08 | 0.29 | -1.78 | 1.78 |
| FOS | 0.00242 | 0.29 | -1.79 | 1.79 |
| ZNF334 | 0.000426 | 0.29 | -1.80 | 1.80 |
| ABCA5 | 1.44E-09 | 0.29 | -1.80 | 1.80 |
| SERPINA5 | 0.00381 | 0.29 | -1.81 | 1.81 |
| IRS1 | 5.87E-04 | 0.29 | -1.81 | 1.81 |
| LRIG1 | 2.93E-06 | 0.29 | -1.81 | 1.81 |
| RHOJ | 0.000867 | 0.29 | -1.81 | 1.81 |
| PGPEP1 | 9.51E-06 | 0.28 | -1.81 | 1.81 |
| GATA3 | 2.25E-05 | 0.28 | -1.82 | 1.82 |
| CACNA1C | 9.41E-05 | 0.28 | -1.82 | 1.82 |
| PLA2R1 | 8.45E-05 | 0.28 | -1.82 | 1.82 |
| C5orf41 | 5.51E-06 | 0.28 | -1.82 | 1.82 |
| ACADSB | 4.34E-05 | 0.28 | -1.85 | 1.85 |
| GOLSYN | 1.09E-05 | 0.28 | -1.83 | 1.83 |
| IGF2 | 0.00832 | 0.28 | -1.83 | 1.83 |
| FLJ12078 | 0.000167 | 0.28 | -1.84 | 1.84 |
| FLJ27365 | 6.68E-05 | 0.28 | -1.85 | 1.85 |
| SPATA18 | 0.000105 | 0.28 | -1.85 | 1.85 |
| NME5 | 0.00402 | 0.28 | -1.85 | 1.85 |
| COX7A1 | 4.16E-05 | 0.28 | -1.85 | 1.85 |
| KIF13B | 8.68E-07 | 0.28 | -1.86 | 1.86 |
| AGTR1 | 9.62E-06 | 0.28 | -1.86 | 1.86 |
| C11orf61 | 0.000747 | 0.28 | -1.86 | 1.86 |
| ACTA2 | 5.93E-05 | 0.27 | -1.86 | 1.86 |
| GRAMD1C | 2.59E-05 | 0.27 | -1.88 | 1.88 |
| VIPR1 | 0.00255 | 0.27 | -1.88 | 1.88 |
| LOC389634 | 3.55E-05 | 0.27 | -1.89 | 1.89 |
| TTC18 | 1.84E-06 | 0.27 | -1.89 | 1.89 |
| FLJ13197 | 1.11E-05 | 0.27 | -1.89 | 1.89 |
| TPTE | 1.84E-05 | 0.27 | -1.89 | 1.89 |
| BCL2 | 3.97E-07 | 0.27 | -1.90 | 1.90 |
| POTEB | 0.000827 | 0.27 | -1.91 | 1.91 |
| LOC100128979 | 4.00E-09 | 0.27 | -1.91 | 1.91 |
| POTED | 0.00023 | 0.27 | -1.91 | 1.91 |
| AFF3 | 0.00193 | 0.26 | -1.92 | 1.92 |
| ZNF214 | 3.97E-06 | 0.26 | -1.92 | 1.92 |
| DACH1 | 0.000458 | 0.26 | -1.93 | 1.93 |
| LCA5 | 1.40E-06 | 0.26 | -1.93 | 1.93 |
| PDE2A | 2.39E-05 | 0.26 | -1.93 | 1.93 |
| DKK3 | 3.60406E-05 | 0.26 | -1.93 | 1.93 |
| EFNB3 | 0.000247 | 0.26 | -1.93 | 1.93 |
| RERG | 1.51E-07 | 0.26 | -1.94 | 1.94 |
| FMO5 | 0.000198 | 0.26 | -1.94 | 1.94 |
| AZGP1 | 0.00789 | 0.26 | -1.94 | 1.94 |
| MLPH | 4.15E-06 | 0.26 | -1.96 | 1.96 |
| CXCL12 | 9.92E-04 | 0.26 | -1.96 | 1.96 |
| PPP1R3C | 0.000536 | 0.26 | -1.96 | 1.96 |
| BMP5 | 0.00183 | 0.26 | -1.96 | 1.96 |
| LOC399959 | 0.000216 | 0.26 | -1.97 | 1.97 |
| ANKRD20A2 | 2.93E-03 | 0.25 | -1.99 | 1.99 |
| GPR81 | 0.00132 | 0.25 | -1.98 | 1.98 |
| PHF15 | 4.64E-05 | 0.25 | -1.98 | 1.98 |
| TUBB2B | 0.000913 | 0.25 | -1.98 | 1.98 |
| LOC221442 | 2.21E-05 | 0.25 | -1.98 | 1.98 |
| GLI3 | 1.17E-06 | 0.25 | -1.99 | 1.99 |
| AFAP1L2 | 7.45E-07 | 0.25 | -1.99 | 1.99 |
| ARL4A | 3.22E-05 | 0.25 | -2.01 | 2.01 |
| LIFR | 4.67E-06 | 0.25 | -2.02 | 2.02 |
| MPPED2 | 0.00093 | 0.24 | -2.04 | 2.04 |
| SP2 | 6.46E-07 | 0.24 | -2.05 | 2.05 |
| INPP4B | 9.05E-08 | 0.24 | -2.05 | 2.05 |
| ITGBL1 | 3.26E-06 | 0.24 | -2.05 | 2.05 |
| LAMA3 | 1.63E-03 | 0.24 | -2.09 | 2.09 |
| MASP2 | 3.83E-07 | 0.24 | -2.06 | 2.06 |
| KIAA0329 | 2.70E-07 | 0.24 | -2.06 | 2.06 |
| SCN4A | 0.000954 | 0.24 | -2.07 | 2.07 |
| SPAG8 | 0.000645 | 0.24 | -2.07 | 2.07 |
| RHD | 6.99E-07 | 0.24 | -2.08 | 2.08 |
| CXCL2 | 9.25E-05 | 0.24 | -2.08 | 2.08 |
| LOC284112 | 3.58E-07 | 0.24 | -2.08 | 2.08 |
| GRAMD3 | 3.74E-09 | 0.23 | -2.09 | 2.09 |
| C9orf125 | 0.00189 | 0.23 | -2.10 | 2.10 |
| CD200 | 7.76E-06 | 0.23 | -2.12 | 2.12 |
| POTEC | 4.69E-05 | 0.23 | -2.12 | 2.12 |
| SNRPN | 1.18E-06 | 0.23 | -2.17 | 2.17 |
| FAM47E | 6.33E-09 | 0.23 | -2.13 | 2.13 |
| IGF1 | 0.0008576 | 0.23 | -2.15 | 2.15 |
| AMIGO2 | 9.25E-08 | 0.23 | -2.15 | 2.15 |
| ROBO3 | 1.38E-07 | 0.23 | -2.15 | 2.15 |
| MAPT | 7.34E-04 | 0.23 | -2.16 | 2.16 |
| HS3ST4 | 0.000308 | 0.22 | -2.16 | 2.16 |
| AGR3 | 0.00135 | 0.22 | -2.18 | 2.18 |
| CCDC74B | 0.00217 | 0.22 | -2.20 | 2.20 |
| NTF4 | 5.79E-05 | 0.22 | -2.20 | 2.20 |
| LTBP2 | 2.71E-05 | 0.22 | -2.21 | 2.21 |
| LOC100130464 | 0.00204 | 0.22 | -2.21 | 2.21 |
| GPRASP1 | 1.00E-08 | 0.22 | -2.21 | 2.21 |
| CYP4F11 | 7.82E-05 | 0.21 | -2.22 | 2.22 |
| ITM2A | 8.52E-07 | 0.21 | -2.24 | 2.24 |
| SCN4B | 0.000272 | 0.21 | -2.25 | 2.25 |
| N4BP2L1 | 6.84E-07 | 0.21 | -2.25 | 2.25 |
| C9orf61 | 3.15E-06 | 0.21 | -2.28 | 2.28 |
| C14orf132 | 9.17E-07 | 0.21 | -2.27 | 2.27 |
| LOC100128364 | 1.93E-06 | 0.21 | -2.27 | 2.27 |
| TMEM144 | 2.93E-07 | 0.21 | -2.28 | 2.28 |
| CMYA5 | 2.14E-06 | 0.21 | -2.29 | 2.29 |
| KCNMA1 | 2.14E-04 | 0.20 | -2.32 | 2.32 |
| PDZK1 | 2.85E-05 | 0.20 | -2.30 | 2.30 |
| LOC729029 | 0.000178 | 0.20 | -2.31 | 2.31 |
| CBX7 | 1.10E-10 | 0.20 | -2.31 | 2.31 |
| DMRTC1 | 0.000452 | 0.20 | -2.31 | 2.31 |
| CTTNBP2 | 0.000104 | 0.20 | -2.31 | 2.31 |
| C10orf79 | 1.57E-05 | 0.20 | -2.31 | 2.31 |
| SAMD5 | 2.89E-05 | 0.20 | -2.33 | 2.33 |
| ABCG2 | 0.000926 | 0.20 | -2.35 | 2.35 |
| KIF5A | 5.84E-05 | 0.19 | -2.37 | 2.37 |
| NAV3 | 2.43E-05 | 0.19 | -2.38 | 2.38 |
| OR5P2 | 0.00278 | 0.19 | -2.38 | 2.38 |
| TMTC1 | 0.00015965 | 0.19 | -2.41 | 2.41 |
| RBMS3 | 2.50E-06 | 0.19 | -2.45 | 2.45 |
| LOC285944 | 0.000284 | 0.19 | -2.43 | 2.43 |
| STC2 | 2.23E-07 | 0.18 | -2.46 | 2.46 |
| RSPO3 | 0.00309 | 0.18 | -2.45 | 2.45 |
| MAGI1 | 8.73E-08 | 0.18 | -2.45 | 2.45 |
| AK5 | 5.69E-09 | 0.18 | -2.46 | 2.46 |
| SPARCL1 | 1.52E-08 | 0.18 | -2.47 | 2.47 |
| THSD4 | 1.22E-06 | 0.18 | -2.48 | 2.48 |
| MAMDC2 | 1.02E-06 | 0.18 | -2.49 | 2.49 |
| PDGFA | 8.37E-10 | 0.18 | -2.49 | 2.49 |
| IL33 | 2.54E-07 | 0.18 | -2.50 | 2.50 |
| ADAMTS5 | 6.57E-08 | 0.18 | -2.51 | 2.51 |
| NTRK2 | 1.51E-06 | 0.18 | -2.51 | 2.51 |
| EGR3 | 6.98E-06 | 0.17 | -2.52 | 2.52 |
| ANO1 | 8.70E-13 | 0.17 | -2.53 | 2.53 |
| NTN4 | 1.81E-10 | 0.17 | -2.53 | 2.53 |
| MAP2K6 | 6.64E-05 | 0.17 | -2.53 | 2.53 |
| SIGLEC15 | 0.000142 | 0.17 | -2.53 | 2.53 |
| HOXA3 | 1.97E-08 | 0.17 | -2.53 | 2.53 |
| PRKD1 | 8.70E-06 | 0.17 | -2.55 | 2.55 |
| BEX1 | 1.75E-07 | 0.17 | -2.55 | 2.55 |
| MME | 6.92E-07 | 0.17 | -2.56 | 2.56 |
| DNALI1 | 7.66E-08 | 0.17 | -2.58 | 2.58 |
| SEMA3G | 0.000202 | 0.17 | -2.59 | 2.59 |
| DMD | 8.95E-06 | 0.17 | -2.61 | 2.61 |
| SYNPO2 | 4.86E-07 | 0.16 | -2.62 | 2.62 |
| CDO1 | 2.63E-06 | 0.16 | -2.64 | 2.64 |
| SEMA5A | 4.42E-05 | 0.16 | -2.65 | 2.65 |
| GRIA4 | 7.74E-07 | 0.16 | -2.68 | 2.68 |
| OR5P3 | 6.81E-07 | 0.15 | -2.70 | 2.70 |
| DYNLRB2 | 6.81E-08 | 0.15 | -2.71 | 2.71 |
| WIF1 | 0.00175 | 0.15 | -2.71 | 2.71 |
| ODZ2 | 1.37E-05 | 0.15 | -2.71 | 2.71 |
| DST | 1.57E-09 | 0.15 | -2.72 | 2.72 |
| LOC284232 | 7.26E-09 | 0.15 | -2.71 | 2.71 |
| ERBB4 | 5.01E-07 | 0.15 | -2.77 | 2.77 |
| ALDH7A1 | 0.000536 | 0.15 | -2.72 | 2.72 |
| HBB | 2.86E-03 | 0.15 | -2.74 | 2.74 |
| IQCA1 | 2.33E-08 | 0.15 | -2.75 | 2.75 |
| FGF1 | 7.57E-07 | 0.15 | -2.82 | 2.82 |
| SLC27A6 | 1.63E-06 | 0.14 | -2.79 | 2.79 |
| CLDN11 | 4.74E-07 | 0.14 | -2.90 | 2.90 |
| IQUB | 3.54E-05 | 0.14 | -2.80 | 2.80 |
| C4orf18 | 1.15E-10 | 0.14 | -2.81 | 2.81 |
| TSHZ2 | 2.61E-10 | 0.14 | -2.81 | 2.81 |
| RAI2 | 4.25E-08 | 0.14 | -2.82 | 2.82 |
| CNN1 | 1.36E-05 | 0.14 | -2.83 | 2.83 |
| IGJ | 1.56E-06 | 0.14 | -2.83 | 2.83 |
| HOXA7 | 2.52E-05 | 0.14 | -2.83 | 2.83 |
| C5orf4 | 1.40E-06 | 0.14 | -2.84 | 2.84 |
| PTN | 0.000908 | 0.14 | -2.84 | 2.84 |
| CLSTN2 | 1.12E-05 | 0.14 | -2.85 | 2.85 |
| MYH11 | 8.26E-09 | 0.14 | -2.86 | 2.86 |
| ITIH5 | 4.17E-05 | 0.14 | -2.87 | 2.87 |
| IGHA1 | 9.19E-05 | 0.13 | -2.91 | 2.91 |
| PIGR | 1.42E-06 | 0.13 | -2.97 | 2.97 |
| PAR5 | 3.16E-13 | 0.12 | -3.04 | 3.04 |
| HOXA4 | 2.40E-12 | 0.12 | -3.04 | 3.04 |
| LOC442245 | 3.01E-10 | 0.12 | -3.10 | 3.10 |
| DCDC2 | 1.02E-05 | 0.11 | -3.14 | 3.14 |
| IL20RA | 3.32E-08 | 0.11 | -3.16 | 3.16 |
| CX3CR1 | 6.96E-10 | 0.11 | -3.16 | 3.16 |
| LOC283174 | 1.77E-07 | 0.11 | -3.16 | 3.16 |
| ESR1 | 3.22E-07 | 0.11 | -3.16 | 3.16 |
| NRG1 | 1.21E-05 | 0.11 | -3.19 | 3.19 |
| APOD | 5.38E-05 | 0.11 | -3.20 | 3.20 |
| CAPN8 | 3.44E-06 | 0.11 | -3.25 | 3.25 |
| KCNJ16 | 3.63E-06 | 0.10 | -3.27 | 3.27 |
| KLHL13 | 2.73E-14 | 0.10 | -3.28 | 3.28 |
| LOC100132116 | 2.60E-06 | 0.10 | -3.29 | 3.29 |
| PDK4 | 1.73E-10 | 0.10 | -3.30 | 3.30 |
| CCL28 | 2.04E-10 | 0.10 | -3.31 | 3.31 |
| LOC728264 | 4.73E-10 | 0.10 | -3.31 | 3.31 |
| NRG2 | 7.83E-05 | 0.10 | -3.33 | 3.33 |
| LOC349196 | 1.83E-07 | 0.10 | -3.33 | 3.33 |
| CHL1 | 4.01E-07 | 0.10 | -3.36 | 3.36 |
| GSTM2 | 8.46E-10 | 0.10 | -3.37 | 3.37 |
| KIF5C | 3.73E-08 | 0.10 | -3.38 | 3.38 |
| COL14A1 | 1.12E-05 | 0.09 | -3.68 | 3.68 |
| CLIC6 | 0.00574 | 0.09 | -3.45 | 3.45 |
| PTHLH | 0.000252 | 0.09 | -3.45 | 3.45 |
| SCGB1D2 | 4.16E-07 | 0.09 | -3.50 | 3.50 |
| FIGF | 1.19E-05 | 0.08 | -3.57 | 3.57 |
| TP63 | 1.16E-08 | 0.08 | -3.59 | 3.59 |
| SCGB1D1 | 3.28E-07 | 0.08 | -3.60 | 3.60 |
| SCUBE2 | 2.29E-08 | 0.08 | -3.68 | 3.68 |
| PGR | 0.00017 | 0.08 | -3.68 | 3.68 |
| EDN3 | 7.84E-10 | 0.07 | -3.75 | 3.75 |
| RIC3 | 4.07E-13 | 0.07 | -3.84 | 3.84 |
| ANKRD30A | 5.92E-12 | 0.06 | -3.96 | 3.96 |
| NPY2R | 0.000509 | 0.06 | -3.99 | 3.99 |
| SEMA6D | 1.08E-06 | 0.06 | -4.08 | 4.08 |
| GRPR | 9.21E-08 | 0.06 | -4.18 | 4.18 |
| C8orf79 | 1.70E-10 | 0.05 | -4.21 | 4.21 |
| SCN2B | 3.91E-09 | 0.05 | -4.32 | 4.32 |
| PIK3C2G | 4.64E-10 | 0.05 | -4.38 | 4.38 |
| NEK10 | 3.45E-05 | 0.05 | -4.39 | 4.39 |
| OXTR | 1.23E-09 | 0.04 | -4.48 | 4.48 |
| PI15 | 1.35E-09 | 0.04 | -4.55 | 4.55 |
| MYBPC1 | 0.00335 | 0.04 | -4.67 | 4.67 |

OPLS, orthogonal partial least squares; DEG, differentially expressed genes; TNBC, triple negative breast cancer; FC, fold change.

**Supplementary Table 2.** Pathways involved in TNBC.

| Cluster no. | Term name | Term id | FDR |
| --- | --- | --- | --- |
| 1 | Cell Cycle, Mitotic | REAC:R-HSA-69278 | 1.29E-28 |
|  | Cell Cycle | REAC:R-HSA-1640170 | 1.61E-27 |
|  | Resolution of Sister Chromatid Cohesion | REAC:R-HSA-2500257 | 7.45E-21 |
|  | Mitotic Prometaphase | REAC:R-HSA-68877 | 1.71E-20 |
|  | M Phase | REAC:R-HSA-68886 | 1.78E-20 |
|  | Mitotic Anaphase | REAC:R-HSA-68882 | 2.18E-19 |
|  | Mitotic Metaphase and Anaphase | REAC:R-HSA-2555396 | 2.37E-19 |
|  | Cell cycle | KEGG:04110 | 3.51E-18 |
|  | Cell Cycle Checkpoints | REAC:R-HSA-69620 | 2.08E-17 |
|  | Mitotic Spindle Checkpoint | REAC:R-HSA-69618 | 3.25E-16 |
|  | Separation of Sister Chromatids | REAC:R-HSA-2467813 | 5.87E-16 |
|  | Amplification of signal from the kinetochores | REAC:R-HSA-141424 | 1.87E-15 |
|  | Amplification of signal from unattached kinetochores via a MAD2 inhibitory signal | REAC:R-HSA-141444 | 1.87E-15 |
|  | EML4 and NUDC in mitotic spindle formation | REAC:R-HSA-9648025 | 3.88E-14 |
|  | RHO GTPases Activate Formins | REAC:R-HSA-5663220 | 3.66E-13 |
|  | RHO GTPase Effectors | REAC:R-HSA-195258 | 6.12E-11 |
|  | APC/C-mediated degradation of cell cycle proteins | REAC:R-HSA-174143 | 1.94E-10 |
|  | Regulation of mitotic cell cycle | REAC:R-HSA-453276 | 1.94E-10 |
|  | Signaling by Rho GTPases | REAC:R-HSA-194315 | 4.24E-08 |
|  | Signaling by Rho GTPases, Miro GTPases and RHOBTB3 | REAC:R-HSA-9716542 | 5.91E-08 |
|  | G2/M Transition | REAC:R-HSA-69275 | 5.98E-07 |
|  | Mitotic G2-G2/M phases | REAC:R-HSA-453274 | 6.60E-07 |
| 2 | Cell Cycle | REAC:R-HSA-1640170 | 2.90E-18 |
|  | Disease | REAC:R-HSA-1643685 | 2.11E-09 |
|  | DNA Replication | REAC:R-HSA-69306 | 1.37E-28 |
|  | Cell Cycle, Mitotic | REAC:R-HSA-69278 | 1.31E-18 |
|  | Metabolism of proteins | REAC:R-HSA-392499 | 1.74E-07 |
|  | Viral Infection Pathways | REAC:R-HSA-9824446 | 1.10E-13 |
|  | Infectious disease | REAC:R-HSA-5663205 | 6.52E-12 |
|  | DNA Repair | REAC:R-HSA-73894 | 1.21E-19 |
|  | Post-translational protein modification | REAC:R-HSA-597592 | 6.80E-07 |
|  | Gene expression (Transcription) | REAC:R-HSA-74160 | 1.56251E-06 |
|  | Systemic lupus erythematosus | KEGG:05322 | 5.32E-25 |
|  | Alcoholism | KEGG:05034 | 2.88E-22 |
|  | Neutrophil extracellular trap formation | KEGG:04613 | 3.49E-22 |
|  | DNA Replication Pre-Initiation | REAC:R-HSA-69002 | 1.04E-21 |
|  | Chromatin organization | REAC:R-HSA-4839726 | 1.17E-17 |
|  | Chromatin modifying enzymes | REAC:R-HSA-3247509 | 1.17E-17 |
|  | Cellular responses to stress | REAC:R-HSA-2262752 | 5.23E-10 |
|  | Cellular responses to stimuli | REAC:R-HSA-8953897 | 6.98E-10 |
|  | Developmental Biology | REAC:R-HSA-1266738 | 2.16E-07 |
|  | Generic Transcription Pathway | REAC:R-HSA-212436 | 7.63E-07 |
|  | RNA Polymerase II Transcription | REAC:R-HSA-73857 | 3.23285E-06 |
|  | Signal Transduction | REAC:R-HSA-162582 | 0.029967748 |
|  | DNA methylation | REAC:R-HSA-5334118 | 1.68E-26 |
|  | Defective pyroptosis | REAC:R-HSA-9710421 | 1.35E-25 |
|  | HDACs deacetylate histones | REAC:R-HSA-3214815 | 1.15E-23 |
|  | Diseases of programmed cell death | REAC:R-HSA-9645723 | 6.55E-23 |
|  | HCMV Late Events | REAC:R-HSA-9610379 | 2.71E-22 |
|  | HCMV Early Events | REAC:R-HSA-9609690 | 5.48E-21 |
|  | HATs acetylate histones | REAC:R-HSA-3214847 | 1.14E-20 |
|  | Assembly of the pre-replicative complex | REAC:R-HSA-68867 | 1.44E-20 |
|  | Epigenetic regulation of gene expression | REAC:R-HSA-212165 | 2.28E-20 |
|  | Estrogen-dependent gene expression | REAC:R-HSA-9018519 | 2.56E-20 |
|  | HCMV Infection | REAC:R-HSA-9609646 | 8.43E-20 |
|  | ESR-mediated signaling | REAC:R-HSA-8939211 | 1.41E-17 |
|  | TCF dependent signaling in response to WNT | REAC:R-HSA-201681 | 4.18E-17 |
|  | Signaling by NOTCH | REAC:R-HSA-157118 | 4.49E-17 |
|  | Signaling by Nuclear Receptors | REAC:R-HSA-9006931 | 1.63E-15 |
|  | Signaling by WNT | REAC:R-HSA-195721 | 1.11E-14 |
|  | RNA Polymerase I Promoter Opening | REAC:R-HSA-73728 | 2.28E-24 |
|  | Activated PKN1 stimulates transcription of AR (androgen receptor) regulated genes KLK2 and KLK3 | REAC:R-HSA-5625886 | 6.41E-24 |
|  | SIRT1 negatively regulates rRNA expression | REAC:R-HSA-427359 | 8.22E-24 |
|  | Assembly of the ORC complex at the origin of replication | REAC:R-HSA-68616 | 1.05E-23 |
|  | PRC2 methylates histones and DNA | REAC:R-HSA-212300 | 2.68E-23 |
|  | Condensation of Prophase Chromosomes | REAC:R-HSA-2299718 | 2.68E-23 |
|  | ERCC6 (CSB) and EHMT2 (G9a) positively regulate rRNA expression | REAC:R-HSA-427389 | 5.21E-23 |
|  | Meiotic recombination | REAC:R-HSA-912446 | 4.75E-22 |
|  | Transcriptional regulation of granulopoiesis | REAC:R-HSA-9616222 | 6.88E-22 |
|  | B-WICH complex positively regulates rRNA expression | REAC:R-HSA-5250924 | 8.24E-22 |
|  | RNA Polymerase I Promoter Escape | REAC:R-HSA-73772 | 9.85E-22 |
|  | Formation of the beta-catenin:TCF transactivating complex | REAC:R-HSA-201722 | 9.85E-22 |
|  | Pre-NOTCH Transcription and Translation | REAC:R-HSA-1912408 | 1.40E-21 |
|  | RHO GTPases activate PKNs | REAC:R-HSA-5625740 | 1.66E-21 |
|  | RUNX1 regulates genes involved in megakaryocyte differentiation and platelet function | REAC:R-HSA-8936459 | 2.76E-21 |
|  | Transcriptional regulation by small RNAs | REAC:R-HSA-5578749 | 7.22E-21 |
|  | Positive epigenetic regulation of rRNA expression | REAC:R-HSA-5250913 | 9.82E-21 |
|  | NoRC negatively regulates rRNA expression | REAC:R-HSA-427413 | 1.14E-20 |
|  | Negative epigenetic regulation of rRNA expression | REAC:R-HSA-5250941 | 1.78E-20 |
|  | Pre-NOTCH Expression and Processing | REAC:R-HSA-1912422 | 1.78E-20 |
|  | Amyloid fiber formation | REAC:R-HSA-977225 | 2.06E-20 |
|  | RNA Polymerase I Promoter Clearance | REAC:R-HSA-73854 | 2.06E-20 |
|  | RNA Polymerase I Transcription | REAC:R-HSA-73864 | 2.38E-20 |
|  | Senescence-Associated Secretory Phenotype (SASP) | REAC:R-HSA-2559582 | 2.74E-20 |
|  | Telomere Maintenance | REAC:R-HSA-157579 | 2.74E-20 |
|  | Meiosis | REAC:R-HSA-1500620 | 5.48E-20 |
|  | Activation of HOX genes during differentiation | REAC:R-HSA-5619507 | 8.18E-20 |
|  | Activation of anterior HOX genes in hindbrain development during early embryogenesis | REAC:R-HSA-5617472 | 8.18E-20 |
|  | Oxidative Stress Induced Senescence | REAC:R-HSA-2559580 | 1.21E-19 |
|  | RUNX1 regulates transcription of genes involved in differentiation of HSCs | REAC:R-HSA-8939236 | 2.89E-19 |
|  | Mitotic Prophase | REAC:R-HSA-68875 | 5.22E-19 |
|  | Gene Silencing by RNA | REAC:R-HSA-211000 | 5.22E-19 |
|  | Chromosome Maintenance | REAC:R-HSA-73886 | 8.24E-19 |
|  | Reproduction | REAC:R-HSA-1474165 | 1.28E-18 |
|  | Cellular Senescence | REAC:R-HSA-2559583 | 1.55E-16 |
|  | Transcriptional regulation by RUNX1 | REAC:R-HSA-8878171 | 2.83E-15 |
|  | RHO GTPase Effectors | REAC:R-HSA-195258 | 2.62E-13 |
|  | M Phase | REAC:R-HSA-68886 | 1.01E-11 |
|  | Signaling by Rho GTPases | REAC:R-HSA-194315 | 2.58E-08 |
|  | Signaling by Rho GTPases, Miro GTPases and RHOBTB3 | REAC:R-HSA-9716542 | 3.48E-08 |
|  | Base Excision Repair | REAC:R-HSA-73884 | 9.23E-20 |
|  | DNA Double-Strand Break Repair | REAC:R-HSA-5693532 | 1.02E-15 |
|  | Ub-specific processing proteases | REAC:R-HSA-5689880 | 5.02E-14 |
|  | Deubiquitination | REAC:R-HSA-5688426 | 3.16E-12 |
|  | Packaging Of Telomere Ends | REAC:R-HSA-171306 | 5.12E-21 |
|  | Cleavage of the damaged purine | REAC:R-HSA-110331 | 1.17E-20 |
|  | Depurination | REAC:R-HSA-73927 | 1.17E-20 |
|  | Recognition and association of DNA glycosylase with site containing an affected purine | REAC:R-HSA-110330 | 1.17E-20 |
|  | Recognition and association of DNA glycosylase with site containing an affected pyrimidine | REAC:R-HSA-110328 | 4.12E-20 |
|  | Cleavage of the damaged pyrimidine | REAC:R-HSA-110329 | 4.12E-20 |
|  | Depyrimidination | REAC:R-HSA-73928 | 4.12E-20 |
|  | Base-Excision Repair, AP Site Formation | REAC:R-HSA-73929 | 5.23E-20 |
|  | Inhibition of DNA recombination at telomere | REAC:R-HSA-9670095 | 2.00E-19 |
|  | Deposition of new CENPA-containing nucleosomes at the centromere | REAC:R-HSA-606279 | 6.75E-19 |
|  | Nucleosome assembly | REAC:R-HSA-774815 | 6.75E-19 |
|  | Meiotic synapsis | REAC:R-HSA-1221632 | 1.43E-18 |
|  | DNA Damage/Telomere Stress Induced Senescence | REAC:R-HSA-2559586 | 2.44E-18 |
|  | Homology Directed Repair | REAC:R-HSA-5693538 | 4.20E-15 |
|  | Processing of DNA double-strand break ends | REAC:R-HSA-5693607 | 3.21E-15 |
|  | HDR through Homologous Recombination (HRR) or Single Strand Annealing (SSA) | REAC:R-HSA-5693567 | 1.49E-13 |
|  | G2/M Checkpoints | REAC:R-HSA-69481 | 1.15E-10 |
|  | Cell Cycle Checkpoints | REAC:R-HSA-69620 | 4.52E-08 |
|  | DNA Double Strand Break Response | REAC:R-HSA-5693606 | 1.78E-12 |
| 9 | Disease | REAC:R-HSA-1643685 | 1.20791E-06 |
|  | Signal Transduction | REAC:R-HSA-162582 | 8.9584E-05 |
| 12 | Cell Cycle | REAC:R-HSA-1640170 | 1.19E-09 |
|  | Cell Cycle, Mitotic | REAC:R-HSA-69278 | 8.12E-09 |

TNBC, triple negative breast cancer; FDR, false discovery rate.

**Supplementary Table 3.** Biological processes involved in TNBC.

| Cluster no. | Term name | Term id | FDR |
| --- | --- | --- | --- |
| 1 | nuclear chromosome segregation | GO:0098813 | 1.48E-54 |
|  | chromosome segregation | GO:0007059 | 4.31E-52 |
|  | cell cycle process | GO:0022402 | 7.48E-51 |
|  | mitotic cell cycle process | GO:1903047 | 5.54E-50 |
|  | mitotic sister chromatid segregation | GO:0000070 | 9.07E-49 |
|  | mitotic cell cycle | GO:0000278 | 1.06E-48 |
|  | sister chromatid segregation | GO:0000819 | 4.38E-48 |
|  | nuclear division | GO:0000280 | 8.20E-47 |
|  | cell cycle | GO:0007049 | 8.02E-46 |
|  | mitotic nuclear division | GO:0140014 | 3.16E-45 |
|  | organelle fission | GO:0048285 | 3.93E-45 |
|  | cell division | GO:0051301 | 3.81E-42 |
|  | chromosome organization | GO:0051276 | 7.14E-41 |
|  | regulation of cell cycle | GO:0051726 | 4.80E-39 |
|  | regulation of cell cycle process | GO:0010564 | 5.88E-37 |
|  | microtubule cytoskeleton organization involved in mitosis | GO:1902850 | 2.55E-30 |
|  | regulation of chromosome segregation | GO:0051983 | 1.06E-26 |
|  | spindle organization | GO:0007051 | 1.53E-26 |
|  | microtubule cytoskeleton organization | GO:0000226 | 3.06E-26 |
|  | mitotic cell cycle phase transition | GO:0044772 | 1.08E-25 |
|  | regulation of mitotic nuclear division | GO:0007088 | 1.82E-25 |
|  | cell cycle phase transition | GO:0044770 | 5.02E-25 |
|  | regulation of chromosome separation | GO:1905818 | 5.99E-25 |
|  | regulation of mitotic sister chromatid separation | GO:0010965 | 2.07E-24 |
|  | regulation of sister chromatid segregation | GO:0033045 | 2.42E-24 |
|  | mitotic spindle organization | GO:0007052 | 2.45E-24 |
|  | chromosome separation | GO:0051304 | 3.03E-24 |
|  | mitotic sister chromatid separation | GO:0051306 | 4.88E-24 |
|  | regulation of nuclear division | GO:0051783 | 1.10E-23 |
|  | regulation of mitotic metaphase/anaphase transition | GO:0030071 | 2.38E-23 |
|  | microtubule-based process | GO:0007017 | 2.60E-23 |
|  | regulation of metaphase/anaphase transition of cell cycle | GO:1902099 | 4.20E-23 |
|  | metaphase/anaphase transition of mitotic cell cycle | GO:0007091 | 5.05E-23 |
|  | metaphase/anaphase transition of cell cycle | GO:0044784 | 8.68E-23 |
|  | regulation of chromosome organization | GO:0033044 | 9.63E-23 |
|  | regulation of mitotic sister chromatid segregation | GO:0033047 | 9.79E-23 |
|  | regulation of mitotic cell cycle phase transition | GO:1901990 | 5.68E-22 |
|  | organelle organization | GO:0006996 | 7.69E-22 |
|  | chromosome localization | GO:0050000 | 1.38E-21 |
|  | mitotic spindle assembly checkpoint signaling | GO:0007094 | 1.70E-21 |
|  | spindle assembly checkpoint signaling | GO:0071173 | 1.70E-21 |
|  | mitotic spindle checkpoint signaling | GO:0071174 | 1.70E-21 |
|  | regulation of mitotic cell cycle | GO:0007346 | 1.97E-21 |
|  | spindle checkpoint signaling | GO:0031577 | 2.37E-21 |
|  | negative regulation of sister chromatid segregation | GO:0033046 | 3.27E-21 |
|  | negative regulation of mitotic metaphase/anaphase transition | GO:0045841 | 3.27E-21 |
|  | negative regulation of mitotic sister chromatid separation | GO:2000816 | 3.27E-21 |
|  | negative regulation of mitotic sister chromatid segregation | GO:0033048 | 3.27E-21 |
|  | regulation of cell cycle phase transition | GO:1901987 | 3.88E-21 |
|  | metaphase chromosome alignment | GO:0051310 | 5.14E-21 |
|  | negative regulation of metaphase/anaphase transition of cell cycle | GO:1902100 | 6.09E-21 |
|  | negative regulation of chromosome segregation | GO:0051985 | 6.09E-21 |
|  | negative regulation of chromosome separation | GO:1905819 | 6.09E-21 |
|  | spindle assembly | GO:0051225 | 1.44E-20 |
|  | negative regulation of mitotic nuclear division | GO:0045839 | 3.33E-20 |
|  | establishment of chromosome localization | GO:0051303 | 4.18E-20 |
|  | negative regulation of nuclear division | GO:0051784 | 1.89E-19 |
|  | positive regulation of cell cycle process | GO:0090068 | 3.40E-19 |
|  | negative regulation of chromosome organization | GO:2001251 | 7.78E-19 |
|  | non-membrane-bounded organelle assembly | GO:0140694 | 1.06E-18 |
|  | cytoskeleton organization | GO:0007010 | 5.67E-18 |
|  | attachment of spindle microtubules to kinetochore | GO:0008608 | 5.31E-17 |
|  | regulation of organelle organization | GO:0033043 | 8.19E-17 |
|  | positive regulation of cell cycle | GO:0045787 | 9.85E-17 |
|  | organelle localization | GO:0051640 | 1.56E-16 |
|  | mitotic cell cycle checkpoint signaling | GO:0007093 | 2.83E-16 |
|  | cell cycle checkpoint signaling | GO:0000075 | 4.72E-16 |
|  | establishment of organelle localization | GO:0051656 | 7.84E-16 |
|  | negative regulation of mitotic cell cycle phase transition | GO:1901991 | 7.85E-15 |
|  | negative regulation of cell cycle process | GO:0010948 | 1.04E-14 |
|  | mitotic spindle assembly | GO:0090307 | 2.75E-14 |
|  | negative regulation of cell cycle phase transition | GO:1901988 | 3.61E-14 |
|  | mitotic metaphase chromosome alignment | GO:0007080 | 1.85E-13 |
|  | negative regulation of mitotic cell cycle | GO:0045930 | 2.41E-13 |
|  | negative regulation of cell cycle | GO:0045786 | 5.26E-13 |
|  | cytokinesis | GO:0000910 | 7.62E-13 |
|  | negative regulation of organelle organization | GO:0010639 | 4.61E-12 |
|  | mitotic cytokinesis | GO:0000281 | 1.70E-11 |
|  | regulation of cytokinesis | GO:0032465 | 2.15E-11 |
|  | organelle assembly | GO:0070925 | 3.69E-11 |
|  | regulation of cellular component organization | GO:0051128 | 5.32E-11 |
|  | cytoskeleton-dependent cytokinesis | GO:0061640 | 3.38E-10 |
|  | regulation of cell division | GO:0051302 | 8.23E-10 |
|  | cellular component organization | GO:0016043 | 9.16E-10 |
|  | meiotic nuclear division | GO:0140013 | 1.04E-09 |
|  | cellular component organization or biogenesis | GO:0071840 | 2.59E-09 |
|  | meiotic cell cycle process | GO:1903046 | 3.15E-09 |
|  | meiotic cell cycle | GO:0051321 | 3.31E-09 |
|  | cell cycle G2/M phase transition | GO:0044839 | 4.59E-09 |
|  | negative regulation of cellular component organization | GO:0051129 | 6.97E-09 |
|  | protein phosphorylation | GO:0006468 | 6.7028E-06 |
|  | cellular localization | GO:0051641 | 8.0046E-06 |
|  | intracellular signal transduction | GO:0035556 | 8.4537E-06 |
|  | phosphorylation | GO:0016310 | 1.0361E-05 |
|  | establishment of localization in cell | GO:0051649 | 5.8748E-05 |
|  | regulation of transferase activity | GO:0051338 | 8.6796E-05 |
|  | sexual reproduction | GO:0019953 | 0.00020265 |
|  | regulation of cellular process | GO:0050794 | 0.00030754 |
|  | cellular component assembly | GO:0022607 | 0.00034979 |
|  | regulation of protein modification process | GO:0031399 | 0.00077226 |
|  | cellular component biogenesis | GO:0044085 | 0.00123336 |
|  | regulation of biological process | GO:0050789 | 0.00194226 |
|  | phosphate-containing compound metabolic process | GO:0006796 | 0.0023191 |
|  | phosphorus metabolic process | GO:0006793 | 0.00268657 |
|  | localization | GO:0051179 | 0.00395214 |
|  | regulation of protein phosphorylation | GO:0001932 | 0.00571764 |
|  | biological regulation | GO:0065007 | 0.0069601 |
|  | reproductive process | GO:0022414 | 0.0135664 |
|  | reproduction | GO:0000003 | 0.01462869 |
|  | regulation of phosphorylation | GO:0042325 | 0.01536698 |
|  | protein modification process | GO:0036211 | 0.0229894 |
|  | gamete generation | GO:0007276 | 0.0246775 |
|  | positive regulation of cellular process | GO:0048522 | 0.02774855 |
|  | DNA metabolic process | GO:0006259 | 0.04468125 |
| 2 | DNA metabolic process | GO:0006259 | 1.2659E-05 |
|  | chromatin remodeling | GO:0006338 | 2.3079E-05 |
|  | chromatin organization | GO:0006325 | 0.00013177 |
|  | protein-DNA complex organization | GO:0071824 | 0.00038826 |
| 12 | organelle organization | GO:0006996 | 0.03624243 |

TNBC, triple negative breast cancer; FDR, false discovery rate.

**Supplementary Table 4.** Molecular functions involved in TNBC.

| Term name | Term id | FDR |
| --- | --- | --- |
| protein binding | GO:0005515 | 1.46E-24 |
| adenyl nucleotide binding | GO:0030554 | 6.13E-11 |
| carbohydrate derivative binding | GO:0097367 | 2.30E-10 |
| small molecule binding | GO:0036094 | 4.06E-10 |
| ATP binding | GO:0005524 | 5.13E-10 |
| adenyl ribonucleotide binding | GO:0032559 | 5.88E-10 |
| nucleotide binding | GO:0000166 | 7.27E-10 |
| purine nucleotide binding | GO:0017076 | 7.49E-10 |
| nucleoside phosphate binding | GO:1901265 | 7.54E-10 |
| anion binding | GO:0043168 | 2.34E-09 |
| ATP-dependent activity | GO:0140657 | 2.88E-09 |
| purine ribonucleoside triphosphate binding | GO:0035639 | 5.28E-09 |
| purine ribonucleotide binding | GO:0032555 | 6.61E-09 |
| ribonucleotide binding | GO:0032553 | 1.21E-08 |
| ATP hydrolysis activity | GO:0016887 | 1.16E-07 |
| catalytic activity | GO:0003824 | 1.87077E-05 |
| binding | GO:0005488 | 2.92379E-05 |
| microtubule binding | GO:0008017 | 2.97036E-05 |
| tubulin binding | GO:0015631 | 3.71751E-05 |
| microtubule motor activity | GO:0003777 | 5.42164E-05 |
| ribonucleoside triphosphate phosphatase activity | GO:0017111 | 6.35421E-05 |
| single-stranded DNA binding | GO:0003697 | 6.95577E-05 |
| pyrophosphatase activity | GO:0016462 | 8.64572E-05 |
| hydrolase activity, acting on acid anhydrides | GO:0016817 | 9.03999E-05 |
| hydrolase activity, acting on acid anhydrides, in phosphorus-containing anhydrides | GO:0016818 | 9.03999E-05 |
| cyclin-dependent protein serine/threonine kinase regulator activity | GO:0016538 | 9.45791E-05 |
| DNA secondary structure binding | GO:0000217 | 0.000109087 |
| ATP-dependent activity, acting on DNA | GO:0008094 | 0.000444052 |
| cytoskeletal protein binding | GO:0008092 | 0.000838098 |
| DNA helicase activity | GO:0003678 | 0.000859418 |
| cytoskeletal motor activity | GO:0003774 | 0.001610903 |
| enzyme binding | GO:0019899 | 0.002333642 |
| kinase activity | GO:0016301 | 0.00236054 |
| identical protein binding | GO:0042802 | 0.002392296 |
| oxidoreductase activity | GO:0016491 | 0.010913027 |
| catalytic activity, acting on DNA | GO:0140097 | 0.013620706 |
| protein-containing complex binding | GO:0044877 | 0.027325484 |
| protein homodimerization activity | GO:0042803 | 0.048163583 |

TNBC, triple negative breast cancer; FDR, false discovery rate.

**Supplementary Table 5.** Cellular components involved in TNBC.

| Term name | Term id | FDR |
| --- | --- | --- |
| cellular anatomical entity | GO:0110165 | 0.000276844 |
| intracellular anatomical structure | GO:0005622 | 2.44958E-06 |
| organelle | GO:0043226 | 9.31E-09 |
| membrane-bounded organelle | GO:0043227 | 2.13E-08 |
| intracellular organelle | GO:0043229 | 6.33903E-06 |
| cytoplasm | GO:0005737 | 8.31E-28 |
| intracellular membrane-bounded organelle | GO:0043231 | 0.000155322 |
| intracellular organelle lumen | GO:0070013 | 1.38E-11 |
| organelle lumen | GO:0043233 | 1.38E-11 |
| membrane-enclosed lumen | GO:0031974 | 1.38E-11 |
| intracellular non-membrane-bounded organelle | GO:0043232 | 3.89E-08 |
| non-membrane-bounded organelle | GO:0043228 | 3.97E-08 |
| cytosol | GO:0005829 | 1.26E-16 |
| nuclear lumen | GO:0031981 | 3.85333E-05 |
| nucleoplasm | GO:0005654 | 1.79E-14 |
| extracellular region | GO:0005576 | 3.97E-07 |
| vesicle | GO:0031982 | 0.000825905 |
| extracellular space | GO:0005615 | 1.17E-07 |
| cytoskeleton | GO:0005856 | 2.56E-11 |
| chromosome | GO:0005694 | 5.01E-18 |
| extracellular vesicle | GO:1903561 | 2.35E-07 |
| extracellular organelle | GO:0043230 | 2.42E-07 |
| extracellular membrane-bounded organelle | GO:0065010 | 2.42E-07 |
| extracellular exosome | GO:0070062 | 2.26E-07 |
| microtubule cytoskeleton | GO:0015630 | 3.17E-14 |
| supramolecular complex | GO:0099080 | 2.20E-07 |
| catalytic complex | GO:1902494 | 0.01570076 |
| mitochondrion | GO:0005739 | 0.00412659 |
| protein-DNA complex | GO:0032993 | 0.000178975 |
| organelle envelope | GO:0031967 | 3.92997E-06 |
| envelope | GO:0031975 | 3.92997E-06 |
| chromatin | GO:0000785 | 0.003843547 |
| supramolecular fiber | GO:0099512 | 6.86838E-05 |
| supramolecular polymer | GO:0099081 | 9.11522E-05 |
| microtubule organizing center | GO:0005815 | 2.20E-07 |
| chromosomal region | GO:0098687 | 1.97E-20 |
| mitochondrial envelope | GO:0005740 | 0.001237704 |
| spindle | GO:0005819 | 2.36E-13 |
| mitochondrial membrane | GO:0031966 | 0.000951609 |
| polymeric cytoskeletal fiber | GO:0099513 | 0.002115477 |
| centrosome | GO:0005813 | 6.75171E-05 |
| chromosome, centromeric region | GO:0000775 | 5.31E-17 |
| microtubule | GO:0005874 | 8.01E-07 |
| extracellular matrix | GO:0031012 | 7.3731E-05 |
| external encapsulating structure | GO:0030312 | 7.75348E-05 |
| condensed chromosome | GO:0000793 | 1.89E-13 |
| organelle inner membrane | GO:0019866 | 0.000848683 |
| collagen-containing extracellular matrix | GO:0062023 | 1.8403E-05 |
| mitochondrial inner membrane | GO:0005743 | 0.002312162 |
| mitochondrial matrix | GO:0005759 | 0.006475212 |
| condensed chromosome, centromeric region | GO:0000779 | 6.09E-13 |
| mitotic spindle | GO:0072686 | 1.67E-12 |
| kinetochore | GO:0000776 | 2.80E-11 |
| nuclear chromosome | GO:0000228 | 7.80E-07 |
| mitochondrial protein-containing complex | GO:0098798 | 0.000810259 |
| midbody | GO:0030496 | 0.000847717 |
| spindle pole | GO:0000922 | 0.000160231 |
| microtubule associated complex | GO:0005875 | 0.000771271 |
| intercellular bridge | GO:0045171 | 9.03E-07 |
| chromosome, telomeric region | GO:0000781 | 0.007625573 |
| replication fork | GO:0005657 | 8.46845E-06 |
| kinesin complex | GO:0005871 | 1.71181E-06 |
| outer kinetochore | GO:0000940 | 4.87E-08 |
| spindle midzone | GO:0051233 | 0.000104899 |
| cyclin-dependent protein kinase holoenzyme complex | GO:0000307 | 0.005044828 |

TNBC, triple negative breast cancer; FDR, false discovery rate.

**Supplementary Table 6**

The GEO2R indicated 3895 DEGs in primary TNBC compared to healthy tissues with a criterion of FDR < 0.01 and |Log2 FC| > 1.585.

| **Symbol** | **FDR** | **log2. FC** |
| --- | --- | --- |
| CST1 | 1.48E-27 | 9.32 |
| IBSP | 1.99E-34 | 8.00 |
| TLX3 | 1.46E-27 | 7.78 |
| MMP13 | 8.29E-34 | 7.50 |
| PRAME | 2.95E-44 | 7.37 |
| MMP1 | 6.13E-32 | 7.17 |
| LINC01667 | 2.21E-18 | 7.00 |
| CST4 | 2.11E-20 | 6.92 |
| NCAN | 6.87E-20 | 6.68 |
| LINC02438 | 4.94E-17 | 6.59 |
| HORMAD1 | 8.51E-30 | 6.28 |
| LINC01705 | 6.01E-21 | 6.27 |
| HOXB13 | 9.03E-17 | 6.24 |
| HAPLN1 | 1.17E-18 | 6.22 |
| LINC01050 | 4.08E-23 | 6.19 |
| COL10A1 | 6.96E-45 | 6.16 |
| LINC00518 | 2.32E-25 | 6.08 |
| CCKBR | 1.19E-23 | 6.05 |
| CXCL13 | 7.10E-26 | 5.89 |
| KIF1A | 2.56E-26 | 5.82 |
| CA9 | 3.08E-23 | 5.80 |
| COL22A1 | 1.70E-23 | 5.79 |
| DPYSL5 | 1.33E-20 | 5.78 |
| LINC02487 | 7.76E-21 | 5.74 |
| POU4F1 | 1.95E-28 | 5.74 |
| S100A7 | 3.27E-13 | 5.74 |
| TLX1 | 5.76E-16 | 5.71 |
| LINC01614 | 4.17E-50 | 5.68 |
| PRAC2 | 1.14E-13 | 5.63 |
| LOC101060400 | 2.61E-18 | 5.63 |
| H3C12 | 3.55E-20 | 5.59 |
| LOC107984024 | 3.73E-14 | 5.57 |
| H4C4 | 1.73E-21 | 5.51 |
| SBSN | 1.41E-22 | 5.48 |
| LHX2 | 3.91E-26 | 5.42 |
| MMP11 | 7.38E-35 | 5.38 |
| CT83 | 7.62E-13 | 5.35 |
| TCL1A | 4.46E-13 | 5.28 |
| SIX3 | 5.89E-27 | 5.27 |
| ASPM | 9.67E-65 | 5.26 |
| INA | 6.89E-23 | 5.24 |
| OR2B6 | 1.20E-22 | 5.24 |
| DMP1 | 6.90E-18 | 5.21 |
| CHODL-AS1 | 8.95E-16 | 5.21 |
| LINC02990 | 3.69E-25 | 5.17 |
| MAFA-AS1 | 4.07E-17 | 5.13 |
| MS4A1 | 3.54E-13 | 5.12 |
| KIF14 | 1.52E-62 | 5.12 |
| LCAL1 | 4.25E-15 | 5.08 |
| MNX1-AS2 | 6.14E-18 | 5.08 |
| LINC01833 | 1.01E-23 | 5.07 |
| LINC02437 | 7.25E-14 | 5.03 |
| VGF | 1.59E-31 | 4.99 |
| KIF26B-AS1 | 4.60E-26 | 4.98 |
| TMEM270 | 2.46E-20 | 4.97 |
| MSLN | 9.14E-17 | 4.97 |
| FNDC1-AS1 | 9.08E-25 | 4.93 |
| TLX1NB | 5.35E-11 | 4.92 |
| CENPF | 3.12E-72 | 4.91 |
| KCNQ5-IT1 | 7.38E-18 | 4.90 |
| CD19 | 7.66E-17 | 4.89 |
| LEMD1-AS1 | 7.08E-20 | 4.89 |
| SIX3-AS1 | 5.94E-16 | 4.85 |
| NUF2 | 4.84E-60 | 4.84 |
| LEMD1 | 6.21E-18 | 4.82 |
| PRSS33 | 1.85E-10 | 4.79 |
| H2AC17 | 3.43E-31 | 4.77 |
| LINC00393 | 7.81E-14 | 4.76 |
| NOTUM | 4.06E-17 | 4.76 |
| UBE2C | 3.05E-46 | 4.76 |
| FCRL1 | 2.92E-12 | 4.76 |
| CLDN6 | 1.41E-14 | 4.74 |
| MUC5AC | 1.19E-16 | 4.74 |
| TCAM1P | 1.13E-25 | 4.70 |
| SGO1-AS1 | 9.64E-68 | 4.68 |
| ACAN | 5.07E-17 | 4.65 |
| PRR27 | 2.77E-08 | 4.65 |
| COL11A1 | 3.45E-25 | 4.65 |
| IQGAP3 | 2.53E-42 | 4.65 |
| MKI67 | 2.92E-67 | 4.65 |
| CKAP2L | 5.47E-66 | 4.62 |
| LMO1 | 2.72E-14 | 4.62 |
| CLPSL1 | 3.29E-13 | 4.62 |
| MIR6783 | 3.07E-21 | 4.61 |
| SEC14L4 | 1.79E-19 | 4.61 |
| EPYC | 1.96E-17 | 4.60 |
| LINC01429 | 4.74E-25 | 4.59 |
| CLDN9 | 6.10E-24 | 4.58 |
| KIF4A | 8.81E-63 | 4.57 |
| H2BC7 | 2.16E-18 | 4.56 |
| MOG | 4.62E-14 | 4.56 |
| GABBR2 | 6.73E-18 | 4.56 |
| CCDC150 | 1.70E-38 | 4.54 |
| HMMR-AS1 | 1.16E-51 | 4.53 |
| LINC00461 | 5.40E-16 | 4.52 |
| CDCA2 | 3.59E-47 | 4.51 |
| MYBL2 | 1.20E-51 | 4.49 |
| TRPM8 | 1.13E-10 | 4.48 |
| EDN2 | 8.09E-19 | 4.45 |
| SPIB | 1.13E-15 | 4.43 |
| PLPP4 | 1.22E-27 | 4.42 |
| H2BC9 | 5.32E-22 | 4.42 |
| FSD1 | 1.92E-23 | 4.42 |
| NEK2 | 3.90E-36 | 4.42 |
| HJURP | 1.70E-55 | 4.40 |
| NDC80 | 2.96E-51 | 4.40 |
| LINC02099 | 5.45E-23 | 4.38 |
| LINC01297 | 2.31E-16 | 4.38 |
| LOC112267886 | 3.96E-21 | 4.37 |
| RUNX1-IT1 | 8.89E-38 | 4.37 |
| TERT | 1.56E-18 | 4.37 |
| HMMR | 1.55E-54 | 4.37 |
| FOXCUT | 5.50E-25 | 4.36 |
| NPHS1 | 4.11E-13 | 4.36 |
| LINC02678 | 7.68E-14 | 4.35 |
| CASP14 | 2.73E-09 | 4.35 |
| CENPE | 5.34E-59 | 4.34 |
| SLC26A9 | 2.65E-19 | 4.34 |
| LINC02532 | 1.95E-10 | 4.33 |
| FBN3 | 6.91E-18 | 4.33 |
| SERPINB2 | 1.91E-11 | 4.32 |
| TROAP | 8.71E-48 | 4.31 |
| GTSE1 | 3.23E-61 | 4.30 |
| IGF2BP3 | 2.61E-18 | 4.30 |
| MNX1 | 2.56E-15 | 4.29 |
| CCL11 | 1.76E-14 | 4.28 |
| A2ML1 | 5.44E-19 | 4.28 |
| SMC1B | 1.90E-28 | 4.28 |
| IGFL2-AS1 | 7.33E-12 | 4.28 |
| LINC00673 | 2.02E-24 | 4.27 |
| H3C4 | 8.76E-29 | 4.27 |
| CST2 | 8.17E-14 | 4.27 |
| ANLN | 1.55E-38 | 4.26 |
| CEP55 | 3.36E-51 | 4.26 |
| CENPA | 5.74E-42 | 4.24 |
| SCEL | 3.88E-12 | 4.24 |
| LINC02732 | 7.41E-15 | 4.22 |
| RIMS2 | 1.55E-16 | 4.22 |
| ISL2 | 4.95E-14 | 4.21 |
| TPX2 | 2.08E-49 | 4.21 |
| TOP2A | 2.15E-49 | 4.21 |
| FCRL2 | 1.73E-10 | 4.20 |
| LOC101929613 | 4.41E-17 | 4.18 |
| H1-3 | 2.23E-20 | 4.18 |
| SLC15A1 | 1.17E-17 | 4.18 |
| SPDYC | 1.79E-15 | 4.17 |
| KIF20A | 1.33E-48 | 4.17 |
| SIM2 | 6.75E-22 | 4.16 |
| KIF18B | 1.47E-41 | 4.16 |
| MYLK2 | 6.34E-27 | 4.14 |
| CDC20 | 3.73E-45 | 4.14 |
| CCDC166 | 3.75E-16 | 4.13 |
| EN1 | 3.19E-20 | 4.13 |
| SALL4 | 8.26E-43 | 4.13 |
| SOX11 | 1.69E-21 | 4.13 |
| HOXC12 | 4.58E-12 | 4.13 |
| BIRC5 | 6.35E-37 | 4.12 |
| ACP7 | 2.00E-16 | 4.12 |
| MELK | 9.66E-44 | 4.12 |
| SNORD3A | 9.74E-17 | 4.11 |
| CLPS | 7.31E-09 | 4.10 |
| TRIML2 | 6.32E-17 | 4.10 |
| LINC00511 | 2.89E-16 | 4.10 |
| KRT81 | 2.31E-09 | 4.10 |
| CELF4 | 9.94E-32 | 4.09 |
| DIAPH3-AS1 | 3.58E-39 | 4.08 |
| MROH3P | 3.85E-15 | 4.08 |
| SGO1 | 1.10E-69 | 4.08 |
| KIF2C | 1.57E-57 | 4.08 |
| BLK | 3.83E-11 | 4.07 |
| CNR2 | 2.35E-12 | 4.07 |
| TTLL8 | 2.15E-19 | 4.06 |
| BARX1 | 2.98E-14 | 4.06 |
| TTK | 2.49E-41 | 4.05 |
| AURKB | 2.59E-48 | 4.04 |
| EFNA2 | 1.11E-14 | 4.04 |
| STRA8 | 1.62E-13 | 4.04 |
| ONECUT2 | 1.40E-26 | 4.02 |
| PTTG1 | 4.47E-50 | 4.02 |
| CLSPN | 1.44E-76 | 4.01 |
| SCGB3A2 | 3.11E-15 | 4.00 |
| FCRLA | 1.36E-10 | 4.00 |
| DLGAP5 | 1.53E-43 | 3.99 |
| S100A8 | 7.69E-11 | 3.99 |
| S100P | 9.20E-14 | 3.98 |
| ZIC1 | 5.48E-17 | 3.98 |
| CDKN2A | 6.41E-23 | 3.98 |
| SMIM45 | 1.75E-39 | 3.98 |
| SNORD3C | 7.88E-15 | 3.97 |
| CXorf49B | 5.24E-13 | 3.97 |
| PGLYRP4 | 2.61E-11 | 3.96 |
| CNIH2 | 1.89E-34 | 3.96 |
| C1QL4 | 2.57E-13 | 3.94 |
| FDCSP | 1.51E-07 | 3.94 |
| TDO2 | 8.78E-17 | 3.94 |
| ARTN | 1.99E-26 | 3.93 |
| POLQ | 5.21E-51 | 3.93 |
| ULBP1 | 1.22E-27 | 3.93 |
| HTR1D | 5.47E-12 | 3.92 |
| TNNT1 | 1.96E-11 | 3.92 |
| ELAVL2 | 3.65E-20 | 3.91 |
| CWH43 | 4.40E-09 | 3.91 |
| XIRP1 | 4.31E-19 | 3.91 |
| SKA1 | 1.53E-48 | 3.91 |
| FAM111B | 3.11E-37 | 3.90 |
| SNORD160 | 1.11E-19 | 3.90 |
| IGF2BP1 | 4.29E-15 | 3.90 |
| PIF1 | 8.07E-39 | 3.90 |
| EPHA8 | 6.92E-13 | 3.90 |
| H1-9P | 1.03E-09 | 3.90 |
| PATE2 | 7.84E-16 | 3.89 |
| GABRQ | 1.40E-10 | 3.89 |
| CXorf49 | 6.96E-14 | 3.89 |
| MIR4653 | 1.18E-22 | 3.89 |
| OPRK1 | 2.89E-15 | 3.88 |
| CDCA8 | 1.34E-42 | 3.88 |
| H2AC7 | 1.91E-17 | 3.87 |
| CHD5 | 1.15E-14 | 3.86 |
| SLC12A5-AS1 | 1.08E-15 | 3.86 |
| NEIL3 | 4.14E-40 | 3.86 |
| XRCC2 | 1.05E-33 | 3.85 |
| KIF15 | 3.02E-53 | 3.85 |
| CALML5 | 7.18E-12 | 3.83 |
| KCNJ6 | 2.41E-16 | 3.83 |
| ZNF695 | 1.34E-24 | 3.83 |
| NT5DC4 | 1.02E-32 | 3.83 |
| IGHGP | 7.10E-11 | 3.82 |
| CFAP47 | 3.85E-12 | 3.82 |
| DLX5 | 3.55E-14 | 3.82 |
| GSDMC | 6.78E-20 | 3.82 |
| SERPINB7 | 6.63E-10 | 3.82 |
| IGHV5-78 | 5.19E-10 | 3.81 |
| KREMEN2 | 4.31E-27 | 3.81 |
| NMU | 1.63E-19 | 3.81 |
| KCNQ5-AS1 | 1.43E-14 | 3.80 |
| CADPS | 3.30E-14 | 3.80 |
| KNL1 | 7.78E-56 | 3.80 |
| RRM2 | 2.26E-39 | 3.80 |
| LINC01276 | 8.97E-12 | 3.78 |
| KLHDC7B-DT | 1.25E-25 | 3.77 |
| PBK | 8.26E-30 | 3.77 |
| BUB1 | 1.07E-40 | 3.77 |
| LOC101929759 | 9.10E-10 | 3.76 |
| EXO1 | 1.53E-30 | 3.75 |
| FOXD1 | 9.30E-13 | 3.74 |
| EPO | 9.40E-14 | 3.74 |
| LHFPL5 | 9.32E-09 | 3.74 |
| LINC00707 | 3.56E-10 | 3.73 |
| AARD | 1.88E-16 | 3.73 |
| LINC02473 | 4.78E-13 | 3.73 |
| PLA2G2D | 1.38E-11 | 3.73 |
| SNORD3B-2 | 5.19E-13 | 3.72 |
| TUBB3 | 1.32E-24 | 3.72 |
| EPIC1 | 1.23E-09 | 3.71 |
| PAX1 | 3.45E-09 | 3.71 |
| LOC105375112 | 1.21E-15 | 3.71 |
| SLURP1 | 3.71E-14 | 3.70 |
| LINC01842 | 1.12E-13 | 3.70 |
| LINC01215 | 1.96E-09 | 3.69 |
| DIAPH3 | 3.02E-53 | 3.69 |
| H4C5 | 2.15E-17 | 3.69 |
| MND1 | 1.05E-42 | 3.68 |
| MUC5B-AS1 | 1.39E-13 | 3.68 |
| NPW | 5.52E-18 | 3.68 |
| ARHGAP11B | 2.76E-37 | 3.68 |
| SPC24 | 7.78E-47 | 3.68 |
| SNORD3D | 1.30E-13 | 3.68 |
| LINC02086 | 2.00E-15 | 3.67 |
| NUSAP1 | 1.49E-50 | 3.67 |
| GREP1 | 2.14E-20 | 3.67 |
| KIF4B | 3.27E-42 | 3.66 |
| GJB6 | 3.91E-09 | 3.66 |
| SNORD3B-1 | 8.33E-13 | 3.66 |
| CDCA3 | 3.26E-50 | 3.66 |
| ORC6 | 1.57E-39 | 3.65 |
| COL2A1 | 4.70E-07 | 3.65 |
| ORM1 | 1.73E-08 | 3.64 |
| GARIN1B | 4.07E-13 | 3.64 |
| MYBL1 | 5.00E-25 | 3.64 |
| ADAMTS14 | 2.18E-25 | 3.64 |
| ECMXP | 2.48E-16 | 3.64 |
| PKMYT1 | 1.32E-42 | 3.63 |
| CLEC18B | 1.98E-15 | 3.63 |
| MYO3B | 2.05E-16 | 3.63 |
| IGHG4 | 1.54E-10 | 3.63 |
| DEPDC1B | 1.74E-49 | 3.62 |
| KRT85 | 2.13E-08 | 3.62 |
| KIFC1 | 1.62E-43 | 3.61 |
| FAM238C | 2.05E-16 | 3.61 |
| FOXM1 | 4.49E-36 | 3.61 |
| MKRN3 | 1.01E-09 | 3.60 |
| NTSR1 | 1.26E-11 | 3.60 |
| H3C10 | 9.95E-18 | 3.60 |
| OTX1 | 2.35E-18 | 3.60 |
| CKS2 | 3.19E-40 | 3.59 |
| VPREB3 | 3.59E-14 | 3.58 |
| LINC02771 | 2.84E-17 | 3.58 |
| DEPDC1 | 5.16E-27 | 3.58 |
| SYCP2 | 1.32E-18 | 3.58 |
| TMEM89 | 3.62E-18 | 3.58 |
| TFR2 | 6.23E-29 | 3.58 |
| DLX6-AS1 | 1.03E-10 | 3.57 |
| DLL3 | 5.96E-15 | 3.57 |
| BUB1B | 4.34E-41 | 3.56 |
| BLACAT1 | 4.45E-15 | 3.55 |
| SKA3 | 1.40E-37 | 3.54 |
| LINC02899 | 1.52E-06 | 3.54 |
| RAD51AP1 | 2.49E-41 | 3.54 |
| DAPK1-IT1 | 1.34E-23 | 3.54 |
| MLC1 | 1.68E-08 | 3.53 |
| DLX6 | 1.56E-11 | 3.53 |
| PLK1 | 3.67E-42 | 3.51 |
| TMCC2-AS1 | 1.52E-15 | 3.51 |
| IGLV3-1 | 9.27E-09 | 3.51 |
| ATP2B3 | 5.87E-12 | 3.50 |
| CD79A | 4.58E-09 | 3.50 |
| FBXO43 | 1.36E-25 | 3.50 |
| KRT82 | 4.18E-07 | 3.50 |
| CD1A | 2.08E-11 | 3.50 |
| LINC01857 | 6.49E-14 | 3.50 |
| PCAT7 | 3.24E-17 | 3.49 |
| CACNA1B | 6.93E-11 | 3.49 |
| LINC01436 | 8.28E-14 | 3.49 |
| CR2 | 3.40E-07 | 3.48 |
| MCM10 | 8.52E-35 | 3.48 |
| GJB2 | 1.69E-11 | 3.47 |
| KRT86 | 4.36E-12 | 3.47 |
| LOC102724943 | 2.76E-22 | 3.47 |
| PRR11 | 1.77E-44 | 3.47 |
| ESCO2 | 3.72E-35 | 3.47 |
| ICMT-DT | 1.50E-15 | 3.46 |
| AURKA | 6.85E-36 | 3.46 |
| HMSD | 4.23E-13 | 3.46 |
| LINC02323 | 2.39E-12 | 3.46 |
| H2AC8 | 1.05E-17 | 3.46 |
| ANKRD62 | 2.83E-11 | 3.45 |
| PCLAF | 4.08E-34 | 3.45 |
| TCF24 | 1.23E-14 | 3.45 |
| P2RX5 | 2.90E-15 | 3.43 |
| PIMREG | 5.62E-26 | 3.42 |
| KCNG1 | 5.62E-14 | 3.42 |
| CCL20 | 9.47E-15 | 3.42 |
| MAST1 | 7.69E-18 | 3.42 |
| KCNQ1OT1 | 8.57E-15 | 3.42 |
| TCHH | 1.01E-11 | 3.42 |
| IGHG3 | 5.74E-10 | 3.42 |
| TMSB15A | 3.56E-15 | 3.41 |
| DUSP5P1 | 8.60E-12 | 3.41 |
| SLC24A2 | 4.12E-20 | 3.41 |
| KRT3 | 4.53E-09 | 3.41 |
| IFNL1 | 1.64E-15 | 3.41 |
| ANTXRLP1 | 3.05E-12 | 3.41 |
| FAM83D | 9.28E-28 | 3.41 |
| MUC16 | 1.53E-10 | 3.41 |
| FAM72B | 2.18E-31 | 3.40 |
| E2F8 | 1.75E-27 | 3.40 |
| TMEM108-AS1 | 3.62E-12 | 3.40 |
| C11orf86 | 1.44E-06 | 3.40 |
| MEX3A | 2.21E-25 | 3.39 |
| ELAVL3 | 9.78E-13 | 3.39 |
| UBE2T | 5.70E-28 | 3.39 |
| CDK1 | 3.22E-34 | 3.39 |
| CDC45 | 2.23E-44 | 3.39 |
| RECQL4 | 5.44E-42 | 3.39 |
| RUFY4 | 8.72E-18 | 3.39 |
| ESPL1 | 2.62E-32 | 3.39 |
| ELFN1-AS1 | 2.33E-11 | 3.39 |
| MYBPH | 1.65E-11 | 3.39 |
| TIAM1-AS1 | 2.55E-19 | 3.39 |
| RDM1 | 1.57E-21 | 3.38 |
| ZAN | 5.51E-10 | 3.37 |
| DLGAP1-AS4 | 1.89E-08 | 3.37 |
| LINC01910 | 9.70E-17 | 3.37 |
| LIX1 | 5.23E-12 | 3.37 |
| NIPAL4 | 3.99E-13 | 3.36 |
| DLGAP1-AS3 | 8.94E-10 | 3.36 |
| MAB21L4 | 6.69E-10 | 3.36 |
| LINC03040 | 9.00E-12 | 3.36 |
| MAPK15 | 2.89E-16 | 3.35 |
| CSMD2 | 9.34E-14 | 3.35 |
| SNORD88C | 9.05E-22 | 3.35 |
| COL9A3 | 8.15E-13 | 3.34 |
| CYP27C1 | 7.14E-13 | 3.34 |
| H2BC17 | 5.78E-20 | 3.34 |
| KLHL34 | 6.16E-08 | 3.34 |
| LRRC37A8P | 2.73E-16 | 3.34 |
| TREX2 | 7.32E-18 | 3.34 |
| SCUBE3 | 5.06E-15 | 3.34 |
| STK33 | 1.30E-11 | 3.34 |
| DNAH17-AS1 | 9.09E-11 | 3.33 |
| LOC105372412 | 1.87E-18 | 3.32 |
| LINC02397 | 6.50E-10 | 3.32 |
| ZFP57 | 6.10E-08 | 3.31 |
| ACRV1 | 1.09E-26 | 3.31 |
| CCL7 | 3.48E-07 | 3.31 |
| OVOS2 | 2.70E-12 | 3.30 |
| GPR158 | 3.16E-13 | 3.30 |
| MIR4641 | 1.88E-16 | 3.30 |
| IGFL2 | 5.80E-11 | 3.29 |
| CENPW | 3.37E-26 | 3.29 |
| LINP1 | 9.77E-07 | 3.29 |
| NKX2-5 | 1.38E-08 | 3.29 |
| LGALS17A | 6.14E-09 | 3.29 |
| LTB | 1.53E-15 | 3.29 |
| PSORS1C2 | 9.25E-17 | 3.28 |
| KRT83 | 3.86E-08 | 3.28 |
| VGLL1 | 2.24E-10 | 3.28 |
| TK1 | 6.27E-34 | 3.28 |
| WDR62 | 5.11E-55 | 3.27 |
| WDR87 | 3.60E-11 | 3.27 |
| DOCK3 | 6.94E-17 | 3.27 |
| DQX1 | 2.34E-16 | 3.27 |
| LINC03044 | 1.50E-26 | 3.26 |
| PCSK1N | 4.35E-11 | 3.26 |
| ACTL8 | 1.94E-09 | 3.26 |
| FCRL3 | 3.84E-09 | 3.26 |
| GUCY1B2 | 1.89E-11 | 3.25 |
| NCCRP1 | 5.85E-10 | 3.25 |
| IGHG2 | 5.19E-09 | 3.25 |
| SPAG5 | 6.30E-43 | 3.24 |
| LINC01567 | 7.68E-09 | 3.23 |
| ZIC4 | 3.69E-14 | 3.23 |
| IGHG1 | 1.19E-08 | 3.23 |
| NCAPH | 1.07E-36 | 3.23 |
| GPC2 | 7.14E-27 | 3.23 |
| ASF1B | 4.26E-35 | 3.23 |
| CLEC18C | 8.73E-13 | 3.22 |
| GNG13 | 2.30E-22 | 3.22 |
| MIR4321 | 7.60E-11 | 3.22 |
| CARMIL2 | 3.39E-20 | 3.22 |
| PTPRN | 1.79E-15 | 3.22 |
| CCNE1 | 2.40E-19 | 3.22 |
| COL19A1 | 1.00E-07 | 3.21 |
| OGDHL | 3.15E-10 | 3.21 |
| TINCR | 9.46E-13 | 3.21 |
| CCDC168 | 7.40E-21 | 3.20 |
| CHRNA6 | 6.24E-16 | 3.19 |
| H2BC8 | 1.35E-15 | 3.19 |
| CCR8 | 6.59E-24 | 3.19 |
| ART3 | 6.95E-08 | 3.19 |
| CDKN2B-AS1 | 3.23E-21 | 3.19 |
| ABCC5-AS1 | 7.76E-12 | 3.19 |
| WT1-AS | 7.02E-09 | 3.18 |
| CD22 | 5.27E-08 | 3.18 |
| UPK1B | 3.70E-11 | 3.18 |
| RTKN2 | 4.43E-35 | 3.17 |
| KIF23 | 1.05E-32 | 3.17 |
| NOL4 | 5.61E-09 | 3.17 |
| LINC01634 | 1.08E-07 | 3.17 |
| KLK4 | 1.74E-09 | 3.17 |
| GJB3 | 7.36E-10 | 3.17 |
| SLC22A31 | 1.51E-13 | 3.17 |
| ZNF670-ZNF695 | 5.87E-19 | 3.16 |
| EZH2 | 1.73E-59 | 3.15 |
| PPFIA4 | 2.44E-22 | 3.15 |
| TRIP13 | 2.07E-26 | 3.15 |
| FBN2 | 1.20E-10 | 3.14 |
| H1-4 | 1.98E-14 | 3.14 |
| ADAMTS16-DT | 9.80E-08 | 3.13 |
| ZNF560 | 1.16E-09 | 3.13 |
| TTC24 | 7.41E-10 | 3.13 |
| STXBP5L | 2.64E-13 | 3.13 |
| SCAT1 | 5.92E-19 | 3.12 |
| AIM2 | 8.40E-13 | 3.12 |
| PRC1-AS1 | 1.49E-33 | 3.12 |
| CDC25C | 4.21E-37 | 3.11 |
| FOXH1 | 1.44E-26 | 3.11 |
| RIPPLY3 | 9.26E-14 | 3.11 |
| RAET1L | 1.43E-10 | 3.11 |
| PRC1 | 6.52E-33 | 3.11 |
| SCX | 1.35E-18 | 3.11 |
| CDKN3 | 2.31E-33 | 3.10 |
| FCRL5 | 2.06E-08 | 3.10 |
| LINC01800 | 3.13E-12 | 3.10 |
| HOXC11 | 3.50E-15 | 3.10 |
| ATP2A1-AS1 | 1.59E-26 | 3.10 |
| CAMK2N2 | 1.42E-14 | 3.10 |
| FAM30A | 5.74E-08 | 3.10 |
| CENPM | 1.10E-39 | 3.10 |
| KCNMB2-AS1 | 2.30E-10 | 3.09 |
| LOC102724560 | 1.22E-18 | 3.09 |
| AMH | 3.89E-10 | 3.09 |
| DISC1-IT1 | 2.60E-12 | 3.09 |
| ERVV-1 | 5.24E-11 | 3.08 |
| INHBA | 1.63E-15 | 3.08 |
| LOC101059954 | 1.16E-12 | 3.08 |
| OR6V1 | 1.13E-11 | 3.08 |
| PNMA5 | 2.66E-06 | 3.08 |
| ACTL6B | 2.36E-12 | 3.08 |
| ELOA3P | 7.10E-15 | 3.07 |
| CAMKV | 8.58E-09 | 3.07 |
| GPRIN1 | 5.09E-16 | 3.07 |
| TAFA3 | 8.90E-13 | 3.06 |
| NCAPG | 5.82E-42 | 3.06 |
| FOXP4-AS1 | 2.76E-23 | 3.06 |
| H3C14 | 8.95E-16 | 3.06 |
| H3C15 | 2.03E-15 | 3.06 |
| CLEC5A | 6.52E-24 | 3.05 |
| IGLV3-9 | 5.14E-06 | 3.05 |
| CBS | 3.70E-18 | 3.05 |
| DNAH8 | 1.91E-11 | 3.05 |
| TEDC2 | 6.53E-45 | 3.05 |
| CENPK | 1.14E-36 | 3.05 |
| LINC00536 | 1.96E-10 | 3.04 |
| KRTAP5-1 | 2.19E-14 | 3.04 |
| ELOA3BP | 7.23E-16 | 3.04 |
| ZIM2-AS1 | 5.89E-11 | 3.04 |
| PSRC1 | 2.90E-30 | 3.04 |
| BOLL | 1.40E-15 | 3.03 |
| CDHR2 | 2.86E-24 | 3.03 |
| LINC01143 | 8.90E-13 | 3.03 |
| FOXD3-AS1 | 1.63E-11 | 3.03 |
| PAX5 | 1.18E-06 | 3.02 |
| HMGA2 | 1.01E-13 | 3.02 |
| BMS1P18 | 1.08E-18 | 3.02 |
| SLC12A1 | 1.27E-08 | 3.02 |
| PPP1R9A-AS1 | 1.10E-11 | 3.02 |
| TPRXL | 3.59E-11 | 3.02 |
| BLM | 8.88E-36 | 3.01 |
| KISS1 | 4.37E-12 | 3.01 |
| KIRREL2 | 2.96E-10 | 3.01 |
| SCARNA18B | 2.11E-14 | 3.01 |
| QRFPR | 5.02E-07 | 3.00 |
| SNORA11E | 1.78E-14 | 3.00 |
| TBC1D27P | 9.89E-09 | 3.00 |
| CPNE7 | 1.14E-11 | 3.00 |
| PITX1 | 1.56E-14 | 3.00 |
| ATAD3C | 1.17E-15 | 3.00 |
| PERM1 | 2.66E-18 | 3.00 |
| ZFR2 | 1.45E-08 | 2.99 |
| LINC02968 | 3.28E-16 | 2.99 |
| KIF11 | 1.06E-34 | 2.99 |
| IL17REL | 1.05E-13 | 2.98 |
| RAET1K | 5.48E-16 | 2.98 |
| COL9A1 | 3.58E-07 | 2.98 |
| MUC5B | 3.64E-11 | 2.98 |
| LOC101929614 | 6.84E-08 | 2.98 |
| ELK2AP | 2.23E-07 | 2.98 |
| NXPH4 | 1.89E-11 | 2.98 |
| BMS1P17 | 2.36E-18 | 2.98 |
| SNORA80A | 1.57E-14 | 2.97 |
| BECN2 | 6.83E-09 | 2.97 |
| SLC11A1 | 7.51E-19 | 2.97 |
| LOC100335030 | 4.05E-19 | 2.97 |
| STAP1 | 1.44E-09 | 2.96 |
| MIR7112 | 1.99E-14 | 2.96 |
| CCDC33 | 8.93E-08 | 2.96 |
| TNFRSF13B | 3.37E-07 | 2.96 |
| CACNA1E | 3.42E-14 | 2.96 |
| TNNT2 | 4.02E-13 | 2.96 |
| PNLDC1 | 1.56E-06 | 2.96 |
| TLX2 | 3.85E-10 | 2.96 |
| FOXF2-DT | 1.53E-10 | 2.96 |
| CIP2A | 7.69E-42 | 2.96 |
| HOTAIR | 4.38E-14 | 2.96 |
| ZWINT | 7.58E-29 | 2.95 |
| RMRP | 3.14E-12 | 2.95 |
| GLDC | 1.02E-10 | 2.95 |
| E2F2 | 2.44E-19 | 2.95 |
| CRYBG2 | 5.72E-19 | 2.94 |
| TNNI3 | 9.67E-08 | 2.94 |
| ADAMDEC1 | 2.89E-10 | 2.94 |
| TACC3 | 1.38E-38 | 2.94 |
| LINC02811 | 2.00E-17 | 2.94 |
| MIOX | 3.45E-27 | 2.93 |
| CDIPTOSP | 8.44E-12 | 2.93 |
| ARMH1 | 2.82E-16 | 2.93 |
| LOC112268238 | 7.59E-20 | 2.93 |
| MIR4480 | 1.59E-11 | 2.92 |
| SNORA11D | 2.35E-13 | 2.92 |
| BPI | 9.70E-11 | 2.92 |
| CCL25 | 1.44E-10 | 2.92 |
| ODAM | 3.75E-04 | 2.92 |
| S100A2 | 2.91E-09 | 2.92 |
| LINC02408 | 2.85E-14 | 2.92 |
| ISG15 | 3.48E-18 | 2.92 |
| RCOR2 | 6.31E-22 | 2.91 |
| PAQR6 | 5.34E-20 | 2.91 |
| CDCA5 | 1.47E-29 | 2.91 |
| HOXA11-AS | 1.36E-07 | 2.91 |
| CHGA | 2.75E-06 | 2.91 |
| GPR137C | 1.96E-15 | 2.91 |
| C10orf88B | 3.68E-37 | 2.91 |
| TNFRSF9 | 3.81E-16 | 2.91 |
| RAD54L | 6.80E-47 | 2.91 |
| UHRF1 | 1.02E-29 | 2.90 |
| PCBP3-AS1 | 1.81E-08 | 2.90 |
| LINC02043 | 1.99E-13 | 2.90 |
| CLDN14 | 2.86E-17 | 2.89 |
| WTAPP1 | 1.43E-10 | 2.89 |
| NKAIN4 | 2.48E-08 | 2.89 |
| CXCR2P1 | 2.18E-09 | 2.89 |
| NEK2-DT | 3.33E-12 | 2.89 |
| MUC19 | 9.24E-07 | 2.89 |
| CPLX3 | 5.94E-08 | 2.88 |
| LINC01224 | 1.46E-07 | 2.88 |
| E2F5-DT | 3.08E-12 | 2.88 |
| CNTNAP2 | 2.03E-07 | 2.87 |
| LINC01694 | 3.07E-09 | 2.87 |
| ERMN | 8.43E-18 | 2.87 |
| KIF18A | 1.34E-24 | 2.86 |
| SNORD88B | 3.36E-26 | 2.86 |
| SMPD5 | 1.68E-13 | 2.86 |
| TMPRSS4 | 2.27E-11 | 2.86 |
| TONSL | 3.55E-28 | 2.86 |
| ASAP1-IT1 | 2.33E-17 | 2.86 |
| DSG1 | 1.73E-04 | 2.86 |
| ARL9 | 1.69E-17 | 2.86 |
| ABCA13 | 7.45E-09 | 2.85 |
| LRP8 | 2.70E-23 | 2.85 |
| GAL3ST2 | 6.82E-09 | 2.84 |
| CENPI | 2.04E-36 | 2.84 |
| CCNB2 | 1.23E-29 | 2.84 |
| GINS1 | 5.69E-28 | 2.84 |
| FRGCA | 2.21E-11 | 2.83 |
| CBX2 | 5.65E-18 | 2.83 |
| SHCBP1 | 7.51E-28 | 2.83 |
| ST8SIA6-AS1 | 3.37E-09 | 2.83 |
| MTFR2 | 7.60E-30 | 2.83 |
| LINC02872 | 4.47E-13 | 2.82 |
| LINC02870 | 9.95E-11 | 2.82 |
| SNORD99 | 6.29E-17 | 2.82 |
| MIR210HG | 4.02E-19 | 2.82 |
| H2AC11 | 3.04E-14 | 2.81 |
| DNAJC5B | 1.86E-11 | 2.81 |
| C21orf58 | 5.36E-34 | 2.81 |
| COMP | 1.43E-09 | 2.81 |
| XKR9 | 2.78E-09 | 2.81 |
| RXFP1 | 2.62E-11 | 2.81 |
| GAL | 7.34E-09 | 2.80 |
| SNORD152 | 2.35E-10 | 2.80 |
| SPATC1 | 3.44E-10 | 2.80 |
| FOXO6-AS1 | 6.19E-14 | 2.80 |
| FNDC9 | 2.15E-10 | 2.80 |
| HTR3A | 4.29E-06 | 2.80 |
| GCGR | 4.38E-07 | 2.80 |
| PATE3 | 6.86E-10 | 2.79 |
| PDCD1 | 5.99E-13 | 2.79 |
| ARHGAP11A | 1.20E-36 | 2.79 |
| LOC440700 | 1.13E-15 | 2.79 |
| TMEM249 | 5.21E-34 | 2.79 |
| RSPO4 | 1.67E-09 | 2.79 |
| PRG2 | 2.77E-10 | 2.79 |
| CRABP1 | 5.83E-07 | 2.79 |
| MROH6 | 3.41E-15 | 2.79 |
| UBE2S | 8.13E-31 | 2.79 |
| MIR3680-2 | 3.26E-16 | 2.78 |
| LINC02100 | 3.04E-14 | 2.78 |
| LINC02367 | 5.56E-16 | 2.78 |
| DANT1 | 5.03E-11 | 2.78 |
| SPOCD1 | 6.16E-14 | 2.78 |
| KIFC2 | 1.02E-25 | 2.78 |
| ZNF571-AS1 | 7.27E-15 | 2.78 |
| STMN1 | 2.56E-28 | 2.78 |
| FIRRE | 9.35E-09 | 2.78 |
| SLURP2 | 1.91E-08 | 2.78 |
| HAGHL | 1.81E-21 | 2.78 |
| WFDC21P | 3.91E-19 | 2.77 |
| MGAT4EP | 3.07E-15 | 2.77 |
| IGFBPL1 | 5.19E-13 | 2.77 |
| LINC00303 | 4.17E-09 | 2.77 |
| STIL | 9.84E-32 | 2.77 |
| MIR4284 | 3.63E-15 | 2.77 |
| ATAD2 | 1.45E-37 | 2.76 |
| MORN3 | 3.02E-15 | 2.76 |
| CARMAL | 1.10E-06 | 2.76 |
| PMFBP1 | 9.71E-16 | 2.76 |
| UMODL1 | 4.91E-11 | 2.76 |
| MIR378I | 1.21E-09 | 2.75 |
| FAM157C | 1.41E-15 | 2.75 |
| IGSF23 | 1.61E-13 | 2.75 |
| DLGAP1-AS5 | 9.79E-08 | 2.75 |
| TMEM190 | 5.30E-15 | 2.75 |
| CCNB1 | 4.47E-26 | 2.74 |
| SMCO2 | 6.80E-13 | 2.74 |
| LINC01108 | 3.92E-11 | 2.74 |
| VSIG1 | 1.32E-12 | 2.74 |
| TONSL-AS1 | 1.90E-26 | 2.74 |
| RHBDL1 | 1.17E-14 | 2.74 |
| H4C8 | 2.71E-11 | 2.73 |
| IL21R | 1.99E-14 | 2.73 |
| LOC106780825 | 1.30E-10 | 2.73 |
| MAP4K1-AS1 | 6.72E-12 | 2.73 |
| C21orf91-OT1 | 1.61E-09 | 2.72 |
| DTL | 1.05E-23 | 2.72 |
| TMEM145 | 1.70E-10 | 2.72 |
| MMP2-AS1 | 1.65E-09 | 2.72 |
| COL6A4P2 | 8.75E-14 | 2.72 |
| MMP9 | 6.77E-09 | 2.72 |
| E2F7 | 4.68E-22 | 2.72 |
| LOC105375800 | 1.37E-13 | 2.71 |
| KCNH2 | 4.23E-07 | 2.71 |
| SLC5A8 | 6.29E-05 | 2.71 |
| CCDC144CP | 3.78E-15 | 2.71 |
| APOBEC3B-AS1 | 1.24E-10 | 2.71 |
| ERC2 | 8.50E-10 | 2.71 |
| RPPH1 | 4.17E-10 | 2.70 |
| UTS2 | 1.80E-11 | 2.70 |
| CCNA2 | 2.23E-26 | 2.70 |
| SLC6A11 | 8.97E-12 | 2.70 |
| BRIP1 | 7.01E-27 | 2.70 |
| LAMP3 | 4.95E-11 | 2.70 |
| CDT1 | 1.43E-22 | 2.70 |
| MAP7D2 | 1.82E-09 | 2.70 |
| TMEM158 | 5.58E-12 | 2.70 |
| MIR3170 | 1.24E-11 | 2.70 |
| CHRNG | 1.29E-19 | 2.70 |
| UROC1 | 1.51E-07 | 2.69 |
| LINC00486 | 4.47E-13 | 2.69 |
| LOC100288637 | 2.28E-28 | 2.69 |
| LINC01711 | 8.51E-10 | 2.68 |
| SMC4 | 1.55E-37 | 2.68 |
| MIR877 | 3.84E-14 | 2.68 |
| SH3GL3 | 1.33E-07 | 2.68 |
| SPINK1 | 9.06E-07 | 2.68 |
| CTXN1 | 1.18E-16 | 2.68 |
| TNFRSF13C | 1.62E-12 | 2.68 |
| INGX | 2.41E-14 | 2.67 |
| ACSL6 | 7.40E-09 | 2.67 |
| LINC00618 | 1.24E-12 | 2.67 |
| RMI2 | 3.41E-24 | 2.67 |
| MNX1-AS1 | 1.17E-06 | 2.67 |
| RNA28SN5 | 7.41E-15 | 2.67 |
| NKX1-2 | 2.42E-08 | 2.67 |
| MCOLN2 | 2.12E-13 | 2.66 |
| BIRC7 | 1.88E-09 | 2.66 |
| ARHGEF26-AS1 | 9.47E-15 | 2.66 |
| CA1 | 1.17E-08 | 2.66 |
| MAP4K1 | 1.22E-11 | 2.66 |
| LPO | 2.07E-07 | 2.66 |
| DNAH14 | 3.87E-26 | 2.66 |
| RASGRF1 | 1.87E-13 | 2.66 |
| GINS4 | 1.56E-35 | 2.66 |
| SNORD60 | 5.36E-16 | 2.66 |
| E2F1 | 7.22E-30 | 2.66 |
| GABRD | 1.32E-17 | 2.66 |
| JPT1 | 2.08E-27 | 2.66 |
| L3MBTL4-AS1 | 1.65E-10 | 2.66 |
| ANKRD36 | 7.55E-20 | 2.66 |
| RHEX | 2.02E-05 | 2.65 |
| JAKMIP1 | 1.88E-12 | 2.65 |
| MFAP2 | 5.91E-16 | 2.65 |
| CCDC78 | 8.33E-19 | 2.64 |
| CCNP | 1.39E-10 | 2.64 |
| NFYCP2 | 3.35E-07 | 2.64 |
| H2BC6 | 3.23E-21 | 2.64 |
| FAM72C | 8.40E-24 | 2.64 |
| SYT5 | 5.22E-08 | 2.64 |
| ROS1 | 6.22E-09 | 2.64 |
| DRAXIN | 2.04E-10 | 2.64 |
| DLX4 | 5.31E-11 | 2.64 |
| ZBP1 | 4.97E-13 | 2.64 |
| DMBX1 | 5.46E-09 | 2.64 |
| BRINP3 | 2.11E-04 | 2.64 |
| CXCL9 | 9.36E-09 | 2.64 |
| FAM131C | 1.25E-08 | 2.64 |
| PCAT6 | 2.55E-32 | 2.64 |
| GLRA2 | 9.60E-09 | 2.63 |
| MIR924HG | 2.62E-14 | 2.63 |
| CCNYL2 | 5.99E-07 | 2.63 |
| ZNF66 | 3.61E-17 | 2.63 |
| LINC02641 | 6.72E-11 | 2.62 |
| PCSK9 | 1.24E-08 | 2.62 |
| ODAD2P1 | 1.87E-06 | 2.62 |
| KCNK9 | 1.14E-10 | 2.62 |
| HGFAC | 4.35E-11 | 2.61 |
| LOC408186 | 5.19E-15 | 2.61 |
| TICRR | 4.29E-24 | 2.61 |
| CLEC17A | 8.73E-06 | 2.61 |
| AKR1C6P | 1.33E-11 | 2.61 |
| OR2H2 | 1.17E-11 | 2.61 |
| CDCA7 | 1.60E-21 | 2.60 |
| DSG3 | 3.36E-05 | 2.60 |
| RGS20 | 7.38E-10 | 2.60 |
| NHLH1 | 1.26E-18 | 2.60 |
| DDX11-AS1 | 1.57E-17 | 2.60 |
| H2BW4P | 6.86E-11 | 2.60 |
| FAM72D | 2.76E-25 | 2.60 |
| MYT1 | 2.40E-07 | 2.60 |
| CTSV | 2.38E-08 | 2.59 |
| RNA28SN4 | 3.86E-15 | 2.59 |
| HSP90AB4P | 2.52E-11 | 2.59 |
| CRLF1 | 5.27E-06 | 2.59 |
| DEUP1 | 7.36E-17 | 2.59 |
| IFI6 | 1.29E-10 | 2.59 |
| EML6-AS1 | 3.99E-11 | 2.59 |
| ESM1 | 7.02E-12 | 2.58 |
| LMNB1 | 6.38E-20 | 2.58 |
| SYNGR3 | 1.04E-19 | 2.58 |
| PPP1R3B-DT | 8.65E-11 | 2.58 |
| CXCL10 | 3.88E-08 | 2.58 |
| H2BC11 | 4.07E-15 | 2.58 |
| RNR1 | 4.09E-11 | 2.58 |
| CCNE2 | 1.15E-30 | 2.57 |
| SNORD38B | 4.70E-20 | 2.57 |
| HTRA4 | 5.91E-14 | 2.57 |
| TRIM59 | 9.68E-27 | 2.57 |
| TAF7L | 9.12E-15 | 2.57 |
| IL21R-AS1 | 1.92E-14 | 2.57 |
| CHRNA1 | 1.23E-10 | 2.57 |
| CAVIN4 | 4.40E-21 | 2.56 |
| UNC5B-AS1 | 4.69E-09 | 2.56 |
| OVOL3 | 3.36E-15 | 2.56 |
| DRC7 | 4.04E-08 | 2.56 |
| NIBAN3 | 3.36E-10 | 2.56 |
| ANKRD34B | 2.88E-07 | 2.56 |
| KIF26B | 2.19E-11 | 2.56 |
| HELLS | 1.86E-24 | 2.55 |
| ANKRD36B | 1.53E-17 | 2.55 |
| KLK1 | 4.84E-09 | 2.55 |
| BICDL1 | 2.22E-28 | 2.55 |
| ORC1 | 4.26E-25 | 2.55 |
| H4C9 | 1.12E-09 | 2.55 |
| CLNK | 1.87E-13 | 2.55 |
| CENPU | 4.07E-24 | 2.55 |
| LINC02878 | 1.77E-13 | 2.54 |
| HRK | 1.41E-07 | 2.54 |
| ANKRD36BP2 | 1.82E-17 | 2.54 |
| ERFE | 1.51E-11 | 2.54 |
| H2BW3P | 4.51E-11 | 2.54 |
| EDDM3A | 1.09E-08 | 2.54 |
| MIR25 | 1.07E-20 | 2.54 |
| RNA45SN4 | 2.16E-15 | 2.54 |
| LINC01588 | 1.23E-13 | 2.54 |
| COL24A1 | 7.60E-12 | 2.54 |
| SNORA72 | 1.91E-23 | 2.54 |
| PLEKHN1 | 1.47E-14 | 2.54 |
| ROCR | 2.03E-06 | 2.54 |
| RPLP0P2 | 2.65E-22 | 2.54 |
| TEKT5 | 6.40E-10 | 2.53 |
| PTTG3P | 3.78E-20 | 2.53 |
| EPIST | 1.33E-04 | 2.53 |
| ANKRD1 | 1.34E-08 | 2.53 |
| LOC112268186 | 5.72E-18 | 2.53 |
| LINC02522 | 2.67E-08 | 2.53 |
| SCARNA2 | 5.22E-10 | 2.53 |
| LTA | 7.62E-21 | 2.53 |
| H2BC5 | 1.40E-15 | 2.52 |
| PLSCR2 | 1.30E-15 | 2.52 |
| CLECL1P | 3.66E-11 | 2.52 |
| PPP1R14C | 9.67E-08 | 2.52 |
| LOC105378936 | 6.54E-14 | 2.52 |
| LOC284412 | 2.16E-13 | 2.52 |
| DKK1 | 1.75E-06 | 2.51 |
| SNORA10 | 2.22E-17 | 2.51 |
| CLPSL2 | 4.16E-06 | 2.51 |
| PDE6B-AS1 | 8.96E-07 | 2.51 |
| SELL | 3.55E-07 | 2.51 |
| SNHG31 | 2.37E-15 | 2.50 |
| SHANK2-AS3 | 4.96E-07 | 2.50 |
| CREB3L2-AS1 | 1.95E-17 | 2.50 |
| SNORD157 | 1.13E-12 | 2.50 |
| KCNJ10 | 1.21E-16 | 2.50 |
| CYP2W1 | 3.55E-10 | 2.50 |
| SNTG2-AS1 | 2.47E-05 | 2.50 |
| LINC02894 | 2.57E-15 | 2.50 |
| COL11A2 | 1.56E-11 | 2.50 |
| LEFTY1 | 1.65E-07 | 2.49 |
| GRIN2D | 1.19E-13 | 2.49 |
| LIX1-AS1 | 6.57E-09 | 2.49 |
| ORM2 | 5.67E-06 | 2.49 |
| LINC03016 | 1.28E-09 | 2.49 |
| MMP12 | 4.76E-09 | 2.49 |
| AFAP1-AS1 | 1.23E-08 | 2.48 |
| RNA28SN1 | 8.17E-14 | 2.48 |
| MTRNR2L2 | 3.66E-11 | 2.48 |
| CEP152 | 4.86E-20 | 2.48 |
| ZNF80 | 8.25E-11 | 2.48 |
| MIR155HG | 1.11E-13 | 2.48 |
| LINC01357 | 1.71E-09 | 2.48 |
| FTCD | 2.00E-11 | 2.47 |
| ZNF124 | 2.34E-21 | 2.47 |
| GRK1 | 4.75E-09 | 2.47 |
| IL12RB2 | 1.21E-10 | 2.47 |
| PROC | 8.61E-17 | 2.47 |
| TMEM151A | 2.86E-07 | 2.47 |
| RHPN1 | 9.79E-19 | 2.47 |
| SRD5A2 | 7.41E-09 | 2.47 |
| BRCA2 | 2.54E-30 | 2.47 |
| B4GALNT3 | 2.53E-12 | 2.46 |
| RNA28SN3 | 2.84E-14 | 2.46 |
| LINC02538 | 9.42E-07 | 2.46 |
| STAM-DT | 3.08E-18 | 2.46 |
| RGR | 4.66E-06 | 2.46 |
| RNA45SN5 | 2.37E-15 | 2.46 |
| CDC6 | 9.49E-22 | 2.46 |
| VXN | 5.27E-10 | 2.46 |
| C8G | 6.14E-16 | 2.46 |
| CIT | 6.19E-29 | 2.46 |
| RNA28SN2 | 4.46E-14 | 2.46 |
| PGBD5 | 2.00E-07 | 2.46 |
| LINC02668 | 3.05E-06 | 2.46 |
| SGO2 | 1.55E-37 | 2.45 |
| GOLGA6L17P | 8.50E-11 | 2.45 |
| DAND5 | 6.40E-15 | 2.45 |
| SULT4A1 | 1.19E-07 | 2.45 |
| LINC00964 | 2.20E-06 | 2.45 |
| RANBP17 | 4.84E-25 | 2.45 |
| LINC00482 | 3.64E-09 | 2.45 |
| B4GALNT4 | 3.41E-15 | 2.45 |
| HMGB3 | 7.96E-22 | 2.45 |
| RABGAP1L-AS1 | 3.72E-13 | 2.45 |
| MIR6807 | 1.40E-16 | 2.44 |
| ZYG11A | 5.49E-11 | 2.44 |
| WT1 | 4.50E-06 | 2.44 |
| MIXL1 | 3.79E-08 | 2.44 |
| NOXO1 | 4.97E-10 | 2.44 |
| F12 | 4.45E-15 | 2.43 |
| PPEF1 | 2.36E-10 | 2.43 |
| CDC25A | 1.02E-20 | 2.43 |
| TRAJ31 | 3.27E-08 | 2.43 |
| EVPL | 2.09E-14 | 2.43 |
| SBK1 | 2.01E-14 | 2.43 |
| ADM2 | 1.54E-19 | 2.43 |
| ELAVL4 | 3.02E-08 | 2.43 |
| C3orf22 | 4.71E-07 | 2.42 |
| RCAN3AS | 3.58E-13 | 2.42 |
| MIR7110 | 4.71E-15 | 2.42 |
| SHC4 | 6.66E-07 | 2.42 |
| KRTAP5-AS1 | 1.28E-08 | 2.42 |
| CCR7 | 4.74E-08 | 2.42 |
| SCARNA7 | 1.09E-12 | 2.42 |
| PGPEP1L | 2.02E-08 | 2.42 |
| MYH16 | 1.65E-14 | 2.42 |
| MDGA2 | 2.76E-08 | 2.41 |
| GBP5 | 1.31E-11 | 2.41 |
| GPR22 | 2.57E-15 | 2.41 |
| CPLX2 | 5.93E-11 | 2.41 |
| MIR4516 | 7.95E-10 | 2.41 |
| PLAAT1 | 7.18E-11 | 2.41 |
| DGCR5 | 1.01E-10 | 2.41 |
| MMP10 | 2.67E-06 | 2.41 |
| DGCR9 | 3.49E-10 | 2.41 |
| SRPK3 | 1.80E-09 | 2.41 |
| RPS15AP10 | 1.45E-17 | 2.41 |
| UNC80 | 2.32E-06 | 2.41 |
| LOC105375737 | 3.14E-15 | 2.41 |
| RPA4 | 2.83E-10 | 2.40 |
| LINC02544 | 2.44E-12 | 2.40 |
| TMEM92 | 1.18E-08 | 2.40 |
| PLEKHG4B | 7.55E-12 | 2.40 |
| SAPCD2 | 2.77E-16 | 2.40 |
| DGCR10 | 6.92E-08 | 2.40 |
| SLC7A5 | 1.79E-16 | 2.40 |
| H4C12 | 1.47E-14 | 2.40 |
| LINC00954 | 2.67E-11 | 2.40 |
| IGLV3-21 | 9.49E-05 | 2.40 |
| RNA45SN3 | 1.05E-14 | 2.40 |
| ZBED2 | 3.42E-09 | 2.40 |
| PNPLA3 | 6.81E-08 | 2.39 |
| CHTF18 | 1.20E-24 | 2.39 |
| RASD2 | 3.60E-08 | 2.39 |
| MIR3917 | 3.93E-15 | 2.39 |
| KRTAP5-9 | 1.45E-13 | 2.39 |
| LOC105375023 | 7.08E-12 | 2.39 |
| LINC01012 | 1.82E-17 | 2.39 |
| RNA45SN2 | 1.29E-14 | 2.39 |
| ANKRD22 | 3.25E-10 | 2.39 |
| SCRG1 | 4.61E-09 | 2.39 |
| OIP5 | 4.58E-30 | 2.39 |
| KBTBD12 | 1.15E-06 | 2.39 |
| MIAT | 5.85E-16 | 2.39 |
| IGLV3-27 | 1.97E-03 | 2.38 |
| ERN2 | 2.90E-14 | 2.38 |
| ZNF730 | 1.38E-11 | 2.38 |
| DDIAS | 2.51E-20 | 2.37 |
| SCAT2 | 4.36E-21 | 2.37 |
| SNORD88A | 3.51E-13 | 2.37 |
| IGSF9 | 1.55E-14 | 2.37 |
| NALF1-IT1 | 1.43E-06 | 2.37 |
| TEX45 | 3.38E-10 | 2.37 |
| LINC01362 | 6.04E-10 | 2.37 |
| H4C3 | 1.83E-23 | 2.37 |
| MMP7 | 8.49E-06 | 2.37 |
| MIR3651 | 3.42E-14 | 2.36 |
| RNR2 | 4.29E-11 | 2.36 |
| SNORA109 | 7.54E-10 | 2.36 |
| IGHV4-4 | 5.79E-04 | 2.36 |
| KPNA7 | 4.56E-17 | 2.36 |
| PSORS1C1 | 8.06E-11 | 2.36 |
| DDN | 6.44E-09 | 2.36 |
| LINC03025 | 1.92E-10 | 2.36 |
| CDHR1 | 6.01E-08 | 2.36 |
| PRSS27 | 3.28E-16 | 2.36 |
| ABRACL | 2.88E-18 | 2.36 |
| UGT2B7 | 1.40E-04 | 2.36 |
| LINC02576 | 4.09E-14 | 2.36 |
| GS1-279B7.1 | 2.11E-12 | 2.35 |
| KRT87P | 2.70E-06 | 2.35 |
| CTNS-AS1 | 7.29E-12 | 2.35 |
| IGLV3-16 | 3.39E-04 | 2.35 |
| LOC105376287 | 1.05E-16 | 2.35 |
| FAM83A | 2.45E-05 | 2.35 |
| ZNF208 | 1.64E-06 | 2.34 |
| GOLGA6L3P | 6.55E-09 | 2.34 |
| CLIC3 | 2.67E-11 | 2.34 |
| IGHV1-18 | 1.80E-04 | 2.34 |
| CRHR1 | 3.19E-05 | 2.34 |
| MTRNR2L1 | 1.35E-06 | 2.34 |
| WDR97 | 1.10E-12 | 2.34 |
| INSM1 | 4.79E-05 | 2.34 |
| C5orf46 | 1.25E-06 | 2.33 |
| LCN2 | 3.88E-05 | 2.33 |
| DNASE1L2 | 5.21E-13 | 2.33 |
| APOBEC3B | 1.12E-08 | 2.33 |
| EPGN | 1.07E-07 | 2.33 |
| G2E3-AS1 | 5.03E-12 | 2.33 |
| FAM72A | 1.18E-20 | 2.33 |
| LRRCC1 | 8.80E-16 | 2.33 |
| TGFBR3L | 4.36E-12 | 2.33 |
| CARD17P | 1.15E-09 | 2.33 |
| SNORA73A | 1.78E-14 | 2.33 |
| HPDL | 1.81E-17 | 2.33 |
| CRPPA-AS1 | 3.68E-08 | 2.33 |
| LRRC14B | 1.92E-07 | 2.33 |
| UFL1-AS1 | 2.82E-16 | 2.32 |
| LAG3 | 1.43E-20 | 2.32 |
| HOXA11 | 3.95E-05 | 2.32 |
| SHISAL2A | 2.77E-11 | 2.32 |
| LYG1 | 2.08E-18 | 2.32 |
| GZMB | 2.18E-09 | 2.32 |
| TREML2 | 1.06E-05 | 2.32 |
| CELSR3 | 2.94E-12 | 2.32 |
| MTBP | 1.38E-24 | 2.32 |
| SQLE | 5.10E-15 | 2.31 |
| BCL2A1 | 5.10E-16 | 2.31 |
| MIR3680-1 | 4.69E-15 | 2.31 |
| ATAD5 | 9.64E-28 | 2.31 |
| YJEFN3 | 1.07E-13 | 2.31 |
| LINC01133 | 5.45E-07 | 2.31 |
| TMEM92-AS1 | 7.02E-11 | 2.30 |
| SNORA68B | 3.68E-12 | 2.30 |
| KLHDC7B | 4.06E-07 | 2.30 |
| GNB3 | 1.28E-14 | 2.30 |
| LINC02806 | 1.49E-07 | 2.30 |
| TMEM274P | 1.05E-12 | 2.30 |
| NHSL1-AS1 | 2.00E-19 | 2.30 |
| GAS2L3 | 4.86E-22 | 2.30 |
| SPACDR | 2.79E-12 | 2.29 |
| SPACA4 | 1.73E-07 | 2.29 |
| H4C11 | 8.07E-14 | 2.29 |
| LOC728715 | 1.74E-12 | 2.29 |
| MYO3A | 1.92E-05 | 2.29 |
| TBC1D3JP | 2.10E-12 | 2.29 |
| AURKAP1 | 1.72E-23 | 2.29 |
| DUXAP9 | 3.04E-12 | 2.29 |
| FUT7 | 2.05E-11 | 2.29 |
| BGN | 1.69E-09 | 2.29 |
| LOC107985297 | 4.12E-20 | 2.29 |
| DNAH12 | 8.54E-09 | 2.29 |
| JPH3 | 1.58E-10 | 2.28 |
| MMP20 | 3.12E-05 | 2.28 |
| RNFT2 | 8.11E-18 | 2.28 |
| SNORA25 | 3.34E-20 | 2.28 |
| ACTG1P25 | 3.88E-16 | 2.28 |
| TNFRSF18 | 1.22E-14 | 2.28 |
| RNA45SN1 | 7.17E-15 | 2.28 |
| SNORA57 | 1.40E-14 | 2.28 |
| DSG1-AS1 | 5.83E-03 | 2.28 |
| PROZ | 5.37E-10 | 2.28 |
| FOXQ1 | 5.07E-06 | 2.28 |
| PAQR4 | 4.48E-22 | 2.28 |
| LOC101409256 | 7.05E-14 | 2.27 |
| SP140 | 3.97E-11 | 2.27 |
| LINC02280 | 6.20E-10 | 2.27 |
| FAM27E4 | 5.23E-08 | 2.27 |
| PPP2R2C | 1.02E-06 | 2.27 |
| SYT14 | 4.24E-05 | 2.27 |
| TMEM179 | 1.90E-04 | 2.27 |
| ITGB2-AS1 | 3.67E-13 | 2.27 |
| MIR200CHG | 1.79E-12 | 2.27 |
| RAD51 | 9.10E-24 | 2.27 |
| SYCP2L | 3.89E-07 | 2.27 |
| ARMC12 | 6.61E-12 | 2.27 |
| LINC01599 | 4.16E-10 | 2.27 |
| GPR18 | 2.74E-16 | 2.27 |
| C6orf52 | 9.54E-16 | 2.27 |
| LINC03034 | 4.44E-09 | 2.27 |
| EAF2 | 2.57E-14 | 2.26 |
| SLC22A20P | 1.30E-12 | 2.26 |
| SLC2A1 | 3.47E-07 | 2.26 |
| H2AC19 | 1.03E-11 | 2.26 |
| POP1 | 1.08E-26 | 2.26 |
| PA2G4P4 | 3.85E-14 | 2.26 |
| UCN2 | 7.66E-07 | 2.26 |
| S100A9 | 2.95E-05 | 2.26 |
| SAPCD1 | 5.94E-18 | 2.26 |
| PSAT1 | 3.33E-12 | 2.26 |
| CAMK2A | 2.07E-10 | 2.26 |
| RHBG | 1.11E-07 | 2.26 |
| LINC01096 | 2.50E-04 | 2.26 |
| RNF113B | 5.61E-12 | 2.26 |
| TSPAN16 | 5.05E-09 | 2.26 |
| LINC03026 | 7.00E-06 | 2.26 |
| CCDC144NL | 3.07E-07 | 2.26 |
| EEF1AKMT4 | 2.99E-15 | 2.26 |
| CCL17 | 4.43E-09 | 2.26 |
| SNORA84 | 9.30E-16 | 2.25 |
| LGR5 | 3.12E-04 | 2.25 |
| MIR648 | 1.27E-09 | 2.25 |
| BCYRN1 | 2.35E-09 | 2.25 |
| CFAP58-DT | 1.20E-11 | 2.25 |
| KCNG3 | 2.51E-08 | 2.25 |
| HSH2D | 3.23E-09 | 2.25 |
| KRT75 | 4.96E-04 | 2.25 |
| RNA5-8SN4 | 1.10E-08 | 2.25 |
| SMYD3-AS1 | 3.02E-12 | 2.25 |
| DNA2 | 2.82E-22 | 2.25 |
| KIF20B | 1.43E-25 | 2.25 |
| MIR6739 | 1.52E-17 | 2.24 |
| LINC01978 | 3.30E-08 | 2.24 |
| KCNQ5 | 1.16E-08 | 2.24 |
| PTPN7 | 3.67E-13 | 2.24 |
| IGLV3-25 | 3.67E-04 | 2.24 |
| H2AC18 | 1.41E-11 | 2.24 |
| LAPTM4A-DT | 1.44E-09 | 2.24 |
| SLC2A1-DT | 4.43E-13 | 2.24 |
| OR13A1 | 3.01E-07 | 2.24 |
| PDE4B-AS1 | 1.38E-04 | 2.24 |
| APLP1 | 8.45E-08 | 2.24 |
| ALPK2 | 8.53E-09 | 2.24 |
| OR7E91P | 6.88E-08 | 2.24 |
| FDPSP2 | 3.92E-19 | 2.24 |
| IGLV1-47 | 1.65E-04 | 2.23 |
| FAUP4 | 4.85E-12 | 2.23 |
| DDX11 | 1.60E-14 | 2.23 |
| TNIP3 | 1.31E-09 | 2.23 |
| SDS | 1.03E-10 | 2.23 |
| ATP2A1 | 1.95E-19 | 2.23 |
| SH2D5 | 4.24E-09 | 2.23 |
| LOC107986874 | 9.14E-10 | 2.23 |
| EXTL1 | 7.54E-05 | 2.23 |
| BOP1 | 1.43E-21 | 2.23 |
| SPDYE2B | 2.50E-15 | 2.23 |
| TSACC | 6.99E-24 | 2.23 |
| MTRNR2L8 | 7.28E-10 | 2.23 |
| SLC28A1 | 1.28E-09 | 2.23 |
| GPAT4-AS1 | 9.81E-23 | 2.22 |
| ASIC3 | 9.81E-13 | 2.22 |
| ST18 | 3.03E-11 | 2.22 |
| MIR181A2HG | 1.48E-12 | 2.22 |
| MYH15 | 1.88E-12 | 2.22 |
| GRM6 | 8.65E-12 | 2.22 |
| PACSIN1 | 1.33E-09 | 2.22 |
| PNOC | 1.88E-05 | 2.22 |
| TEDDM1 | 4.81E-08 | 2.22 |
| TBC1D3P2 | 2.99E-12 | 2.21 |
| SLCO1A2 | 6.75E-04 | 2.21 |
| AQP6 | 1.54E-05 | 2.21 |
| S100A1 | 1.39E-06 | 2.21 |
| PPP1R14B | 7.77E-36 | 2.21 |
| DHDH | 2.10E-14 | 2.21 |
| PLK4 | 2.26E-18 | 2.21 |
| TRIM46 | 7.63E-17 | 2.21 |
| SHISA9 | 5.12E-05 | 2.21 |
| PYCR1 | 3.09E-15 | 2.21 |
| PAFAH1B3 | 1.12E-20 | 2.21 |
| PANX2 | 2.62E-10 | 2.21 |
| SNORD56 | 1.01E-16 | 2.20 |
| KLRG2 | 7.36E-10 | 2.20 |
| CYP2F1 | 1.22E-08 | 2.20 |
| IGLV1-44 | 1.54E-04 | 2.20 |
| GINS2 | 4.50E-14 | 2.20 |
| CD72 | 2.05E-14 | 2.20 |
| PCAT1 | 1.99E-08 | 2.20 |
| ASCL5 | 1.39E-10 | 2.20 |
| MYBPHL | 3.87E-08 | 2.20 |
| CCDC18 | 8.88E-22 | 2.20 |
| BSG-AS1 | 1.68E-11 | 2.20 |
| ERFL | 2.06E-19 | 2.20 |
| CEP295NL | 2.48E-13 | 2.20 |
| LINC03063 | 3.84E-09 | 2.20 |
| MIR10525 | 5.97E-13 | 2.20 |
| DCANP1 | 1.92E-07 | 2.19 |
| MYH9-DT | 2.69E-13 | 2.19 |
| SLC16A10 | 5.80E-12 | 2.19 |
| GPR84 | 3.78E-15 | 2.19 |
| HS3ST6 | 1.50E-04 | 2.19 |
| NDST3 | 1.47E-08 | 2.19 |
| DDX12P | 1.49E-19 | 2.19 |
| LINC00605 | 1.88E-06 | 2.19 |
| BTBD9-AS1 | 8.98E-13 | 2.19 |
| POU5F1 | 6.43E-15 | 2.18 |
| APPAT | 2.78E-17 | 2.18 |
| NECTIN4-AS1 | 3.52E-10 | 2.18 |
| KISS1R | 3.51E-06 | 2.18 |
| TBC1D3 | 9.10E-12 | 2.18 |
| TBC1D26 | 2.20E-07 | 2.18 |
| PLAUR | 3.47E-20 | 2.18 |
| SLC6A15 | 2.44E-04 | 2.18 |
| IGLV9-49 | 1.82E-03 | 2.18 |
| MYBPC2 | 4.96E-08 | 2.18 |
| EMC3-AS1 | 2.48E-14 | 2.18 |
| LIN9 | 1.84E-24 | 2.18 |
| ULBP2 | 1.29E-07 | 2.18 |
| STRA6 | 2.34E-11 | 2.18 |
| H2BC12 | 1.86E-13 | 2.18 |
| CLEC18A | 9.93E-09 | 2.17 |
| IL11 | 8.62E-10 | 2.17 |
| OR10AD1 | 1.57E-08 | 2.17 |
| TAF1D | 3.02E-16 | 2.17 |
| TRPM2-AS | 1.91E-11 | 2.17 |
| KCNMB3 | 2.96E-14 | 2.17 |
| PPIAL4H | 1.73E-11 | 2.17 |
| LY6D | 2.11E-03 | 2.17 |
| RPRML | 5.49E-05 | 2.17 |
| NRIR | 7.73E-09 | 2.17 |
| ESPNP | 4.36E-07 | 2.17 |
| TRAF3IP3 | 1.56E-13 | 2.17 |
| SLCO5A1 | 4.83E-14 | 2.16 |
| HAUS7 | 7.37E-13 | 2.16 |
| SCT | 1.52E-16 | 2.16 |
| LINC00487 | 3.17E-09 | 2.16 |
| IGLV3-10 | 7.43E-04 | 2.16 |
| SLC7A11 | 6.11E-13 | 2.16 |
| H2AC20 | 1.98E-12 | 2.16 |
| CD207 | 4.03E-05 | 2.16 |
| SYT16 | 3.11E-07 | 2.16 |
| IGHV4-34 | 3.56E-04 | 2.16 |
| ADCK5 | 5.28E-26 | 2.16 |
| POLE2 | 1.46E-24 | 2.16 |
| NPAS2-AS1 | 1.51E-08 | 2.16 |
| MAD2L1 | 4.71E-12 | 2.16 |
| RSPH6A | 2.93E-09 | 2.16 |
| ITGAX | 2.05E-12 | 2.15 |
| SNORA33 | 7.60E-16 | 2.15 |
| LINC01977 | 4.17E-11 | 2.15 |
| RNF151 | 2.98E-12 | 2.15 |
| FAF1-AS1 | 3.35E-18 | 2.15 |
| LINC01572 | 3.01E-15 | 2.15 |
| CDC27P9 | 8.11E-09 | 2.15 |
| MMP3 | 1.24E-05 | 2.15 |
| DSCC1 | 1.33E-19 | 2.14 |
| RNA5-8SN3 | 2.39E-08 | 2.14 |
| CAPS | 1.07E-11 | 2.14 |
| FCER2 | 3.71E-03 | 2.14 |
| ZNF451-AS1 | 3.19E-21 | 2.14 |
| TMPRSS11A | 4.66E-07 | 2.14 |
| SNORA58 | 3.30E-12 | 2.14 |
| NUP210 | 8.39E-11 | 2.14 |
| CDH15 | 5.19E-08 | 2.13 |
| CHI3L1 | 5.76E-05 | 2.13 |
| KLK6 | 1.87E-04 | 2.13 |
| RHBDL2 | 2.13E-19 | 2.13 |
| PPP1R27 | 5.69E-09 | 2.13 |
| CRACD | 1.85E-10 | 2.13 |
| ANKRD26P3 | 1.57E-04 | 2.13 |
| TOX-DT | 1.49E-08 | 2.13 |
| CHST6 | 4.88E-08 | 2.13 |
| CDC27P10 | 4.29E-09 | 2.13 |
| ANKRD18B | 1.35E-06 | 2.13 |
| BAIAP2L2 | 2.00E-16 | 2.13 |
| CD37 | 2.38E-09 | 2.13 |
| LIMD2 | 1.97E-20 | 2.13 |
| ABHD11 | 1.12E-13 | 2.13 |
| C12orf56 | 1.24E-06 | 2.13 |
| DBF4 | 1.59E-33 | 2.13 |
| KLK8 | 1.80E-04 | 2.13 |
| CHODL | 1.32E-07 | 2.13 |
| MIR6858 | 3.56E-10 | 2.13 |
| EME1 | 2.97E-23 | 2.13 |
| C5orf34 | 2.17E-13 | 2.12 |
| CYP8B1 | 3.67E-04 | 2.12 |
| OR7E37P | 1.44E-14 | 2.12 |
| SAPCD1-AS1 | 2.21E-16 | 2.12 |
| INAVA | 7.72E-09 | 2.12 |
| NAALADL2-AS2 | 1.97E-07 | 2.12 |
| PKLR | 2.79E-17 | 2.12 |
| RNA18SN5 | 1.99E-15 | 2.12 |
| SCARNA4 | 4.07E-18 | 2.12 |
| EPCAM | 5.23E-09 | 2.12 |
| EEF1AKMT4-ECE2 | 1.20E-12 | 2.12 |
| LINC00861 | 4.94E-07 | 2.12 |
| SNORD4B | 4.71E-17 | 2.12 |
| SPDYE2 | 6.05E-15 | 2.12 |
| SAP25 | 1.16E-13 | 2.11 |
| RNA18SN3 | 9.37E-15 | 2.11 |
| CDH19 | 2.84E-03 | 2.11 |
| IFNG | 2.87E-06 | 2.11 |
| MC1R | 1.72E-14 | 2.11 |
| FOXP3 | 4.01E-15 | 2.11 |
| GAD1 | 7.36E-08 | 2.11 |
| IKZF3 | 1.33E-07 | 2.11 |
| PCAT5 | 1.43E-05 | 2.11 |
| PYDC1 | 5.28E-05 | 2.11 |
| FAM83H-AS1 | 9.95E-11 | 2.11 |
| MIR624 | 1.72E-10 | 2.11 |
| PNCK | 3.59E-11 | 2.11 |
| FANCD2OS | 6.87E-23 | 2.11 |
| PRKCA-AS1 | 1.04E-07 | 2.11 |
| SLC35G5 | 2.89E-10 | 2.11 |
| RNA18SN4 | 4.72E-15 | 2.10 |
| MIR6835 | 1.11E-09 | 2.10 |
| MIR939 | 8.47E-17 | 2.10 |
| RNA18SN2 | 1.68E-14 | 2.10 |
| LINC01869 | 5.01E-08 | 2.10 |
| AMZ1 | 6.65E-17 | 2.10 |
| MCM4 | 7.78E-17 | 2.10 |
| GPRC5D | 4.07E-09 | 2.10 |
| LOC606724 | 2.26E-16 | 2.10 |
| PARPBP | 1.38E-21 | 2.10 |
| SNORA61 | 3.49E-17 | 2.10 |
| CHEK1 | 2.80E-26 | 2.10 |
| RNA18SN1 | 8.36E-15 | 2.10 |
| TBC1D3B | 1.92E-12 | 2.10 |
| MINDY4B | 1.24E-07 | 2.10 |
| UPK2 | 5.19E-11 | 2.09 |
| WNT16 | 1.60E-07 | 2.09 |
| SH2D2A | 1.68E-23 | 2.09 |
| KRT16 | 2.81E-05 | 2.09 |
| SLC34A3 | 8.35E-08 | 2.09 |
| SLC39A4 | 3.62E-14 | 2.09 |
| PIK3CD-AS1 | 2.73E-08 | 2.09 |
| SLC52A1 | 3.03E-05 | 2.09 |
| GGH | 4.21E-13 | 2.09 |
| FBXL6 | 8.07E-26 | 2.09 |
| LOC112543491 | 7.83E-17 | 2.09 |
| SNORD41 | 8.31E-13 | 2.09 |
| LOC105374466 | 3.94E-07 | 2.09 |
| KASH5 | 1.14E-04 | 2.09 |
| TSL | 2.51E-08 | 2.09 |
| GDNF | 1.35E-05 | 2.08 |
| IGHV3-21 | 6.76E-04 | 2.08 |
| RIBC2 | 1.21E-13 | 2.08 |
| LMAN1L | 1.12E-04 | 2.08 |
| SNORD42A | 5.25E-13 | 2.08 |
| C9orf153 | 1.54E-08 | 2.08 |
| DLGAP3 | 2.28E-10 | 2.08 |
| GAPDH-DT | 3.77E-20 | 2.08 |
| RAPSN | 6.38E-08 | 2.08 |
| MTRNR2L9 | 1.02E-08 | 2.08 |
| GSK3B-DT | 1.43E-20 | 2.08 |
| ANO9 | 6.22E-15 | 2.08 |
| ATP11A-AS1 | 1.28E-12 | 2.07 |
| MIR1972-2 | 2.00E-10 | 2.07 |
| PRKX-AS1 | 1.14E-11 | 2.07 |
| EFNA3 | 9.93E-11 | 2.07 |
| RNA5-8SN2 | 4.04E-08 | 2.07 |
| H1-2 | 5.84E-09 | 2.07 |
| PCA3 | 2.10E-05 | 2.07 |
| KRTAP5-2 | 4.22E-07 | 2.07 |
| MIR6836 | 6.53E-12 | 2.07 |
| DBNDD1 | 7.55E-13 | 2.07 |
| UNC13A | 4.82E-11 | 2.07 |
| LOC107986211 | 2.66E-08 | 2.07 |
| LOC112268294 | 3.10E-17 | 2.06 |
| EN2 | 2.16E-04 | 2.06 |
| APOC2 | 1.11E-13 | 2.06 |
| PARP15 | 3.39E-08 | 2.06 |
| LOC112268262 | 2.57E-15 | 2.06 |
| S100A14 | 1.33E-06 | 2.06 |
| SLC20A1-DT | 2.18E-10 | 2.06 |
| SNORD14E | 2.03E-06 | 2.06 |
| SNORA73B | 8.97E-11 | 2.06 |
| SNORD50A | 1.70E-15 | 2.06 |
| WNT10A | 8.48E-07 | 2.06 |
| MIR3648-1 | 2.21E-09 | 2.06 |
| LOC102724621 | 8.20E-09 | 2.06 |
| ANKLE1 | 1.15E-13 | 2.06 |
| LOC107986830 | 5.84E-11 | 2.06 |
| RNA5-8SN1 | 1.16E-07 | 2.05 |
| FAM183A | 8.46E-07 | 2.05 |
| GPR171 | 3.87E-10 | 2.05 |
| SNORD4A | 6.35E-13 | 2.05 |
| CCDC154 | 3.29E-09 | 2.05 |
| LOC107986084 | 1.27E-12 | 2.05 |
| EMSLR | 2.48E-11 | 2.05 |
| RSPH10B2 | 3.37E-11 | 2.05 |
| FBXL19-AS1 | 3.76E-12 | 2.05 |
| NIPAL4-DT | 9.12E-13 | 2.05 |
| SHTN1 | 3.20E-10 | 2.05 |
| GRM4 | 4.73E-07 | 2.05 |
| LINC02901 | 3.97E-11 | 2.05 |
| CHKA-DT | 1.02E-06 | 2.04 |
| CCDC137 | 3.03E-16 | 2.04 |
| UBE2CP5 | 4.46E-11 | 2.04 |
| ZC3H12D | 2.78E-09 | 2.04 |
| LINC00896 | 3.30E-08 | 2.04 |
| KRT4 | 5.29E-03 | 2.04 |
| IGLJ3 | 4.47E-04 | 2.04 |
| HEATR6-DT | 2.37E-16 | 2.04 |
| CBLC | 2.19E-11 | 2.04 |
| HASPIN | 1.37E-15 | 2.04 |
| LINC00930 | 5.87E-05 | 2.04 |
| ICOS | 1.04E-07 | 2.03 |
| VSTM2L | 1.13E-05 | 2.03 |
| SLC6A7 | 3.70E-09 | 2.03 |
| HPN-AS1 | 1.15E-08 | 2.03 |
| EP300-AS1 | 5.09E-11 | 2.03 |
| FANCA | 1.77E-26 | 2.03 |
| KRT6A | 7.38E-04 | 2.03 |
| ERP27 | 2.04E-06 | 2.03 |
| KNTC1 | 2.45E-27 | 2.03 |
| ERAS | 8.24E-10 | 2.03 |
| CCDC167 | 1.41E-18 | 2.03 |
| TIFAB | 1.08E-06 | 2.03 |
| RSPH10B | 1.53E-10 | 2.03 |
| ANKRD36C | 1.07E-10 | 2.03 |
| MUC8 | 2.95E-06 | 2.03 |
| SNCB | 8.28E-06 | 2.02 |
| BOLA2B | 1.82E-33 | 2.02 |
| IQANK1 | 8.63E-08 | 2.02 |
| OPRD1 | 1.80E-07 | 2.02 |
| LOC105377805 | 1.44E-05 | 2.02 |
| TCP10L2 | 1.03E-05 | 2.02 |
| HCN2 | 1.43E-18 | 2.02 |
| CDHR5 | 1.01E-08 | 2.02 |
| HOXC-AS3 | 4.70E-09 | 2.02 |
| C16orf92 | 1.85E-08 | 2.02 |
| APOC4-APOC2 | 1.45E-13 | 2.02 |
| IGHV2-26 | 3.48E-03 | 2.02 |
| CNGB1 | 5.32E-07 | 2.02 |
| H2BC12L | 4.56E-11 | 2.02 |
| LINC01934 | 1.35E-06 | 2.01 |
| AIFM3 | 4.55E-10 | 2.01 |
| TBC1D3C | 4.20E-12 | 2.01 |
| DENND3-AS1 | 5.08E-12 | 2.01 |
| LOC105376353 | 2.56E-05 | 2.01 |
| MIR4786 | 9.52E-12 | 2.01 |
| TARID | 5.88E-05 | 2.01 |
| RBAKDN | 9.93E-08 | 2.01 |
| LINC00926 | 4.95E-07 | 2.01 |
| TMEM123-DT | 4.67E-10 | 2.01 |
| TBC1D3H | 2.57E-11 | 2.01 |
| RNA5-8SN5 | 1.63E-07 | 2.01 |
| MIR320B2 | 9.41E-12 | 2.01 |
| HSD17B6 | 3.81E-15 | 2.01 |
| LOC101928096 | 7.99E-13 | 2.01 |
| TBC1D3G | 5.25E-11 | 2.01 |
| LOC102724334 | 4.29E-11 | 2.01 |
| PXT1 | 4.95E-10 | 2.01 |
| IGLJ2 | 3.32E-04 | 2.01 |
| ZNF69 | 4.25E-11 | 2.01 |
| CD52 | 7.62E-07 | 2.01 |
| BTLA | 8.50E-05 | 2.01 |
| MIR3648-2 | 1.07E-08 | 2.01 |
| MYT1L | 8.88E-06 | 2.01 |
| ADAM8 | 1.16E-18 | 2.00 |
| PANO1 | 2.08E-17 | 2.00 |
| MROH7 | 6.37E-07 | 2.00 |
| SLAMF6 | 2.63E-06 | 2.00 |
| PNPLA1 | 2.37E-11 | 2.00 |
| MIF | 3.04E-22 | 2.00 |
| APOC1 | 1.24E-11 | 2.00 |
| GOLGA6L10 | 4.07E-09 | 2.00 |
| TMEM132A | 7.06E-16 | 2.00 |
| NASP | 2.87E-25 | 1.99 |
| TRNM | 6.81E-06 | 1.99 |
| CASC8 | 1.66E-05 | 1.99 |
| GTSF1 | 7.10E-06 | 1.99 |
| TBC1D3I | 9.02E-12 | 1.99 |
| FBP2 | 2.20E-06 | 1.99 |
| NPIPB5 | 1.63E-14 | 1.99 |
| CXCL11 | 5.10E-05 | 1.99 |
| EIF4EBP1 | 2.09E-14 | 1.99 |
| CD80 | 1.11E-12 | 1.99 |
| ITGB1BP2 | 4.94E-17 | 1.99 |
| MTRNR2L6 | 3.75E-08 | 1.99 |
| DYDC2 | 5.77E-05 | 1.99 |
| SPDYE8 | 5.54E-17 | 1.99 |
| ANKRD44-IT1 | 5.84E-11 | 1.99 |
| SUGCT-AS1 | 2.62E-11 | 1.99 |
| CCNA1 | 1.50E-05 | 1.99 |
| MSL3P1 | 3.88E-15 | 1.99 |
| PRSS21 | 5.37E-03 | 1.98 |
| LINC00887 | 6.06E-07 | 1.98 |
| RAD51AP2 | 6.73E-08 | 1.98 |
| FAM24B | 3.70E-16 | 1.98 |
| CENPL | 1.60E-18 | 1.98 |
| MIR6734 | 4.35E-16 | 1.98 |
| LINC02198 | 2.51E-05 | 1.98 |
| SNORD22 | 1.02E-14 | 1.98 |
| MIR6887 | 3.70E-06 | 1.98 |
| LINC00944 | 1.14E-06 | 1.98 |
| SNHG25 | 6.76E-09 | 1.98 |
| STAC3 | 9.67E-14 | 1.98 |
| FGFR4 | 1.74E-06 | 1.98 |
| MIR6728 | 5.20E-09 | 1.97 |
| TBC1D3F | 2.35E-12 | 1.97 |
| SND1-IT1 | 2.55E-19 | 1.97 |
| HES6 | 9.81E-12 | 1.97 |
| POU2AF1 | 1.66E-04 | 1.97 |
| CCDC138 | 9.67E-17 | 1.97 |
| LOC100130298 | 2.38E-06 | 1.97 |
| SSTR3 | 2.31E-05 | 1.97 |
| TLCD3B | 1.80E-09 | 1.97 |
| SLC12A3 | 2.79E-07 | 1.97 |
| MESP2 | 8.81E-07 | 1.97 |
| CEP128 | 6.35E-16 | 1.97 |
| CCDC179 | 2.04E-07 | 1.97 |
| FBXL13 | 3.30E-07 | 1.97 |
| LINC02602 | 5.31E-06 | 1.97 |
| GFUS | 1.36E-22 | 1.97 |
| ACTN2 | 2.21E-06 | 1.96 |
| ATP2C2-AS1 | 2.23E-08 | 1.96 |
| NPIPB12 | 2.66E-13 | 1.96 |
| KIRREL3 | 7.13E-07 | 1.96 |
| MIR6741 | 2.07E-10 | 1.96 |
| H3-4 | 5.36E-09 | 1.96 |
| DUXAP10 | 1.11E-09 | 1.96 |
| MIR6793 | 6.00E-09 | 1.96 |
| LOC105376678 | 5.33E-13 | 1.96 |
| TBC1D3D | 1.15E-11 | 1.96 |
| KCNAB3 | 4.34E-10 | 1.96 |
| CPNE9 | 9.83E-10 | 1.96 |
| LOC112268277 | 8.00E-15 | 1.96 |
| UBD | 3.17E-05 | 1.96 |
| SPDYE6 | 8.16E-14 | 1.96 |
| APOA1 | 1.31E-05 | 1.96 |
| SCN1A | 1.07E-04 | 1.96 |
| SNORA65 | 1.59E-16 | 1.96 |
| SYT13 | 1.11E-04 | 1.96 |
| GNG4 | 3.56E-04 | 1.96 |
| FANCD2 | 8.83E-22 | 1.96 |
| CHRNA10 | 3.59E-13 | 1.95 |
| PKD2L1 | 3.43E-10 | 1.95 |
| SCO2 | 1.08E-16 | 1.95 |
| GSEC | 3.63E-16 | 1.95 |
| IGLC5 | 4.77E-04 | 1.95 |
| KPNA2 | 3.11E-12 | 1.95 |
| ARRDC5 | 5.03E-11 | 1.95 |
| MIR4435-1 | 1.35E-08 | 1.95 |
| IGLV1-36 | 1.10E-03 | 1.95 |
| MTRNR2L10 | 6.94E-08 | 1.95 |
| CDC27P11 | 9.19E-09 | 1.95 |
| SMKR1 | 1.96E-06 | 1.95 |
| LINC02803 | 4.74E-18 | 1.95 |
| SNORA26 | 3.55E-16 | 1.95 |
| C4orf48 | 6.72E-16 | 1.95 |
| CCDC144BP | 2.40E-09 | 1.95 |
| MIF-AS1 | 6.95E-22 | 1.95 |
| CDC7 | 7.54E-17 | 1.95 |
| LINC00342 | 4.19E-09 | 1.95 |
| KRT9 | 3.42E-04 | 1.95 |
| DUOXA2 | 5.86E-06 | 1.95 |
| SNORD57 | 2.35E-13 | 1.95 |
| IGHV2-70 | 2.85E-03 | 1.94 |
| THOC7-AS1 | 4.92E-09 | 1.94 |
| ERCC6L | 2.17E-21 | 1.94 |
| GRHL3-AS1 | 1.13E-06 | 1.94 |
| SNORA68 | 1.06E-10 | 1.94 |
| RBPJL | 1.68E-07 | 1.94 |
| LOC105374298 | 4.85E-12 | 1.94 |
| COX2 | 1.21E-09 | 1.94 |
| PBX4 | 1.81E-11 | 1.94 |
| C5orf34-AS1 | 5.59E-07 | 1.94 |
| PCDHGB8P | 4.90E-11 | 1.94 |
| TYMSOS | 1.52E-11 | 1.94 |
| SNORD104 | 3.46E-09 | 1.94 |
| KEL | 5.65E-09 | 1.94 |
| SH3GL2 | 3.76E-07 | 1.93 |
| SPDYE13 | 3.30E-17 | 1.93 |
| CD24 | 3.92E-06 | 1.93 |
| PPIAP30 | 2.74E-10 | 1.93 |
| DTHD1 | 2.11E-06 | 1.93 |
| CCN4 | 4.22E-08 | 1.93 |
| TUNAR | 4.95E-03 | 1.93 |
| MSH5-SAPCD1 | 4.06E-19 | 1.93 |
| PFDN2 | 8.49E-21 | 1.93 |
| ZNF714 | 1.23E-12 | 1.93 |
| LINC01191 | 5.67E-06 | 1.93 |
| DUXAP8 | 1.10E-09 | 1.93 |
| LINC02210-CRHR1 | 4.00E-05 | 1.93 |
| SNORD52 | 5.55E-16 | 1.93 |
| H2AX | 3.33E-23 | 1.93 |
| CAPN14 | 3.27E-07 | 1.93 |
| TRAT1 | 5.38E-06 | 1.93 |
| RFC4 | 5.49E-20 | 1.93 |
| GPR132 | 3.95E-11 | 1.93 |
| SLC22A16 | 4.47E-04 | 1.93 |
| DERL3 | 1.97E-10 | 1.93 |
| SMG1-DT | 2.69E-15 | 1.92 |
| CPNE5 | 4.83E-09 | 1.92 |
| CCER2 | 1.19E-14 | 1.92 |
| BACH1-IT2 | 8.26E-09 | 1.92 |
| SMTNL1 | 1.87E-08 | 1.92 |
| AQP5-AS1 | 6.13E-04 | 1.92 |
| CTPS1 | 8.93E-16 | 1.92 |
| MIR6742 | 1.82E-14 | 1.92 |
| PFDN6 | 4.97E-31 | 1.92 |
| LINC01508 | 1.79E-05 | 1.92 |
| TESPA1 | 2.31E-07 | 1.92 |
| PCDH11X | 6.29E-04 | 1.92 |
| IGLV6-57 | 1.02E-03 | 1.92 |
| VEGFA | 3.02E-12 | 1.91 |
| MIR4517 | 9.62E-18 | 1.91 |
| SPDYE14 | 5.58E-17 | 1.91 |
| PRELID3A | 3.08E-17 | 1.91 |
| IGKV4-1 | 1.88E-03 | 1.91 |
| SH2D1A | 7.15E-06 | 1.91 |
| RBBP8NL | 1.57E-08 | 1.91 |
| PLS3-AS1 | 5.78E-07 | 1.91 |
| NPIPB3 | 2.31E-14 | 1.91 |
| FUT5 | 4.37E-06 | 1.91 |
| MIR6847 | 8.93E-10 | 1.91 |
| LINC02390 | 4.41E-06 | 1.91 |
| PCDH11Y | 3.07E-04 | 1.91 |
| IGLC6 | 6.35E-04 | 1.91 |
| NPIPB13 | 1.18E-13 | 1.91 |
| CAGE1 | 1.08E-10 | 1.91 |
| CKM | 3.07E-07 | 1.91 |
| SPDYE7P | 4.46E-14 | 1.91 |
| FABP7 | 1.37E-03 | 1.90 |
| CORO1A-AS1 | 8.81E-11 | 1.90 |
| LINC02809 | 6.44E-08 | 1.90 |
| VASH2 | 1.27E-08 | 1.90 |
| SEPTIN1 | 9.26E-09 | 1.90 |
| SUMO4 | 1.56E-13 | 1.90 |
| GZMK | 1.51E-05 | 1.90 |
| CHEK2 | 3.36E-26 | 1.90 |
| MIR6758 | 1.87E-15 | 1.90 |
| SCARNA12 | 1.47E-14 | 1.90 |
| IBA57-DT | 1.49E-10 | 1.90 |
| SATB1-AS1 | 1.29E-05 | 1.90 |
| LINC02515 | 1.23E-04 | 1.90 |
| RUNX3-AS1 | 8.19E-10 | 1.90 |
| SNORA80E | 4.78E-11 | 1.90 |
| TBC1D3K | 7.84E-11 | 1.89 |
| TMEM31 | 5.90E-06 | 1.89 |
| H3C13 | 8.10E-11 | 1.89 |
| LINC01754 | 1.48E-05 | 1.89 |
| SNORD33 | 9.69E-14 | 1.89 |
| SUSD4 | 1.30E-05 | 1.89 |
| LINC01449 | 4.07E-05 | 1.89 |
| P2RY10 | 1.44E-05 | 1.89 |
| TTC9-DT | 7.41E-06 | 1.89 |
| LINC01356 | 5.07E-07 | 1.89 |
| GPR55 | 6.18E-05 | 1.89 |
| CFAP418-AS1 | 1.79E-07 | 1.89 |
| LINC01993 | 1.24E-06 | 1.89 |
| ATP6V0D2 | 6.10E-05 | 1.89 |
| MIA | 3.47E-05 | 1.89 |
| LY6K | 2.62E-07 | 1.89 |
| CCDC92B | 8.22E-07 | 1.89 |
| ETV7 | 3.58E-12 | 1.89 |
| JSRP1 | 6.02E-06 | 1.89 |
| EGR4 | 5.24E-04 | 1.89 |
| MIR4690 | 2.33E-16 | 1.89 |
| GCNT2 | 1.38E-12 | 1.89 |
| NUP210L | 1.83E-10 | 1.89 |
| ASPHD1 | 1.03E-08 | 1.89 |
| NOP2 | 1.17E-17 | 1.89 |
| SIRPG | 7.19E-07 | 1.89 |
| ZNF724 | 2.20E-15 | 1.89 |
| TTBK1 | 1.64E-06 | 1.89 |
| EFCAB6-DT | 1.54E-06 | 1.89 |
| RFPL3 | 4.62E-14 | 1.89 |
| LINC00243 | 4.27E-09 | 1.89 |
| HOXC9 | 3.82E-09 | 1.88 |
| H2BC20P | 4.92E-13 | 1.88 |
| SNORD11 | 3.40E-10 | 1.88 |
| ABHD11-AS1 | 2.79E-10 | 1.88 |
| LINC02736 | 1.74E-06 | 1.88 |
| NKX6-1 | 2.07E-05 | 1.88 |
| WFIKKN1 | 1.84E-07 | 1.88 |
| H2AB2 | 2.25E-07 | 1.88 |
| OASL | 3.65E-07 | 1.88 |
| SDC1 | 9.12E-07 | 1.88 |
| TMSB10 | 9.80E-19 | 1.88 |
| OGFRP1 | 7.30E-19 | 1.88 |
| APOC1P1 | 2.82E-09 | 1.88 |
| E2F3 | 2.51E-20 | 1.88 |
| TNF | 2.74E-07 | 1.88 |
| CHAF1B | 4.44E-15 | 1.87 |
| SLC5A11 | 3.77E-06 | 1.87 |
| DCSTAMP | 2.66E-06 | 1.87 |
| NLRP2 | 2.91E-04 | 1.87 |
| PICSAR | 9.15E-05 | 1.87 |
| ASIC4 | 2.36E-08 | 1.87 |
| SNORD134 | 8.81E-05 | 1.87 |
| KCNK1 | 1.36E-09 | 1.87 |
| TMEM51-AS2 | 1.05E-10 | 1.87 |
| NEMP2-DT | 1.72E-11 | 1.87 |
| PAX6 | 1.71E-07 | 1.87 |
| LOC107987259 | 3.14E-07 | 1.87 |
| HMGB2 | 2.15E-17 | 1.87 |
| SCIRT | 9.63E-06 | 1.87 |
| NPIPB11 | 1.19E-13 | 1.87 |
| NEB | 1.90E-12 | 1.87 |
| TBX19 | 1.24E-15 | 1.87 |
| ITPKA | 4.73E-09 | 1.87 |
| ASAP1-IT2 | 3.19E-12 | 1.87 |
| H2BC15 | 1.92E-11 | 1.87 |
| RBIS | 1.85E-23 | 1.87 |
| LINC00937 | 3.27E-08 | 1.87 |
| POU2F2 | 1.71E-09 | 1.86 |
| NME8 | 6.06E-10 | 1.86 |
| TRGV1 | 4.00E-06 | 1.86 |
| SLC30A3 | 9.88E-07 | 1.86 |
| ZNF707 | 8.85E-14 | 1.86 |
| IGLV1-51 | 2.82E-04 | 1.86 |
| LINC01139 | 1.42E-03 | 1.86 |
| SNORD63 | 3.54E-10 | 1.86 |
| SWINGN | 5.36E-08 | 1.86 |
| ZNF663P | 1.74E-05 | 1.86 |
| DRD4 | 1.03E-09 | 1.86 |
| TLR9 | 2.13E-08 | 1.86 |
| UST-AS2 | 1.39E-10 | 1.86 |
| FANCI | 8.30E-26 | 1.86 |
| RFPL3S | 3.43E-15 | 1.86 |
| PVT1 | 5.61E-09 | 1.86 |
| TRGV2 | 1.41E-07 | 1.86 |
| PRR22 | 3.86E-10 | 1.86 |
| RAB26 | 1.16E-12 | 1.86 |
| DSCR9 | 2.59E-15 | 1.86 |
| SLC16A3 | 2.51E-10 | 1.86 |
| DNMT3B | 2.45E-15 | 1.85 |
| KRT6B | 1.10E-03 | 1.85 |
| TNNI2 | 1.89E-03 | 1.85 |
| TLR10 | 2.43E-04 | 1.85 |
| SNORD38A | 8.22E-12 | 1.85 |
| PMAIP1 | 1.98E-09 | 1.85 |
| GEN1 | 3.70E-23 | 1.85 |
| SNORD50B | 7.15E-15 | 1.85 |
| TRBJ2-7 | 2.42E-05 | 1.85 |
| SNORD87 | 1.64E-16 | 1.85 |
| IL4I1 | 4.42E-14 | 1.85 |
| TMEM262 | 7.58E-15 | 1.85 |
| LINC00299 | 4.30E-06 | 1.85 |
| QRICH2 | 2.39E-10 | 1.85 |
| RGS10 | 1.35E-13 | 1.85 |
| GSDMB | 2.39E-09 | 1.85 |
| PPFIA3 | 5.57E-09 | 1.85 |
| LCTL | 2.16E-14 | 1.85 |
| CEP250-AS1 | 5.18E-19 | 1.85 |
| BAAT | 6.35E-07 | 1.85 |
| SLC6A20 | 8.38E-05 | 1.85 |
| CLEC7A | 8.09E-12 | 1.84 |
| SNORD70 | 1.47E-11 | 1.84 |
| TRBC2 | 1.47E-06 | 1.84 |
| AOC1 | 2.52E-06 | 1.84 |
| ARHGEF2-AS2 | 3.49E-17 | 1.84 |
| ARFGEF1-DT | 1.20E-08 | 1.84 |
| KLHL38 | 7.55E-05 | 1.84 |
| BRPF3-AS1 | 1.46E-10 | 1.84 |
| DNAH11 | 4.63E-11 | 1.84 |
| GSTA2 | 2.09E-03 | 1.84 |
| COL27A1 | 4.82E-11 | 1.84 |
| HOXC13 | 1.25E-06 | 1.84 |
| LNCOC1 | 8.63E-08 | 1.84 |
| IGLJ1 | 2.47E-03 | 1.84 |
| DDX39A | 1.58E-44 | 1.84 |
| SNORA18 | 1.40E-15 | 1.84 |
| KNOP1 | 1.20E-20 | 1.84 |
| ARHGAP39 | 4.03E-10 | 1.83 |
| KRTDAP | 2.89E-04 | 1.83 |
| TUBA3D | 4.29E-12 | 1.83 |
| MIR6719 | 2.59E-08 | 1.83 |
| TBC1D32 | 3.26E-12 | 1.83 |
| MSH5 | 6.41E-19 | 1.83 |
| CDH2 | 5.89E-04 | 1.83 |
| FIBCD1 | 9.15E-05 | 1.83 |
| SPDYE15 | 8.10E-16 | 1.83 |
| ARHGAP11A-SCG5 | 5.25E-21 | 1.83 |
| CST6 | 5.66E-05 | 1.83 |
| WWOX-AS1 | 1.30E-07 | 1.83 |
| BMS1P21 | 1.06E-05 | 1.83 |
| KCNH8 | 2.39E-04 | 1.83 |
| UNC13D | 1.56E-12 | 1.83 |
| GPR21 | 2.60E-11 | 1.83 |
| SNORD35A | 1.03E-09 | 1.83 |
| LIPM | 4.96E-07 | 1.83 |
| TRS-AGA5-1 | 2.29E-09 | 1.83 |
| TRNR | 4.03E-06 | 1.83 |
| TNFSF11 | 1.91E-06 | 1.83 |
| MIR663A | 1.41E-08 | 1.83 |
| ZNF652-AS1 | 7.08E-06 | 1.82 |
| SNORD86 | 7.42E-17 | 1.82 |
| KDM2B-DT | 4.24E-09 | 1.82 |
| H2AC25 | 2.83E-10 | 1.82 |
| LOC112267973 | 6.50E-05 | 1.82 |
| PRSS3 | 4.89E-06 | 1.82 |
| SNORD54 | 2.51E-13 | 1.82 |
| SLFNL1 | 2.28E-19 | 1.82 |
| PACC1 | 7.07E-23 | 1.82 |
| IGHV1-69D | 1.71E-03 | 1.82 |
| HYDIN2 | 1.30E-05 | 1.82 |
| LINC02446 | 7.17E-08 | 1.82 |
| A1CF | 5.01E-05 | 1.82 |
| SPDL1 | 8.67E-21 | 1.82 |
| MST1R | 8.82E-12 | 1.82 |
| SLC35F2 | 4.99E-09 | 1.82 |
| LIME1 | 6.91E-17 | 1.82 |
| IGLV4-69 | 6.18E-03 | 1.82 |
| CASC15 | 4.59E-08 | 1.82 |
| MIR1245A | 1.96E-05 | 1.82 |
| MIR1245B | 1.96E-05 | 1.82 |
| PNMA3 | 2.49E-06 | 1.82 |
| TM4SF1-AS1 | 5.87E-06 | 1.82 |
| SNHG3 | 3.46E-17 | 1.82 |
| TYMS | 1.65E-23 | 1.82 |
| RNASEH2A | 3.08E-22 | 1.82 |
| KLC2-AS2 | 4.30E-12 | 1.81 |
| SOX4 | 6.71E-16 | 1.81 |
| ZNF692 | 3.42E-15 | 1.81 |
| SPDYE5 | 3.61E-17 | 1.81 |
| ANKRD18CP | 2.05E-08 | 1.81 |
| NRG4 | 2.47E-13 | 1.81 |
| DEPDC7 | 2.12E-14 | 1.81 |
| PRKCQ-AS1 | 2.90E-07 | 1.81 |
| SNORD58C | 1.01E-12 | 1.81 |
| JAKMIP2 | 5.35E-11 | 1.81 |
| NTNG1 | 2.69E-04 | 1.81 |
| DPH3P1 | 9.79E-12 | 1.81 |
| LOC642846 | 1.99E-14 | 1.81 |
| TYMP | 1.77E-12 | 1.81 |
| MLLT11 | 4.54E-10 | 1.81 |
| KHDC1 | 5.94E-13 | 1.81 |
| RNF222 | 5.67E-06 | 1.81 |
| TMSB15B-AS1 | 4.18E-05 | 1.81 |
| C1orf35 | 8.66E-27 | 1.81 |
| DRP2 | 3.37E-08 | 1.81 |
| RELT | 2.77E-16 | 1.81 |
| LIPK | 2.90E-03 | 1.80 |
| WDR35-DT | 2.25E-10 | 1.80 |
| FADS2 | 2.11E-06 | 1.80 |
| CALML6 | 3.51E-07 | 1.80 |
| TRGV4 | 3.83E-07 | 1.80 |
| SNORD81 | 1.58E-18 | 1.80 |
| C14orf39 | 8.09E-04 | 1.80 |
| RAC3 | 6.24E-13 | 1.80 |
| ZNF133-AS1 | 1.01E-09 | 1.80 |
| NPIPB4 | 8.42E-14 | 1.80 |
| SNORD17 | 4.04E-11 | 1.80 |
| LINC01600 | 1.66E-07 | 1.80 |
| UVRAG-DT | 1.94E-05 | 1.80 |
| LINC01535 | 2.10E-09 | 1.80 |
| LRRC69 | 1.28E-13 | 1.80 |
| CENPJ | 1.38E-17 | 1.80 |
| SLC6A17 | 1.28E-05 | 1.80 |
| GRASLND | 7.84E-09 | 1.80 |
| LINC01970 | 6.16E-08 | 1.80 |
| SMCR5 | 5.29E-09 | 1.80 |
| RNF182 | 1.64E-03 | 1.80 |
| SOCAR | 4.48E-18 | 1.80 |
| MIR4793 | 3.57E-06 | 1.80 |
| CCNF | 1.44E-17 | 1.80 |
| BMS1P22 | 4.24E-09 | 1.79 |
| DANT2 | 1.47E-06 | 1.79 |
| CCDC77 | 2.28E-14 | 1.79 |
| MIR7113 | 1.31E-14 | 1.79 |
| PRSS53 | 1.12E-22 | 1.79 |
| PART1 | 7.26E-06 | 1.79 |
| LLGL2 | 1.01E-12 | 1.79 |
| ZNF793 | 9.97E-11 | 1.79 |
| IGF2BP2-AS1 | 6.84E-05 | 1.79 |
| SMIM6 | 2.16E-13 | 1.79 |
| ELF3-AS1 | 7.74E-15 | 1.79 |
| LINC02908 | 4.61E-13 | 1.79 |
| MIR6849 | 1.83E-10 | 1.79 |
| CPA4 | 1.00E-05 | 1.79 |
| RRS1-DT | 6.21E-08 | 1.79 |
| ACTR3-AS1 | 1.65E-10 | 1.79 |
| CNFN | 9.63E-09 | 1.79 |
| KCNJ9 | 7.04E-09 | 1.79 |
| WASIR1 | 2.62E-05 | 1.79 |
| SLC17A9 | 2.27E-12 | 1.79 |
| RUNX2-AS1 | 1.68E-09 | 1.79 |
| SLC22A7 | 1.47E-05 | 1.79 |
| LOC107984397 | 2.39E-06 | 1.78 |
| FXYD7 | 9.14E-09 | 1.78 |
| AGK-DT | 4.11E-08 | 1.78 |
| KNSTRN | 2.96E-17 | 1.78 |
| LOC100505585 | 3.15E-07 | 1.78 |
| SRPK1 | 1.98E-25 | 1.78 |
| ZBTB32 | 7.50E-09 | 1.78 |
| LINC02970 | 1.15E-06 | 1.78 |
| MIR7111 | 6.04E-12 | 1.78 |
| HOXD13 | 2.90E-03 | 1.78 |
| ADAMTS6 | 3.56E-09 | 1.78 |
| MIR6820 | 5.19E-09 | 1.78 |
| SNORA58B | 6.81E-10 | 1.78 |
| MIR6878 | 2.10E-08 | 1.78 |
| FAM133A | 4.59E-04 | 1.78 |
| LINC01107 | 7.50E-06 | 1.78 |
| PDC-AS1 | 1.30E-10 | 1.78 |
| TRPV6 | 2.54E-05 | 1.78 |
| MIR4435-2 | 3.23E-07 | 1.78 |
| LINC00664 | 6.23E-06 | 1.78 |
| MIR4534 | 1.86E-04 | 1.78 |
| MROH9 | 1.28E-04 | 1.78 |
| ZNF726 | 3.87E-11 | 1.78 |
| PLAC4 | 9.23E-08 | 1.78 |
| CCDC57 | 2.74E-08 | 1.77 |
| PMCH | 1.07E-15 | 1.77 |
| C9orf50 | 1.09E-06 | 1.77 |
| DNAJC2 | 2.37E-19 | 1.77 |
| DPY19L3-DT | 2.20E-13 | 1.77 |
| RUNDC3A-AS1 | 1.71E-06 | 1.77 |
| PODXL2 | 5.04E-12 | 1.77 |
| IL2RG | 1.18E-07 | 1.77 |
| GORAB-AS1 | 3.30E-11 | 1.77 |
| IGHV3-65 | 6.02E-03 | 1.77 |
| C1QL1 | 4.85E-05 | 1.77 |
| PRR19 | 3.54E-14 | 1.77 |
| MAGEC3 | 1.00E-05 | 1.77 |
| RLBP1 | 2.85E-05 | 1.77 |
| CFAP276 | 6.35E-07 | 1.77 |
| CYP2D6 | 7.75E-13 | 1.77 |
| TEX50 | 1.17E-05 | 1.76 |
| ZNF252P-AS1 | 1.24E-14 | 1.76 |
| GBAP1 | 4.06E-16 | 1.76 |
| OBP2A | 3.20E-05 | 1.76 |
| NEFH | 1.06E-04 | 1.76 |
| KIF21A | 1.21E-14 | 1.76 |
| IL20RB | 9.73E-07 | 1.76 |
| TRBC1 | 1.52E-06 | 1.76 |
| TRY-ATA1-1 | 4.26E-11 | 1.76 |
| FZD9 | 2.57E-05 | 1.76 |
| H2AC15 | 7.84E-08 | 1.76 |
| LINC01410 | 2.64E-11 | 1.76 |
| ZNF556 | 1.09E-05 | 1.76 |
| INHBA-AS1 | 8.70E-07 | 1.76 |
| IGHV3-30 | 3.24E-03 | 1.76 |
| CHML | 5.20E-13 | 1.76 |
| LOC101929141 | 5.67E-08 | 1.76 |
| MEIG1 | 3.46E-06 | 1.76 |
| CUZD1 | 3.62E-10 | 1.76 |
| HIF1A-AS3 | 1.02E-09 | 1.76 |
| ADAMTS19 | 1.73E-03 | 1.76 |
| TFAP2A-AS2 | 5.47E-09 | 1.76 |
| FAR2P1 | 1.96E-03 | 1.76 |
| GPATCH4 | 1.34E-24 | 1.76 |
| CAPN12 | 4.01E-14 | 1.75 |
| LINC02788 | 4.36E-08 | 1.75 |
| KLHL17 | 1.49E-17 | 1.75 |
| LCT | 4.80E-04 | 1.75 |
| SYNDIG1 | 5.74E-06 | 1.75 |
| TMCC2 | 1.54E-06 | 1.75 |
| SLC24A3-AS1 | 4.52E-09 | 1.75 |
| TBX20 | 4.01E-03 | 1.75 |
| IGHV1-69 | 3.44E-03 | 1.75 |
| LINC00907 | 1.82E-05 | 1.75 |
| NPPA | 3.99E-07 | 1.75 |
| SNHG12 | 1.87E-17 | 1.75 |
| RPL19P12 | 1.87E-13 | 1.75 |
| PHOX2A | 1.61E-10 | 1.75 |
| TBC1D3L | 7.03E-11 | 1.75 |
| PAXX | 1.96E-23 | 1.75 |
| CORO1A | 4.64E-09 | 1.75 |
| ATP10B | 1.35E-06 | 1.75 |
| SPP1 | 1.11E-05 | 1.75 |
| GATA6-AS1 | 5.19E-06 | 1.75 |
| TNFSF4 | 2.24E-19 | 1.75 |
| LOC107986982 | 8.48E-08 | 1.75 |
| REC8 | 1.65E-14 | 1.75 |
| MIR4312 | 1.80E-08 | 1.74 |
| PRR36 | 8.77E-06 | 1.74 |
| S100A5 | 1.58E-05 | 1.74 |
| ATP4A | 1.12E-05 | 1.74 |
| LRRC45 | 2.78E-14 | 1.74 |
| IGLV1-41 | 1.21E-03 | 1.74 |
| CD2 | 8.17E-07 | 1.74 |
| COCH | 5.48E-05 | 1.74 |
| CSTB | 7.83E-13 | 1.74 |
| TMSB15B | 3.40E-08 | 1.74 |
| DPRXP4 | 4.83E-09 | 1.74 |
| C19orf33 | 4.10E-07 | 1.74 |
| ZNF239 | 1.45E-11 | 1.74 |
| IGLC3 | 1.11E-03 | 1.74 |
| HMGA1 | 2.39E-10 | 1.74 |
| TNKS2-DT | 9.52E-06 | 1.74 |
| IGKV3-15 | 2.78E-03 | 1.74 |
| E2F5 | 5.19E-13 | 1.74 |
| VIRMA-DT | 5.96E-09 | 1.74 |
| CBX8 | 1.06E-13 | 1.74 |
| FALEC | 4.33E-09 | 1.74 |
| SNORA7B | 3.68E-16 | 1.74 |
| STPG4 | 1.52E-07 | 1.74 |
| ACBD7 | 9.18E-06 | 1.74 |
| CFAP119 | 2.11E-16 | 1.74 |
| QPCT | 3.66E-06 | 1.73 |
| NRTN | 1.48E-06 | 1.73 |
| JARID2-AS1 | 8.62E-08 | 1.73 |
| NBPF6 | 1.77E-03 | 1.73 |
| PSCA | 5.06E-06 | 1.73 |
| RHCG | 3.56E-04 | 1.73 |
| DDC | 5.43E-04 | 1.73 |
| LINC02340 | 1.26E-07 | 1.73 |
| LINC02918 | 3.47E-12 | 1.73 |
| BRCA1 | 1.23E-11 | 1.73 |
| AP1S3 | 1.49E-11 | 1.73 |
| MIR4730 | 1.91E-06 | 1.73 |
| SNORD79 | 3.57E-14 | 1.73 |
| ING2-DT | 6.40E-14 | 1.73 |
| CHRM3 | 9.18E-05 | 1.73 |
| ZGRF1 | 1.58E-12 | 1.73 |
| MIR6729 | 4.82E-12 | 1.73 |
| PDE7A | 1.84E-16 | 1.73 |
| UBE2Q2P2 | 7.64E-16 | 1.73 |
| SOX2 | 1.33E-03 | 1.73 |
| TRPM5 | 5.54E-08 | 1.73 |
| PPP1R16A | 8.22E-19 | 1.73 |
| GP1BB | 6.98E-08 | 1.73 |
| TBC1D3E | 3.21E-10 | 1.73 |
| LINC00426 | 9.50E-06 | 1.72 |
| H4C15 | 9.73E-10 | 1.72 |
| OR1K1 | 2.90E-06 | 1.72 |
| DLEU7-AS1 | 4.01E-07 | 1.72 |
| SPDYE16 | 1.17E-13 | 1.72 |
| GPR35 | 2.02E-08 | 1.72 |
| LAD1 | 1.92E-06 | 1.72 |
| LOC105375513 | 1.65E-11 | 1.72 |
| GLYATL2 | 9.58E-04 | 1.72 |
| CHRNA5 | 1.06E-07 | 1.72 |
| TFAP2A-AS1 | 5.25E-11 | 1.72 |
| CLEC4F | 9.69E-04 | 1.72 |
| WASIR2 | 2.37E-05 | 1.72 |
| ODF3 | 1.13E-06 | 1.72 |
| FBXW10 | 1.23E-11 | 1.72 |
| LHB | 5.71E-08 | 1.72 |
| GOLGA6L9 | 3.41E-12 | 1.72 |
| MYADML2 | 3.49E-09 | 1.72 |
| CLDN16 | 8.02E-05 | 1.72 |
| FAM135B | 2.96E-03 | 1.72 |
| SLC35F4 | 1.10E-06 | 1.72 |
| MIR6859-4 | 1.48E-12 | 1.72 |
| FRMPD3 | 1.10E-04 | 1.72 |
| FOXD4L1 | 1.67E-08 | 1.72 |
| CACNA1I | 1.05E-05 | 1.72 |
| LRGUK | 2.58E-07 | 1.72 |
| SPDYE1 | 3.60E-13 | 1.72 |
| UPF3B | 7.74E-17 | 1.72 |
| NBAT1 | 1.04E-05 | 1.72 |
| H4C14 | 1.36E-09 | 1.71 |
| IGF2BP2 | 6.00E-05 | 1.71 |
| SNORD13D | 6.00E-06 | 1.71 |
| HOXC-AS2 | 2.80E-07 | 1.71 |
| KMO | 8.50E-19 | 1.71 |
| WDHD1 | 1.61E-19 | 1.71 |
| NPIPB15 | 4.50E-09 | 1.71 |
| MDFI | 1.41E-06 | 1.71 |
| LINC01943 | 3.38E-14 | 1.71 |
| IGLC2 | 1.11E-03 | 1.71 |
| CCDC144A | 1.44E-06 | 1.71 |
| EPHX3 | 2.90E-05 | 1.71 |
| MIR5695 | 3.57E-06 | 1.71 |
| CCZ1P-OR7E38P | 7.79E-20 | 1.71 |
| MIR4728 | 9.87E-08 | 1.71 |
| LRRC18 | 8.50E-06 | 1.71 |
| PSTPIP1 | 1.79E-11 | 1.71 |
| GOLGA6B | 7.03E-06 | 1.71 |
| RPL3L | 1.27E-07 | 1.71 |
| RDH10-AS1 | 6.59E-07 | 1.71 |
| HROB | 1.28E-19 | 1.71 |
| SLC23A3 | 6.67E-12 | 1.71 |
| PTMA | 6.17E-31 | 1.71 |
| ESRP1 | 1.23E-07 | 1.70 |
| KRTAP5-8 | 6.11E-09 | 1.70 |
| KRT74 | 2.06E-03 | 1.70 |
| LINC02604 | 6.78E-11 | 1.70 |
| USP12-AS1 | 1.40E-05 | 1.70 |
| BICDL2 | 1.65E-06 | 1.70 |
| WDR7-OT1 | 1.71E-08 | 1.70 |
| LOC107984284 | 2.49E-04 | 1.70 |
| SMG1P7 | 3.54E-11 | 1.70 |
| AFDN-DT | 5.93E-10 | 1.70 |
| H2BC4 | 5.72E-08 | 1.70 |
| SNX29P1 | 9.89E-09 | 1.70 |
| RNF139-DT | 3.67E-13 | 1.70 |
| MIR4489 | 7.35E-10 | 1.70 |
| LINC01725 | 2.74E-05 | 1.70 |
| SPDYE17 | 1.45E-15 | 1.70 |
| CAMK1G | 8.12E-07 | 1.70 |
| NGEF | 5.80E-06 | 1.70 |
| NME1 | 1.14E-15 | 1.70 |
| LOC105373826 | 9.39E-09 | 1.70 |
| LINC01544 | 1.04E-06 | 1.69 |
| LOC100419170 | 6.70E-07 | 1.69 |
| CD48 | 2.01E-05 | 1.69 |
| BARD1 | 9.08E-21 | 1.69 |
| SPDYE11 | 2.09E-15 | 1.69 |
| DDX11L16 | 2.65E-06 | 1.69 |
| ALKAL2 | 3.97E-03 | 1.69 |
| PRR7 | 1.05E-09 | 1.69 |
| CPT1B | 3.11E-12 | 1.69 |
| PDIA2 | 3.31E-04 | 1.69 |
| FOXD4L3 | 7.76E-10 | 1.69 |
| SNORD76 | 2.14E-08 | 1.69 |
| PRKCG | 1.70E-07 | 1.69 |
| ZNF469 | 9.74E-09 | 1.69 |
| ZNF682 | 7.59E-12 | 1.69 |
| PTPRCAP | 1.54E-07 | 1.69 |
| TRAIP | 4.83E-20 | 1.69 |
| ZNF492 | 3.44E-07 | 1.69 |
| MIR5587 | 6.46E-10 | 1.69 |
| HOMER3 | 3.62E-19 | 1.69 |
| CKAP2 | 2.22E-15 | 1.69 |
| HLA-DOB | 2.95E-06 | 1.69 |
| ZFP69B | 5.46E-13 | 1.69 |
| CREG2 | 2.91E-07 | 1.69 |
| APOBEC3A | 1.32E-07 | 1.69 |
| RTP5 | 6.06E-05 | 1.69 |
| MAP3K9-DT | 1.12E-09 | 1.69 |
| RNF224 | 4.34E-05 | 1.69 |
| LINC00528 | 2.91E-06 | 1.69 |
| MMS22L | 2.22E-16 | 1.69 |
| FCMR | 2.11E-04 | 1.69 |
| LINC00115 | 4.59E-14 | 1.68 |
| LOC100505909 | 1.81E-06 | 1.68 |
| GOLGA6L5P | 1.45E-06 | 1.68 |
| CCDC65 | 3.22E-10 | 1.68 |
| ZNF93 | 1.13E-13 | 1.68 |
| S100A3 | 2.48E-05 | 1.68 |
| LINC00839 | 1.62E-06 | 1.68 |
| PHLDA2 | 4.20E-06 | 1.68 |
| STAG3 | 3.35E-07 | 1.68 |
| KIAA1755 | 1.93E-07 | 1.68 |
| AXDND1 | 5.13E-07 | 1.68 |
| INTS13 | 1.59E-13 | 1.68 |
| ANP32E | 1.41E-14 | 1.68 |
| TMC8 | 2.72E-07 | 1.68 |
| FAM220CP | 2.30E-07 | 1.68 |
| CARMIL3 | 7.39E-09 | 1.68 |
| LOC84214 | 4.89E-09 | 1.68 |
| EXTL3-AS1 | 1.21E-07 | 1.68 |
| ST3GAL3-AS1 | 1.15E-08 | 1.68 |
| MAGED4 | 2.20E-06 | 1.68 |
| COA6 | 3.42E-18 | 1.68 |
| ECT2 | 3.00E-10 | 1.68 |
| MIR6753 | 2.85E-10 | 1.68 |
| DDX11L5 | 2.38E-05 | 1.67 |
| LOC107985786 | 8.90E-10 | 1.67 |
| MIR3150BHG | 9.28E-08 | 1.67 |
| LDHC | 3.02E-05 | 1.67 |
| MELTF-AS1 | 1.63E-11 | 1.67 |
| C11orf80 | 7.13E-23 | 1.67 |
| MAGED4B | 2.27E-06 | 1.67 |
| FOXI3 | 3.37E-03 | 1.67 |
| MOXD1 | 5.58E-05 | 1.67 |
| LRFN2 | 4.16E-04 | 1.67 |
| P2RX2 | 4.02E-05 | 1.67 |
| TMEFF1 | 1.93E-09 | 1.67 |
| PLCH1 | 3.48E-06 | 1.67 |
| PYY2 | 1.07E-09 | 1.67 |
| MMP20-AS1 | 1.57E-03 | 1.67 |
| CTLA4 | 1.22E-06 | 1.67 |
| LINC01424 | 4.64E-12 | 1.67 |
| MIR503HG | 4.10E-06 | 1.67 |
| MIR6845 | 5.51E-10 | 1.67 |
| MIR6859-1 | 1.27E-11 | 1.67 |
| CRABP2 | 5.40E-05 | 1.67 |
| MED15P9 | 2.55E-04 | 1.67 |
| MAT1A | 1.57E-03 | 1.67 |
| FTCD-AS1 | 6.11E-06 | 1.67 |
| TBX1 | 3.25E-06 | 1.67 |
| IL24 | 9.61E-05 | 1.66 |
| TRBV20-1 | 1.75E-04 | 1.66 |
| SNHG1 | 3.54E-15 | 1.66 |
| DNAI1 | 2.66E-04 | 1.66 |
| SLC4A11 | 1.07E-04 | 1.66 |
| LINC02048 | 7.44E-06 | 1.66 |
| ZNF300 | 1.10E-06 | 1.66 |
| SFTPB | 6.50E-10 | 1.66 |
| CYP2D7 | 2.61E-14 | 1.66 |
| CMTM2 | 3.47E-08 | 1.66 |
| PHACTR2-AS1 | 1.65E-09 | 1.66 |
| FLAD1 | 3.81E-22 | 1.66 |
| HTR6 | 1.90E-05 | 1.66 |
| ARSL | 5.25E-07 | 1.66 |
| LINC00885 | 4.62E-06 | 1.66 |
| MALAT1 | 9.36E-09 | 1.66 |
| PUM3 | 5.97E-16 | 1.66 |
| TALAM1 | 9.31E-09 | 1.66 |
| MIR6859-2 | 4.42E-12 | 1.66 |
| TUBA4B | 2.48E-08 | 1.66 |
| SLC34A1 | 7.24E-07 | 1.66 |
| B4GAT1-DT | 4.68E-12 | 1.66 |
| APBA2 | 3.60E-08 | 1.66 |
| CYP27B1 | 1.34E-07 | 1.66 |
| CEP295 | 9.00E-14 | 1.66 |
| LOC105371036 | 4.06E-10 | 1.66 |
| CTRC | 1.06E-07 | 1.66 |
| MRPL14 | 8.44E-14 | 1.66 |
| BIRC6-AS2 | 3.90E-12 | 1.65 |
| GTF2IP20 | 7.40E-13 | 1.65 |
| POLE | 2.29E-12 | 1.65 |
| TRBV5-1 | 8.78E-04 | 1.65 |
| ZNF876P | 6.84E-07 | 1.65 |
| LRRC63 | 2.23E-08 | 1.65 |
| PBX1-AS1 | 9.76E-08 | 1.65 |
| SNORA40B | 9.83E-10 | 1.65 |
| DONSON | 2.00E-17 | 1.65 |
| CATSPERG | 2.19E-06 | 1.65 |
| TWSG1-DT | 4.31E-07 | 1.65 |
| SPAG4 | 2.83E-14 | 1.65 |
| GOLT1A | 1.66E-06 | 1.65 |
| C1orf74 | 4.01E-08 | 1.65 |
| IGLL5 | 3.07E-03 | 1.65 |
| MCMDC2 | 8.96E-16 | 1.65 |
| C9orf24 | 7.68E-09 | 1.65 |
| MIR5194 | 1.73E-09 | 1.65 |
| ADAM19 | 1.91E-10 | 1.65 |
| PLAG1 | 1.80E-05 | 1.65 |
| P4HA3 | 1.13E-05 | 1.65 |
| PCDHA4 | 1.01E-04 | 1.65 |
| PRR7-AS1 | 4.09E-14 | 1.65 |
| ATAD3B | 5.08E-16 | 1.65 |
| IER5L | 3.81E-14 | 1.64 |
| SIT1 | 1.05E-04 | 1.64 |
| NKTR | 2.78E-09 | 1.64 |
| TEX29 | 5.18E-07 | 1.64 |
| MIR3176 | 7.30E-07 | 1.64 |
| IRF7 | 3.37E-11 | 1.64 |
| MX1 | 3.26E-06 | 1.64 |
| ITGAD | 5.02E-08 | 1.64 |
| MIR5690 | 9.58E-04 | 1.64 |
| RAI1-AS1 | 1.98E-10 | 1.64 |
| TIGD1 | 4.58E-13 | 1.64 |
| AGER | 7.91E-12 | 1.64 |
| COMTD1 | 1.30E-12 | 1.64 |
| NLRC3 | 1.71E-07 | 1.64 |
| KCTD13-DT | 1.17E-15 | 1.64 |
| HAUS8 | 1.90E-21 | 1.64 |
| S100A11 | 1.24E-13 | 1.64 |
| CCDC144NL-AS1 | 3.13E-04 | 1.64 |
| SNORD48 | 2.80E-11 | 1.64 |
| LOC107987118 | 1.48E-07 | 1.64 |
| SEM1 | 1.14E-18 | 1.64 |
| SNORD12C | 1.02E-09 | 1.64 |
| MIR6859-3 | 2.30E-11 | 1.64 |
| SRD5A1 | 9.88E-11 | 1.64 |
| MIR3658 | 1.05E-16 | 1.64 |
| CCDC181 | 1.36E-06 | 1.64 |
| HR | 3.90E-05 | 1.64 |
| LIPG | 5.93E-05 | 1.64 |
| DPF1 | 4.17E-08 | 1.64 |
| RGS13 | 1.36E-04 | 1.64 |
| LRRC15 | 3.45E-04 | 1.64 |
| PEG3-AS1 | 1.90E-03 | 1.64 |
| SGSM3-AS1 | 3.71E-10 | 1.64 |
| MYCN | 1.27E-04 | 1.64 |
| SNHG19 | 1.44E-10 | 1.64 |
| CRYBA1 | 7.11E-05 | 1.64 |
| ANK2-AS1 | 1.49E-04 | 1.63 |
| FOXD4L6 | 2.97E-09 | 1.63 |
| IFI44 | 2.53E-07 | 1.63 |
| SEPTIN3 | 1.03E-05 | 1.63 |
| UCHL1 | 9.23E-04 | 1.63 |
| PCDH8 | 1.91E-03 | 1.63 |
| NCF1B | 1.39E-05 | 1.63 |
| MUC12 | 2.49E-06 | 1.63 |
| LOC107986800 | 1.60E-06 | 1.63 |
| ITPK1-AS1 | 5.78E-07 | 1.63 |
| ERVMER34-1 | 2.80E-04 | 1.63 |
| SMIM18 | 5.58E-08 | 1.63 |
| GPR173 | 7.11E-06 | 1.63 |
| LOC107986755 | 1.03E-08 | 1.63 |
| IGHJ3P | 4.28E-03 | 1.63 |
| LENG8-AS1 | 3.59E-11 | 1.63 |
| LOC112268171 | 9.16E-07 | 1.63 |
| SLC6A16 | 6.29E-09 | 1.63 |
| DDX11L10 | 4.36E-07 | 1.63 |
| FLVCR1 | 3.07E-19 | 1.63 |
| THBS3-AS1 | 1.07E-09 | 1.63 |
| IGLC1 | 3.52E-03 | 1.63 |
| LINC02244 | 1.27E-07 | 1.63 |
| CALR4P | 1.11E-05 | 1.63 |
| ZGLP1 | 3.08E-09 | 1.63 |
| BACH1-IT3 | 1.99E-08 | 1.63 |
| DNER | 2.66E-03 | 1.63 |
| RACGAP1 | 4.05E-11 | 1.63 |
| LINC01002 | 5.49E-12 | 1.63 |
| ZNF233 | 4.97E-12 | 1.63 |
| SNORD168 | 9.60E-09 | 1.63 |
| CLEC2D | 1.55E-11 | 1.63 |
| TTLL2 | 2.31E-04 | 1.63 |
| BRICD5 | 1.39E-16 | 1.62 |
| FAM161A | 3.61E-15 | 1.62 |
| PDE6G | 5.86E-07 | 1.62 |
| SH2D6 | 1.27E-05 | 1.62 |
| NMRAL2P | 4.68E-04 | 1.62 |
| SLAMF9 | 5.74E-05 | 1.62 |
| LIN28A | 1.52E-07 | 1.62 |
| MIA-RAB4B | 2.00E-09 | 1.62 |
| PAM16 | 1.37E-24 | 1.62 |
| ATP6V0D1-DT | 1.10E-09 | 1.62 |
| ENHO | 2.60E-05 | 1.62 |
| SLC7A9 | 2.28E-07 | 1.62 |
| MIR6840 | 2.60E-13 | 1.62 |
| IL18 | 5.96E-15 | 1.62 |
| C1orf220 | 1.09E-09 | 1.62 |
| FRG1DP | 8.61E-06 | 1.62 |
| RAB33B-AS1 | 1.16E-07 | 1.62 |
| CD44-DT | 7.40E-06 | 1.62 |
| ANXA8 | 4.20E-03 | 1.62 |
| DYNLT2B | 6.83E-20 | 1.62 |
| LZTS1-AS1 | 3.08E-06 | 1.61 |
| NCF1 | 1.02E-05 | 1.61 |
| MYBPC3 | 9.64E-08 | 1.61 |
| SMC6 | 1.03E-22 | 1.61 |
| OCA2 | 1.04E-03 | 1.61 |
| ZNF528-AS1 | 1.78E-06 | 1.61 |
| LOC105371427 | 2.94E-09 | 1.61 |
| OLR1 | 2.92E-06 | 1.61 |
| ZFAND3-DT | 1.58E-06 | 1.61 |
| DISC2 | 4.04E-05 | 1.61 |
| LYPD5 | 5.80E-06 | 1.61 |
| SLC16A8 | 1.68E-09 | 1.61 |
| ND4L | 4.45E-07 | 1.61 |
| ACOT12 | 3.03E-11 | 1.61 |
| MSH4 | 2.59E-07 | 1.61 |
| FHAD1 | 5.73E-08 | 1.61 |
| MYCNOS | 1.62E-04 | 1.61 |
| MIR6750 | 1.04E-08 | 1.61 |
| SNHG9 | 5.80E-09 | 1.61 |
| CCDC14 | 3.88E-13 | 1.61 |
| CHD7 | 1.14E-09 | 1.61 |
| LTK | 7.43E-05 | 1.61 |
| MIR4326 | 2.41E-07 | 1.61 |
| LINC02680 | 1.96E-07 | 1.61 |
| CYCSP52 | 1.78E-07 | 1.61 |
| PFKFB4 | 7.27E-09 | 1.60 |
| NLRP7 | 1.83E-05 | 1.60 |
| VWA5B2 | 4.26E-06 | 1.60 |
| CHRNA3 | 9.20E-10 | 1.60 |
| SEPTIN12 | 1.87E-04 | 1.60 |
| DUOXA1 | 8.72E-05 | 1.60 |
| KRT23 | 9.52E-04 | 1.60 |
| KLK14 | 6.04E-05 | 1.60 |
| TRBV7-9 | 9.10E-04 | 1.60 |
| ASIP | 5.80E-07 | 1.60 |
| ALMS1-IT1 | 1.49E-16 | 1.60 |
| IGKV3-20 | 4.82E-03 | 1.60 |
| SLAMF8 | 8.21E-12 | 1.60 |
| STAU2-AS1 | 9.20E-10 | 1.60 |
| CXCR3 | 1.28E-05 | 1.60 |
| BTG2-DT | 1.45E-08 | 1.60 |
| CAPN10-DT | 1.10E-21 | 1.60 |
| LOC105371461 | 6.36E-04 | 1.60 |
| PLXNA3 | 2.05E-13 | 1.60 |
| C20orf204 | 3.98E-09 | 1.60 |
| DCAF4L1 | 2.22E-09 | 1.60 |
| MICAL1 | 1.36E-11 | 1.60 |
| MIR6772 | 4.80E-06 | 1.60 |
| H2AZ1 | 8.57E-12 | 1.60 |
| REREP3 | 3.17E-07 | 1.60 |
| TIGD3 | 6.84E-09 | 1.60 |
| PILRB | 2.70E-10 | 1.60 |
| LINC01001 | 2.54E-12 | 1.59 |
| LINC02595 | 2.55E-05 | 1.59 |
| GOLGA2P6 | 3.85E-05 | 1.59 |
| ANKRD26 | 4.93E-11 | 1.59 |
| NPPC | 9.49E-04 | 1.59 |
| CACNB1 | 2.31E-07 | 1.59 |
| MINAR1 | 2.17E-12 | 1.59 |
| EFNA4 | 1.27E-12 | 1.59 |
| CENPX | 1.01E-12 | 1.59 |
| TMEM86B | 6.75E-14 | 1.59 |
| TUBA3E | 5.35E-09 | 1.59 |
| NXPE2 | 3.53E-07 | 1.59 |
| KHDRBS2 | 1.36E-03 | 1.59 |
| GOLGA6A | 7.34E-06 | 1.59 |
| MIR3142HG | 2.68E-05 | 1.59 |
| CSPP1 | 1.24E-11 | 1.59 |
| ARHGAP22 | 9.79E-15 | 1.59 |
| C2CD4D | 3.11E-07 | 1.59 |
| STRIP2 | 5.72E-12 | 1.59 |
| FOXC1 | 4.21E-09 | 1.59 |
| IL9R | 3.04E-06 | 1.59 |
| CFAP53 | 2.39E-06 | 1.59 |
| FAM106B | 1.36E-09 | 1.59 |
| ARID3C | 2.08E-09 | 1.59 |
| CRYGS | 2.58E-07 | 1.59 |
| VPS9D1-AS1 | 1.68E-13 | 1.59 |
| CHGB | 8.10E-04 | 1.59 |
| FUT3 | 2.15E-03 | 1.59 |
| GHET1 | 4.58E-11 | 1.59 |
| ZNF367 | 8.19E-10 | 1.59 |
| CEP131 | 4.47E-13 | 1.59 |
| MAP1A | 1.20E-08 | -1.59 |
| GABARAPL3 | 2.23E-12 | -1.59 |
| NCOA4 | 8.64E-17 | -1.59 |
| NRG1 | 5.47E-03 | -1.59 |
| MAGEB17 | 1.33E-03 | -1.59 |
| APCDD1 | 1.26E-05 | -1.59 |
| ODF3L1 | 1.64E-08 | -1.59 |
| DAAM2-AS1 | 1.34E-08 | -1.59 |
| JUNB | 3.54E-09 | -1.59 |
| CPZ | 1.50E-06 | -1.59 |
| DACH1 | 1.12E-06 | -1.59 |
| HPGDS | 1.20E-07 | -1.59 |
| GGTA1 | 1.06E-09 | -1.59 |
| LURAP1 | 1.75E-11 | -1.59 |
| MYADM | 2.90E-09 | -1.59 |
| CTNNA3 | 1.77E-06 | -1.59 |
| PRKAR1A | 1.94E-19 | -1.59 |
| CARMN | 1.86E-06 | -1.59 |
| ETS2 | 2.14E-10 | -1.59 |
| MYOM3 | 1.80E-06 | -1.59 |
| LONRF2 | 4.69E-05 | -1.59 |
| CBLN4 | 1.36E-03 | -1.60 |
| YPEL2 | 5.58E-17 | -1.60 |
| RNF157-AS1 | 1.06E-06 | -1.60 |
| PDE1B | 4.72E-09 | -1.60 |
| EFCAB14 | 3.11E-20 | -1.60 |
| KIAA0040 | 2.89E-10 | -1.60 |
| TMEM144 | 2.61E-14 | -1.60 |
| STARD13 | 1.09E-10 | -1.60 |
| FLRT3 | 3.86E-04 | -1.60 |
| MEGF9 | 3.24E-10 | -1.60 |
| FFAR2 | 1.31E-05 | -1.60 |
| ACADS | 5.35E-17 | -1.61 |
| SUCLA2 | 4.96E-16 | -1.61 |
| ZFHX4 | 1.77E-06 | -1.61 |
| CLDN8 | 2.16E-03 | -1.61 |
| PCCA | 1.72E-26 | -1.61 |
| SLC25A48 | 3.16E-06 | -1.61 |
| FUT1 | 9.78E-09 | -1.61 |
| SNRK-AS1 | 9.18E-16 | -1.61 |
| SYNE1 | 2.82E-13 | -1.61 |
| AQP7P3 | 3.10E-03 | -1.61 |
| RGL1 | 3.01E-17 | -1.61 |
| PNPLA4 | 1.31E-10 | -1.61 |
| PPP2R1B | 1.07E-11 | -1.61 |
| TPRG1 | 3.75E-10 | -1.61 |
| EGFR-AS1 | 5.64E-08 | -1.61 |
| RTN4RL1 | 6.28E-06 | -1.61 |
| GUCY1A2 | 6.45E-05 | -1.61 |
| GYPC | 2.94E-08 | -1.62 |
| DIPK1A | 1.27E-17 | -1.62 |
| PDK2 | 1.16E-11 | -1.62 |
| LAMC3 | 8.21E-06 | -1.62 |
| PNMA1 | 1.03E-15 | -1.62 |
| SCGB2A1 | 4.04E-03 | -1.62 |
| TSHZ1 | 1.05E-24 | -1.62 |
| VKORC1L1 | 1.04E-08 | -1.62 |
| ENG | 1.61E-12 | -1.62 |
| TRIM8 | 5.12E-16 | -1.62 |
| ADAM22 | 9.81E-12 | -1.62 |
| GULP1 | 2.49E-11 | -1.62 |
| SLC66A1L | 1.09E-04 | -1.62 |
| CAV3 | 2.10E-04 | -1.62 |
| ART4 | 7.19E-06 | -1.62 |
| ADAMTS9-AS1 | 9.98E-07 | -1.62 |
| BHMT | 1.01E-06 | -1.62 |
| COL4A2-AS2 | 6.05E-08 | -1.62 |
| TBX5 | 5.25E-06 | -1.62 |
| CADPS2 | 1.15E-09 | -1.62 |
| PHYHD1 | 4.69E-06 | -1.63 |
| BST1 | 4.45E-08 | -1.63 |
| EPB41L4A-DT | 7.02E-09 | -1.63 |
| APBB2 | 6.78E-17 | -1.63 |
| FAM228A | 2.10E-06 | -1.63 |
| EBF3-AS1 | 5.24E-06 | -1.63 |
| PEX19 | 1.38E-11 | -1.63 |
| MAMDC2 | 7.91E-26 | -1.63 |
| RNF180 | 4.19E-14 | -1.63 |
| TSC22D3 | 4.85E-09 | -1.63 |
| SLC25A20 | 1.68E-17 | -1.63 |
| HOXA5 | 4.99E-06 | -1.64 |
| CASKIN2 | 1.14E-14 | -1.64 |
| PCNX1 | 5.52E-37 | -1.64 |
| EPB41L3 | 3.43E-08 | -1.64 |
| THBS1 | 4.45E-07 | -1.64 |
| FOXO4 | 1.44E-14 | -1.64 |
| TEAD1 | 2.90E-14 | -1.64 |
| IRX6 | 3.08E-04 | -1.64 |
| RAG1 | 4.01E-13 | -1.64 |
| IQSEC3 | 9.44E-06 | -1.64 |
| ICAM2 | 6.35E-09 | -1.64 |
| CBFA2T3 | 2.13E-06 | -1.64 |
| MRO | 2.22E-06 | -1.64 |
| RBM24 | 4.03E-05 | -1.64 |
| CDS2 | 3.21E-21 | -1.64 |
| GAS1 | 8.52E-09 | -1.64 |
| NACC2 | 7.17E-11 | -1.64 |
| EHHADH-AS1 | 8.48E-08 | -1.64 |
| ASIC2 | 2.26E-05 | -1.65 |
| MRTFB | 6.50E-33 | -1.65 |
| OPN4 | 7.22E-06 | -1.65 |
| MKX | 1.24E-06 | -1.65 |
| PRND | 1.00E-03 | -1.65 |
| CEBPA-DT | 7.74E-11 | -1.65 |
| LINC01963 | 2.19E-11 | -1.65 |
| LTBP4 | 1.91E-08 | -1.65 |
| ENTPD3 | 1.94E-07 | -1.65 |
| NAV2-AS6 | 1.35E-07 | -1.65 |
| PHLDB1 | 4.36E-13 | -1.65 |
| FBLN5 | 1.69E-06 | -1.66 |
| RALGAPA2 | 1.98E-15 | -1.66 |
| FMO1 | 4.10E-07 | -1.66 |
| SLIT2 | 1.82E-05 | -1.66 |
| LOC100286986 | 7.52E-05 | -1.66 |
| EHHADH | 4.00E-11 | -1.66 |
| MCC | 2.17E-13 | -1.66 |
| FZD5 | 1.75E-10 | -1.66 |
| BABAM2-AS1 | 5.09E-16 | -1.66 |
| MAGEL2 | 1.30E-05 | -1.66 |
| MCTP1 | 4.94E-13 | -1.66 |
| ADCY6 | 2.76E-16 | -1.66 |
| CYP4A11 | 8.56E-04 | -1.66 |
| FGFBP2 | 7.44E-04 | -1.66 |
| PCDH18 | 2.36E-08 | -1.66 |
| LINC02798 | 2.01E-06 | -1.66 |
| RTL9 | 2.21E-06 | -1.66 |
| EIF4EBP2 | 8.29E-15 | -1.66 |
| RNASE1 | 1.77E-07 | -1.66 |
| RNF125 | 2.50E-12 | -1.66 |
| SLC25A25 | 2.14E-16 | -1.67 |
| CLCA4-AS1 | 1.01E-12 | -1.67 |
| ZNF853 | 5.12E-09 | -1.67 |
| KCNK15-AS1 | 8.84E-07 | -1.67 |
| TMEM35A | 4.83E-04 | -1.67 |
| ELK3 | 4.91E-15 | -1.67 |
| KIRREL1 | 1.55E-09 | -1.67 |
| NAALAD2 | 2.80E-14 | -1.67 |
| MIR6506 | 2.68E-21 | -1.67 |
| C10orf82 | 2.93E-03 | -1.67 |
| ALDH3A2 | 5.99E-11 | -1.68 |
| ERICH3 | 2.10E-04 | -1.68 |
| PPP1R12B | 4.37E-14 | -1.68 |
| PRIMA1 | 2.48E-05 | -1.68 |
| HSDL2 | 2.04E-16 | -1.68 |
| LRCH2 | 1.39E-09 | -1.68 |
| SPARC | 2.99E-06 | -1.68 |
| MYL3 | 4.79E-05 | -1.68 |
| ACSS2 | 1.19E-15 | -1.68 |
| RAB27B | 1.39E-05 | -1.68 |
| SNRK | 1.23E-25 | -1.68 |
| FAT3 | 2.12E-05 | -1.68 |
| STON1-GTF2A1L | 3.94E-11 | -1.68 |
| RPS6KA2 | 8.67E-15 | -1.68 |
| MFAP5 | 2.23E-04 | -1.68 |
| ALB | 2.04E-03 | -1.68 |
| ZBTB7C | 1.17E-07 | -1.69 |
| PDLIM1 | 3.06E-10 | -1.69 |
| FOXN1 | 4.13E-04 | -1.69 |
| ACOT1 | 2.30E-13 | -1.69 |
| CLCA2 | 5.61E-03 | -1.69 |
| CNTNAP3B | 4.55E-07 | -1.69 |
| EGFR | 9.99E-09 | -1.69 |
| HSPB2 | 1.80E-06 | -1.69 |
| SH3RF3-AS1 | 1.23E-09 | -1.69 |
| PTN | 1.39E-03 | -1.69 |
| DOCK9 | 8.88E-22 | -1.69 |
| PRKD1 | 1.45E-17 | -1.69 |
| PIP5K1B | 2.51E-06 | -1.69 |
| LINC01352 | 7.22E-08 | -1.69 |
| FOXI2 | 1.19E-04 | -1.69 |
| CCDC184 | 7.73E-08 | -1.70 |
| KRT32 | 4.16E-04 | -1.70 |
| RERG | 1.43E-06 | -1.70 |
| SLC7A8 | 1.97E-08 | -1.70 |
| DDO | 2.55E-08 | -1.70 |
| PTPN21 | 1.66E-17 | -1.70 |
| MITF | 1.36E-11 | -1.70 |
| LIMA1 | 8.18E-19 | -1.70 |
| GBE1 | 1.17E-10 | -1.70 |
| SHANK3 | 5.40E-10 | -1.70 |
| MIR6717 | 5.70E-09 | -1.70 |
| CLIC2 | 1.30E-12 | -1.70 |
| LOC103611081 | 4.50E-21 | -1.70 |
| PCYOX1 | 8.02E-14 | -1.70 |
| CEP68 | 1.63E-33 | -1.70 |
| RELL1 | 1.10E-18 | -1.70 |
| TMEM220-AS1 | 2.09E-15 | -1.70 |
| GSTM3 | 2.65E-08 | -1.71 |
| CYP26A1 | 5.09E-04 | -1.71 |
| CCN1 | 1.56E-11 | -1.71 |
| DSC1 | 4.62E-10 | -1.71 |
| IGIP | 8.63E-16 | -1.71 |
| PSG8-AS1 | 1.79E-04 | -1.71 |
| CARD10 | 3.24E-09 | -1.71 |
| NR3C1 | 5.23E-20 | -1.71 |
| C8orf34 | 8.47E-06 | -1.71 |
| KLF10 | 1.02E-10 | -1.71 |
| TAMALIN-AS1 | 1.08E-09 | -1.71 |
| CFL2 | 7.52E-20 | -1.72 |
| ESRRB | 2.39E-07 | -1.72 |
| TLCD2 | 1.11E-09 | -1.72 |
| AMPD1 | 6.69E-04 | -1.72 |
| HIPK3 | 7.07E-19 | -1.72 |
| PEX11A | 2.85E-18 | -1.72 |
| MIR6513 | 2.35E-12 | -1.72 |
| CCNDBP1 | 1.86E-23 | -1.72 |
| PDLIM3 | 2.62E-06 | -1.72 |
| AGPAT2 | 7.36E-08 | -1.72 |
| FBXL5 | 1.19E-28 | -1.72 |
| IL17B | 3.94E-05 | -1.72 |
| TNFRSF1B | 2.67E-12 | -1.72 |
| PABPC5 | 4.43E-10 | -1.72 |
| SGCB | 4.30E-13 | -1.72 |
| MYOM2 | 2.03E-08 | -1.73 |
| CYS1 | 1.53E-08 | -1.73 |
| MFNG | 9.65E-09 | -1.73 |
| FYCO1 | 5.07E-20 | -1.73 |
| SMIM3 | 6.14E-10 | -1.73 |
| MXRA7 | 1.01E-10 | -1.73 |
| OVCH1-AS1 | 3.88E-05 | -1.73 |
| PJA2 | 1.02E-30 | -1.73 |
| SERPINA5 | 3.30E-04 | -1.73 |
| PINK1 | 4.75E-17 | -1.73 |
| CREBL2 | 1.62E-13 | -1.73 |
| SLC26A5-AS1 | 8.90E-04 | -1.73 |
| SNED1 | 4.14E-14 | -1.73 |
| SNN | 1.21E-12 | -1.73 |
| LOC107984638 | 5.65E-12 | -1.73 |
| RNF150 | 6.86E-06 | -1.74 |
| NIBAN1 | 6.68E-13 | -1.74 |
| IHO1 | 9.11E-11 | -1.74 |
| IRS2 | 1.64E-06 | -1.74 |
| GIMAP5 | 6.92E-08 | -1.74 |
| NHSL2 | 7.25E-13 | -1.74 |
| SEMA3A | 5.24E-06 | -1.74 |
| ABHD15 | 5.12E-14 | -1.74 |
| NUDT6 | 1.96E-21 | -1.74 |
| NFIA | 1.89E-11 | -1.74 |
| PTPRN2 | 9.04E-09 | -1.74 |
| EGFL7 | 2.59E-09 | -1.74 |
| TCF23 | 1.17E-05 | -1.74 |
| DSEL | 1.19E-08 | -1.74 |
| FOXN3 | 1.61E-19 | -1.74 |
| FLRT2 | 7.78E-07 | -1.74 |
| CFI | 6.08E-10 | -1.74 |
| MS4A2 | 5.05E-06 | -1.74 |
| FAM187A | 1.68E-09 | -1.74 |
| HTR7 | 2.59E-10 | -1.74 |
| ADCY4 | 1.01E-07 | -1.75 |
| LDB3 | 8.92E-07 | -1.75 |
| CPXM2 | 3.57E-05 | -1.75 |
| NDST1 | 1.05E-21 | -1.75 |
| GRK5 | 1.45E-13 | -1.75 |
| LINC01894 | 1.84E-07 | -1.75 |
| PCAT19 | 2.76E-09 | -1.76 |
| CNTNAP3P2 | 3.56E-09 | -1.76 |
| FLI1 | 5.16E-11 | -1.76 |
| CNTN1 | 5.73E-06 | -1.76 |
| RECK | 2.60E-14 | -1.76 |
| TWIST2 | 3.17E-06 | -1.76 |
| SOD3 | 1.05E-05 | -1.76 |
| STAB2 | 7.64E-08 | -1.76 |
| HSD17B11 | 3.26E-13 | -1.76 |
| LINC00517 | 5.55E-07 | -1.76 |
| LRRC4B | 1.29E-06 | -1.76 |
| FBXL7 | 4.81E-09 | -1.76 |
| CCRL2 | 9.34E-11 | -1.76 |
| ZSWIM5 | 3.59E-11 | -1.76 |
| GRK3 | 1.81E-11 | -1.76 |
| AFAP1L2 | 1.85E-12 | -1.76 |
| CCDC68 | 7.27E-06 | -1.76 |
| DMBT1 | 1.85E-03 | -1.77 |
| SLC39A6 | 1.08E-09 | -1.77 |
| LINC01091 | 3.59E-10 | -1.77 |
| LOC105375116 | 2.99E-04 | -1.77 |
| TSPAN3 | 2.14E-15 | -1.77 |
| CDKN1A | 1.19E-08 | -1.77 |
| LOC105379443 | 3.97E-06 | -1.77 |
| CDKN1C | 1.01E-07 | -1.77 |
| VSIG4 | 5.52E-08 | -1.77 |
| SYT11 | 1.01E-11 | -1.77 |
| PROS1 | 1.24E-06 | -1.77 |
| NHLRC4 | 3.48E-15 | -1.77 |
| KLF11 | 2.48E-20 | -1.77 |
| DNALI1 | 2.27E-07 | -1.77 |
| NBEA | 1.60E-10 | -1.77 |
| RUNX1T1 | 4.84E-08 | -1.77 |
| WWC2-AS2 | 1.16E-10 | -1.77 |
| STOM | 4.72E-14 | -1.77 |
| PYGM | 5.96E-11 | -1.78 |
| PLCL1 | 5.79E-11 | -1.78 |
| LINC01589 | 4.91E-09 | -1.78 |
| OTUD1 | 1.57E-21 | -1.78 |
| MIR4758 | 2.14E-11 | -1.78 |
| INSYN1 | 1.54E-07 | -1.78 |
| NKX2-8 | 2.01E-04 | -1.78 |
| TCF15 | 2.84E-06 | -1.78 |
| LINC01695 | 3.99E-06 | -1.78 |
| AOC2 | 1.59E-08 | -1.79 |
| APH1B | 4.83E-22 | -1.79 |
| EGR2 | 1.23E-08 | -1.79 |
| RHOXF1 | 9.53E-08 | -1.79 |
| PC | 2.83E-12 | -1.79 |
| SOX5 | 1.45E-06 | -1.79 |
| CDH8 | 8.23E-08 | -1.79 |
| GPRC5B | 6.85E-18 | -1.79 |
| HMCN1 | 3.85E-07 | -1.79 |
| PDE3A | 7.99E-07 | -1.79 |
| CITED2 | 4.05E-12 | -1.79 |
| TRIM68 | 9.51E-11 | -1.79 |
| ITPK1 | 1.14E-13 | -1.79 |
| LY75-CD302 | 2.00E-21 | -1.79 |
| COLEC12 | 8.82E-08 | -1.79 |
| SERPINF1 | 2.46E-09 | -1.79 |
| ANKRD33B | 5.88E-08 | -1.80 |
| INS-IGF2 | 5.23E-06 | -1.80 |
| GIMAP1-GIMAP5 | 1.53E-08 | -1.80 |
| MAP3K20 | 1.86E-20 | -1.80 |
| NEDD9 | 1.39E-10 | -1.80 |
| MIR27A | 3.45E-06 | -1.80 |
| KIF17 | 1.38E-13 | -1.80 |
| ALDH6A1 | 1.73E-27 | -1.80 |
| KAT2B | 4.45E-16 | -1.80 |
| JUN | 8.53E-21 | -1.80 |
| CDH20 | 8.53E-06 | -1.80 |
| CALCR | 1.89E-03 | -1.81 |
| TPRG1-AS1 | 8.20E-11 | -1.81 |
| FAM107A | 1.37E-04 | -1.81 |
| CYSLTR1 | 4.13E-07 | -1.81 |
| FAXDC2 | 1.28E-25 | -1.81 |
| BOK | 2.18E-09 | -1.81 |
| ATF3 | 1.92E-12 | -1.81 |
| BMERB1 | 8.80E-12 | -1.81 |
| TCN2 | 6.14E-12 | -1.81 |
| GABARAPL1 | 3.09E-16 | -1.81 |
| VIM-AS1 | 2.04E-11 | -1.81 |
| COX4I2 | 3.99E-08 | -1.81 |
| CACHD1 | 2.86E-10 | -1.81 |
| RRAD | 2.79E-05 | -1.81 |
| ENPEP | 9.21E-08 | -1.82 |
| CCBE1 | 4.88E-10 | -1.82 |
| RHBDL3 | 9.61E-08 | -1.82 |
| IGF2 | 4.03E-06 | -1.82 |
| ANO2 | 2.19E-09 | -1.82 |
| LRP1B | 1.93E-05 | -1.82 |
| FITM2 | 6.46E-17 | -1.82 |
| KCND2 | 3.11E-06 | -1.82 |
| HNMT | 1.02E-20 | -1.82 |
| CTSO | 5.90E-18 | -1.82 |
| ELOVL5 | 6.82E-14 | -1.82 |
| VEGFC | 3.94E-11 | -1.82 |
| CD99L2 | 6.81E-20 | -1.83 |
| METTL24 | 3.25E-08 | -1.83 |
| CRYBG3 | 1.40E-24 | -1.83 |
| SLC8A1 | 8.21E-15 | -1.83 |
| CADM3-AS1 | 3.29E-05 | -1.83 |
| CADM3 | 6.57E-05 | -1.83 |
| FSTL1 | 1.24E-07 | -1.83 |
| ENTREP1 | 1.24E-08 | -1.83 |
| SERPINA1 | 4.16E-06 | -1.83 |
| MYLK | 2.05E-08 | -1.83 |
| AMIGO1 | 2.03E-21 | -1.84 |
| PDE5A | 3.89E-21 | -1.84 |
| NOVA2 | 1.69E-09 | -1.84 |
| VIPR1 | 3.50E-10 | -1.84 |
| WNT11 | 8.07E-06 | -1.84 |
| ACOT2 | 5.43E-15 | -1.84 |
| FAM124B | 2.02E-07 | -1.84 |
| TRIL | 5.75E-07 | -1.84 |
| KITLG | 8.74E-11 | -1.84 |
| PDGFRA | 7.35E-10 | -1.84 |
| ANKRD40 | 2.86E-22 | -1.84 |
| MIR23A | 7.85E-07 | -1.84 |
| PDZRN3 | 4.64E-13 | -1.84 |
| SOCS2-AS1 | 1.67E-10 | -1.85 |
| CETP | 2.04E-07 | -1.85 |
| PIK3R1 | 1.21E-12 | -1.85 |
| AFF1 | 4.59E-21 | -1.85 |
| DENND2A | 1.17E-08 | -1.85 |
| CRYL1 | 4.74E-19 | -1.85 |
| BMP3 | 1.23E-07 | -1.85 |
| SMOC2 | 5.33E-08 | -1.85 |
| DHH | 5.86E-07 | -1.85 |
| STEAP2 | 6.16E-13 | -1.85 |
| ALDH3A1 | 3.26E-07 | -1.85 |
| STXBP1 | 1.82E-12 | -1.85 |
| BMP2 | 3.10E-05 | -1.86 |
| DPYD | 1.16E-14 | -1.86 |
| GAS2L2 | 1.73E-04 | -1.86 |
| ERICH4 | 3.03E-07 | -1.86 |
| ITGA8 | 4.29E-06 | -1.86 |
| PTPRS | 2.75E-13 | -1.86 |
| LAMB2 | 1.79E-23 | -1.86 |
| HOXD1 | 2.48E-08 | -1.86 |
| MEST | 2.79E-06 | -1.86 |
| MAPT | 3.16E-07 | -1.86 |
| WFS1 | 2.96E-18 | -1.87 |
| PLD5 | 6.53E-08 | -1.87 |
| FGF14-AS2 | 6.73E-10 | -1.87 |
| DNAJC18 | 2.18E-25 | -1.87 |
| DPYSL2 | 2.01E-11 | -1.87 |
| ADCY1 | 1.07E-10 | -1.87 |
| NRN1 | 1.85E-06 | -1.87 |
| MIR1287 | 2.14E-10 | -1.87 |
| XG | 1.93E-06 | -1.87 |
| C1QTNF1 | 1.04E-10 | -1.87 |
| PHLDB2 | 8.42E-09 | -1.88 |
| USHBP1 | 9.42E-09 | -1.88 |
| CDC42EP2 | 4.18E-17 | -1.88 |
| SLC5A1 | 3.45E-05 | -1.88 |
| ARHGAP23 | 4.50E-18 | -1.88 |
| KLHDC8B | 3.60E-20 | -1.88 |
| MAST4 | 3.98E-13 | -1.88 |
| LINC01852 | 1.16E-16 | -1.88 |
| CCND2-AS1 | 1.12E-11 | -1.88 |
| ABCC6P1 | 4.02E-05 | -1.88 |
| MSX1 | 6.65E-15 | -1.88 |
| MIR22HG | 8.17E-14 | -1.89 |
| NOS3 | 1.21E-11 | -1.89 |
| ARHGAP31 | 3.10E-18 | -1.89 |
| PTGIS | 4.88E-06 | -1.89 |
| LINC00702 | 2.41E-06 | -1.89 |
| DAAM2 | 1.20E-10 | -1.89 |
| PID1 | 7.79E-07 | -1.89 |
| TYRO3 | 1.12E-09 | -1.89 |
| ABLIM1 | 4.48E-19 | -1.89 |
| CDC14C | 3.92E-14 | -1.89 |
| PDGFC | 5.24E-11 | -1.89 |
| RFX2 | 1.54E-13 | -1.89 |
| STAT5A | 9.10E-14 | -1.89 |
| UPK3A | 3.63E-05 | -1.89 |
| LIPE-AS1 | 1.87E-20 | -1.90 |
| MN1 | 9.14E-08 | -1.90 |
| RAB30 | 7.45E-18 | -1.90 |
| ACADSB | 1.52E-09 | -1.90 |
| EPHB1 | 6.33E-08 | -1.90 |
| NOSTRIN | 4.76E-16 | -1.90 |
| TCEAL5 | 1.91E-08 | -1.90 |
| PLLP | 1.69E-11 | -1.90 |
| MIR6746 | 1.86E-09 | -1.90 |
| NR4A1 | 9.34E-10 | -1.90 |
| TIPARP | 2.14E-24 | -1.90 |
| PRG4 | 2.86E-10 | -1.90 |
| DNMBP-AS1 | 7.24E-14 | -1.90 |
| ALX4 | 3.61E-05 | -1.91 |
| SYT9 | 4.28E-06 | -1.91 |
| RPH3AL | 6.55E-10 | -1.91 |
| CYP4Z2P | 4.26E-04 | -1.91 |
| RAMP2-AS1 | 6.40E-11 | -1.91 |
| PRDM16 | 5.24E-05 | -1.91 |
| ZNF728 | 1.64E-08 | -1.91 |
| SERINC1 | 1.58E-18 | -1.92 |
| CTTNBP2 | 2.50E-06 | -1.92 |
| AXL | 8.81E-18 | -1.92 |
| TK2 | 2.78E-24 | -1.92 |
| DIXDC1 | 4.55E-18 | -1.92 |
| SNX31 | 1.02E-08 | -1.92 |
| LINC02600 | 5.19E-06 | -1.92 |
| FAM89A | 7.97E-09 | -1.92 |
| TMCC3 | 5.03E-11 | -1.92 |
| ESYT1 | 6.64E-22 | -1.92 |
| CABP1 | 9.41E-10 | -1.92 |
| NLGN1 | 7.09E-06 | -1.93 |
| NEURL1B | 1.89E-11 | -1.93 |
| KLHL29 | 1.12E-11 | -1.93 |
| PLA2G2A | 7.15E-04 | -1.93 |
| BTD | 9.02E-25 | -1.93 |
| LIMS2 | 2.36E-11 | -1.93 |
| RANBP3L | 5.27E-10 | -1.93 |
| PDGFRB | 4.35E-10 | -1.94 |
| CDON | 1.21E-18 | -1.94 |
| CD302 | 2.76E-17 | -1.94 |
| FAH | 3.33E-12 | -1.94 |
| ZFPM2 | 1.07E-10 | -1.94 |
| PLAT | 5.63E-09 | -1.94 |
| ABCA1 | 1.65E-21 | -1.94 |
| ST6GALNAC6 | 2.30E-20 | -1.94 |
| SLC10A6 | 1.03E-05 | -1.94 |
| IGSF21 | 2.30E-09 | -1.94 |
| FREM1 | 5.62E-05 | -1.94 |
| ARHGAP44-AS1 | 9.04E-07 | -1.95 |
| LINC00710 | 1.82E-05 | -1.95 |
| SGK2 | 2.20E-06 | -1.95 |
| NTRK2 | 1.79E-04 | -1.95 |
| MRAS | 3.73E-15 | -1.95 |
| KCNK3 | 4.49E-06 | -1.95 |
| FRMPD2 | 1.89E-04 | -1.95 |
| LOC286297 | 4.83E-07 | -1.95 |
| LRFN5-DT | 8.59E-09 | -1.95 |
| MEIS3P1 | 5.37E-15 | -1.95 |
| AIFM2 | 2.40E-14 | -1.96 |
| MECOM | 1.39E-09 | -1.96 |
| MIR24-2 | 2.74E-08 | -1.96 |
| RILP | 1.19E-15 | -1.96 |
| KLHL30 | 1.18E-04 | -1.96 |
| WNT9B | 2.78E-08 | -1.96 |
| PTH1R | 1.91E-07 | -1.96 |
| CLEC1A | 2.24E-16 | -1.96 |
| THSD1 | 1.17E-18 | -1.96 |
| TCP11L2 | 4.69E-19 | -1.96 |
| AFAP1L1 | 1.04E-12 | -1.96 |
| PIR-FIGF | 3.63E-12 | -1.97 |
| NECAB1 | 1.16E-09 | -1.97 |
| PARVA | 2.35E-22 | -1.97 |
| PDE1C | 2.75E-08 | -1.97 |
| LINC02881 | 5.44E-13 | -1.97 |
| KANK2 | 1.89E-14 | -1.97 |
| RCAN2 | 2.12E-07 | -1.97 |
| CYP4X1 | 3.67E-05 | -1.97 |
| PRKN | 3.87E-15 | -1.97 |
| ASPH | 1.02E-13 | -1.97 |
| ITGB3 | 3.70E-09 | -1.98 |
| CNTN6 | 2.43E-08 | -1.98 |
| PALM2AKAP2 | 1.28E-12 | -1.98 |
| RASGRF2-AS1 | 5.38E-10 | -1.98 |
| TSHZ2 | 6.97E-11 | -1.98 |
| RHOXF1-AS1 | 3.14E-05 | -1.98 |
| ARHGEF40 | 1.80E-21 | -1.98 |
| TMEM26-AS1 | 8.29E-09 | -1.98 |
| TNNT3 | 4.26E-06 | -1.98 |
| SNCA | 2.97E-14 | -1.98 |
| PKDCC | 2.47E-08 | -1.99 |
| RAMP3 | 6.92E-11 | -1.99 |
| TMEM47 | 9.78E-14 | -1.99 |
| TINAGL1 | 4.81E-18 | -1.99 |
| ABCA10 | 8.02E-08 | -1.99 |
| SLC22A11 | 1.15E-05 | -1.99 |
| CH25H | 9.04E-10 | -1.99 |
| CYTL1 | 2.53E-07 | -1.99 |
| LAMA4 | 5.42E-10 | -1.99 |
| DUSP4 | 1.54E-09 | -1.99 |
| SYNM | 1.00E-07 | -1.99 |
| PER1 | 1.33E-12 | -1.99 |
| SPON1 | 2.88E-11 | -1.99 |
| GIMAP1 | 1.34E-10 | -1.99 |
| OR51E1 | 8.48E-06 | -2.00 |
| INHBB | 9.79E-09 | -2.00 |
| TNS2 | 3.66E-13 | -2.00 |
| LINC01140 | 3.67E-11 | -2.00 |
| MSRB3 | 2.30E-12 | -2.00 |
| FGF7 | 8.11E-11 | -2.00 |
| RTN1 | 1.06E-12 | -2.00 |
| MYLK-AS1 | 2.44E-18 | -2.00 |
| SRL | 1.84E-07 | -2.00 |
| CLMAT3 | 2.45E-09 | -2.00 |
| MAN1C1 | 4.56E-12 | -2.01 |
| MID2 | 4.35E-30 | -2.01 |
| OLFML1 | 1.43E-11 | -2.01 |
| ZBTB4 | 6.05E-34 | -2.01 |
| TSKU | 7.42E-11 | -2.01 |
| C11orf96 | 9.19E-09 | -2.01 |
| DSCAM | 1.20E-05 | -2.01 |
| BDKRB2 | 1.48E-11 | -2.01 |
| CA12 | 3.73E-07 | -2.01 |
| FMO2 | 1.20E-07 | -2.02 |
| ACSS3 | 1.36E-09 | -2.02 |
| LPAR1 | 1.41E-12 | -2.02 |
| ANKFN1 | 3.74E-07 | -2.02 |
| LINC00840 | 7.98E-07 | -2.02 |
| GAS7 | 8.64E-14 | -2.02 |
| PCDH12 | 3.06E-13 | -2.02 |
| EMP1 | 1.13E-08 | -2.02 |
| AR | 3.79E-06 | -2.02 |
| RTL5 | 1.15E-20 | -2.02 |
| C3 | 2.70E-10 | -2.02 |
| NEK10 | 8.83E-06 | -2.02 |
| SLC7A14-AS1 | 1.37E-06 | -2.02 |
| SHROOM4 | 3.42E-15 | -2.02 |
| C2CD2 | 1.10E-16 | -2.02 |
| SETD7 | 3.87E-17 | -2.03 |
| TBX5-AS1 | 3.36E-10 | -2.03 |
| ADGRL3 | 7.83E-05 | -2.03 |
| FLT1 | 4.13E-14 | -2.03 |
| PTPRG | 6.83E-19 | -2.03 |
| CDH22 | 1.59E-06 | -2.03 |
| NID1 | 1.85E-10 | -2.03 |
| PLPP7 | 1.87E-12 | -2.03 |
| ABTB3 | 1.65E-11 | -2.04 |
| CPD | 2.67E-18 | -2.04 |
| RAMP2 | 6.35E-12 | -2.04 |
| PRDM16-DT | 1.41E-05 | -2.04 |
| WASF3 | 1.99E-13 | -2.04 |
| PLVAP | 1.43E-11 | -2.04 |
| CCDC8 | 1.81E-08 | -2.04 |
| ZEB2 | 2.78E-14 | -2.04 |
| TACC1 | 2.87E-17 | -2.04 |
| CSRNP3 | 3.66E-11 | -2.04 |
| HEG1 | 1.40E-16 | -2.05 |
| CLEC4GP1 | 3.33E-06 | -2.05 |
| MIR6084 | 1.66E-15 | -2.05 |
| GNAT1 | 1.87E-05 | -2.05 |
| RHOU | 1.61E-14 | -2.05 |
| INKA2 | 9.93E-17 | -2.05 |
| PGLYRP2 | 1.63E-05 | -2.06 |
| PDE8B | 1.00E-16 | -2.06 |
| LINC01673 | 1.98E-06 | -2.06 |
| ALDOC | 1.31E-09 | -2.06 |
| LAMC1 | 1.49E-12 | -2.06 |
| SIGLEC17P | 3.00E-09 | -2.06 |
| HOXD8 | 2.60E-14 | -2.06 |
| ANK2 | 5.66E-09 | -2.06 |
| CYP2U1 | 1.05E-24 | -2.06 |
| LINC00654 | 3.49E-11 | -2.06 |
| CRTAP | 8.79E-20 | -2.06 |
| IGFBP4 | 3.41E-13 | -2.06 |
| ITGA1 | 9.89E-16 | -2.07 |
| FAM47E | 1.05E-13 | -2.07 |
| ABCC8 | 2.32E-04 | -2.07 |
| TP53AIP1 | 1.52E-09 | -2.07 |
| NOTCH4 | 6.32E-13 | -2.07 |
| MRGPRF-AS1 | 5.62E-07 | -2.07 |
| SASH1 | 2.27E-21 | -2.07 |
| FERMT2 | 7.37E-15 | -2.07 |
| JAM3 | 1.74E-23 | -2.07 |
| ASAH1 | 7.10E-17 | -2.07 |
| CRY2 | 4.68E-20 | -2.08 |
| CBX7 | 1.74E-12 | -2.08 |
| MT1A | 4.96E-10 | -2.08 |
| HECTD2-AS1 | 2.78E-06 | -2.08 |
| LINC00841 | 1.35E-07 | -2.08 |
| LINC00639 | 4.53E-06 | -2.08 |
| EMCN | 3.66E-10 | -2.09 |
| SCUBE1 | 2.90E-05 | -2.09 |
| INPP4B | 9.81E-12 | -2.09 |
| IGDCC3 | 2.76E-05 | -2.09 |
| GPBAR1 | 5.47E-09 | -2.09 |
| EGFLAM | 2.37E-09 | -2.09 |
| GRIK1-AS1 | 2.45E-06 | -2.09 |
| GJA4 | 9.11E-10 | -2.09 |
| HIF3A | 6.46E-07 | -2.09 |
| RASSF9 | 2.38E-12 | -2.10 |
| SCARF1 | 1.06E-17 | -2.10 |
| B3GALT1 | 1.27E-08 | -2.10 |
| SV2B | 3.25E-06 | -2.10 |
| STK32B | 2.54E-15 | -2.10 |
| CUX2 | 4.51E-07 | -2.10 |
| LRP10 | 4.82E-21 | -2.10 |
| CYP11A1 | 2.30E-10 | -2.10 |
| FOXO1 | 7.38E-18 | -2.10 |
| IL1RL1 | 1.18E-07 | -2.10 |
| GCNT4 | 7.32E-11 | -2.10 |
| LRRC70 | 2.15E-23 | -2.10 |
| MAOB | 1.70E-07 | -2.10 |
| DPYD-AS1 | 9.10E-24 | -2.10 |
| EPS8 | 3.58E-20 | -2.11 |
| LRRC2 | 9.67E-11 | -2.11 |
| TTC28 | 1.90E-28 | -2.11 |
| CKMT2 | 8.00E-20 | -2.11 |
| SLC16A2 | 4.52E-15 | -2.11 |
| RBMS3-AS3 | 4.18E-08 | -2.11 |
| SCN11A | 3.20E-11 | -2.11 |
| KCNA2 | 1.38E-05 | -2.11 |
| SLC4A1 | 9.99E-07 | -2.11 |
| KRT222 | 2.21E-08 | -2.12 |
| WLS | 6.29E-11 | -2.12 |
| KCNK15 | 3.62E-09 | -2.12 |
| FAM43A | 4.33E-13 | -2.12 |
| SLC29A4 | 1.61E-07 | -2.12 |
| PTPRM | 4.45E-14 | -2.12 |
| FBLN1 | 1.85E-10 | -2.13 |
| NXPH3 | 3.89E-10 | -2.13 |
| RBPMS2 | 8.49E-10 | -2.13 |
| IL20RA | 3.41E-08 | -2.13 |
| MYL7 | 2.95E-04 | -2.13 |
| CD248 | 1.68E-07 | -2.13 |
| GATA2 | 6.26E-09 | -2.13 |
| EEPD1 | 2.00E-22 | -2.13 |
| PTGER4 | 4.37E-21 | -2.13 |
| DEPP1 | 5.55E-13 | -2.14 |
| PELI2 | 1.32E-14 | -2.14 |
| GALNT16 | 5.54E-10 | -2.14 |
| PFKFB1 | 5.07E-05 | -2.14 |
| AFF3 | 1.53E-06 | -2.14 |
| SEMA3C | 1.29E-09 | -2.14 |
| CALML3 | 5.59E-07 | -2.14 |
| PLPP1 | 3.50E-19 | -2.14 |
| IRAK3 | 9.37E-18 | -2.15 |
| ST6GALNAC1 | 4.18E-11 | -2.15 |
| C6 | 6.37E-05 | -2.15 |
| LINC00377 | 2.90E-06 | -2.15 |
| PPP1R3C | 4.53E-09 | -2.15 |
| SALL2 | 2.53E-14 | -2.15 |
| AJAP1 | 8.11E-16 | -2.16 |
| NRP1 | 2.17E-17 | -2.16 |
| COBLL1 | 1.07E-23 | -2.16 |
| AGAP11 | 4.32E-14 | -2.16 |
| DLGAP2 | 7.65E-12 | -2.16 |
| SLC14A2-AS1 | 3.25E-08 | -2.16 |
| ZNF219 | 4.38E-25 | -2.16 |
| TDRD1 | 6.79E-11 | -2.16 |
| PKD1L2 | 5.00E-07 | -2.16 |
| LETR1 | 5.51E-10 | -2.17 |
| TMEM170B | 2.04E-14 | -2.17 |
| CLEC4G | 1.48E-05 | -2.17 |
| FAM13C | 9.16E-13 | -2.17 |
| TSLP | 2.17E-10 | -2.17 |
| CAVIN1 | 8.01E-15 | -2.18 |
| LINC02185 | 4.02E-10 | -2.18 |
| ZFHX4-AS1 | 1.06E-08 | -2.19 |
| F10 | 5.34E-10 | -2.19 |
| PTCHD4 | 2.74E-11 | -2.19 |
| GREM2 | 6.15E-07 | -2.19 |
| ABCB1 | 7.86E-12 | -2.19 |
| TACR1 | 8.82E-10 | -2.19 |
| ABCA6 | 2.09E-10 | -2.19 |
| DUSP6 | 1.59E-20 | -2.19 |
| S1PR1-DT | 3.58E-11 | -2.19 |
| CYGB | 2.26E-12 | -2.19 |
| ZNF385D | 1.57E-13 | -2.20 |
| TF | 3.18E-08 | -2.20 |
| TPPP3 | 1.08E-11 | -2.20 |
| RASD1 | 1.52E-10 | -2.20 |
| ACO1 | 2.01E-21 | -2.20 |
| TMEM273 | 2.21E-13 | -2.20 |
| FXYD1 | 5.03E-08 | -2.20 |
| MIR198 | 1.09E-08 | -2.20 |
| MAGI2-AS3 | 5.24E-17 | -2.20 |
| RBP7 | 6.26E-10 | -2.20 |
| MIR143 | 3.84E-06 | -2.21 |
| ARHGEF6 | 1.52E-17 | -2.21 |
| GPD1L | 2.38E-24 | -2.21 |
| LINC00504 | 1.26E-09 | -2.21 |
| NR4A3 | 1.29E-14 | -2.21 |
| LYPD6 | 2.15E-07 | -2.21 |
| CRTAC1 | 1.37E-05 | -2.21 |
| PTPRT | 1.77E-06 | -2.21 |
| RSPO3 | 3.54E-12 | -2.21 |
| FEZ1 | 1.34E-21 | -2.21 |
| CCND2 | 5.98E-15 | -2.22 |
| MIR2277 | 3.61E-20 | -2.22 |
| NSG1 | 4.12E-10 | -2.22 |
| ARHGAP6 | 4.44E-13 | -2.22 |
| GJB1 | 8.49E-05 | -2.22 |
| SLC22A3 | 1.70E-09 | -2.22 |
| PLPPR1 | 3.21E-06 | -2.22 |
| LINC01801 | 1.10E-09 | -2.22 |
| CAB39L | 1.05E-32 | -2.22 |
| BOK-AS1 | 3.57E-11 | -2.23 |
| PLAAT3 | 1.34E-09 | -2.23 |
| SLC12A2 | 6.52E-14 | -2.23 |
| ECSCR | 5.85E-16 | -2.23 |
| PDE1A | 9.89E-14 | -2.23 |
| GPRASP2 | 1.18E-34 | -2.23 |
| LRIG1 | 3.01E-23 | -2.23 |
| EPHA3 | 1.42E-07 | -2.24 |
| SYPL2 | 3.98E-14 | -2.24 |
| OLFML2A | 3.01E-11 | -2.24 |
| IGSF1 | 3.01E-06 | -2.25 |
| TEF | 3.34E-20 | -2.25 |
| MIR1182 | 2.42E-11 | -2.25 |
| GNG2 | 2.83E-12 | -2.25 |
| TFPI | 3.11E-15 | -2.25 |
| FRY | 2.56E-25 | -2.25 |
| ACSL1 | 5.56E-10 | -2.25 |
| LHX6 | 1.62E-09 | -2.25 |
| MRC1 | 1.35E-08 | -2.25 |
| PCSK5 | 1.36E-12 | -2.26 |
| FGB | 8.31E-04 | -2.26 |
| CNRIP1 | 3.72E-16 | -2.26 |
| KLF9 | 1.66E-20 | -2.26 |
| ANO1 | 3.05E-17 | -2.26 |
| ATP1B2 | 1.17E-10 | -2.26 |
| MIR6852 | 1.81E-15 | -2.27 |
| ELAPOR2 | 1.19E-12 | -2.27 |
| HSPG2 | 1.22E-16 | -2.27 |
| CDH6 | 1.01E-10 | -2.27 |
| LINC02747 | 4.59E-05 | -2.27 |
| TENM3-AS1 | 8.12E-10 | -2.27 |
| RASGRF2 | 5.82E-16 | -2.27 |
| C8orf88 | 2.84E-14 | -2.27 |
| TRABD2B | 2.70E-08 | -2.27 |
| KAAG1 | 3.71E-06 | -2.27 |
| GPR88 | 4.05E-11 | -2.27 |
| STC2 | 2.30E-09 | -2.27 |
| BCL6B | 2.78E-14 | -2.28 |
| LINC02228 | 2.42E-16 | -2.28 |
| VSIR | 1.56E-20 | -2.28 |
| SOX18 | 1.11E-11 | -2.28 |
| LUARIS | 3.17E-10 | -2.28 |
| PODN | 4.68E-09 | -2.28 |
| JCAD | 9.82E-13 | -2.28 |
| EIF4E3 | 1.10E-21 | -2.28 |
| SPATA18 | 1.44E-16 | -2.28 |
| RERGL | 2.39E-04 | -2.29 |
| EGF | 1.16E-06 | -2.29 |
| NR3C2 | 1.55E-23 | -2.29 |
| CCL16 | 3.98E-11 | -2.29 |
| DTX1 | 8.70E-10 | -2.29 |
| PLN | 3.04E-08 | -2.29 |
| CCL28 | 9.31E-07 | -2.29 |
| LINC02660 | 1.57E-07 | -2.30 |
| C16orf89 | 5.89E-09 | -2.30 |
| LINC00924 | 2.30E-08 | -2.30 |
| AMOTL2 | 7.02E-19 | -2.30 |
| ADRA2C | 1.39E-08 | -2.30 |
| IL1R1 | 2.39E-28 | -2.30 |
| ADRA1D | 1.37E-09 | -2.31 |
| ARHGAP36 | 1.06E-03 | -2.31 |
| KCND3 | 2.38E-09 | -2.31 |
| SYTL5 | 5.75E-06 | -2.31 |
| ARRB1 | 3.22E-32 | -2.31 |
| PRICKLE2 | 2.65E-16 | -2.31 |
| SPRY2 | 7.94E-18 | -2.31 |
| ADAMTS9-AS2 | 1.08E-09 | -2.31 |
| SELE | 5.37E-08 | -2.31 |
| MCAM | 3.68E-16 | -2.32 |
| DPP4 | 1.27E-11 | -2.32 |
| TRPC6 | 4.58E-30 | -2.32 |
| ACVRL1 | 1.80E-18 | -2.32 |
| HMGCS2 | 5.95E-03 | -2.33 |
| CHST7 | 2.35E-31 | -2.33 |
| MYO16 | 1.23E-07 | -2.33 |
| THSD4 | 6.25E-10 | -2.33 |
| HPSE2 | 2.80E-06 | -2.34 |
| TIMP3 | 1.93E-09 | -2.34 |
| VSTM4 | 3.26E-13 | -2.34 |
| VIPR1-AS1 | 2.25E-15 | -2.34 |
| FOLR2 | 3.70E-10 | -2.34 |
| SLC47A1 | 1.04E-25 | -2.34 |
| PLSCR4 | 7.51E-22 | -2.34 |
| NMNAT2 | 9.28E-10 | -2.34 |
| RIMBP2 | 3.23E-14 | -2.35 |
| NOVA1 | 1.51E-08 | -2.35 |
| NECTIN3 | 1.05E-17 | -2.35 |
| GNS | 9.93E-21 | -2.35 |
| KLHL4 | 1.60E-11 | -2.35 |
| SIK2 | 1.49E-19 | -2.35 |
| NTF3 | 1.14E-09 | -2.36 |
| AGMO | 2.33E-11 | -2.36 |
| RHOB | 3.10E-22 | -2.36 |
| STARD8 | 1.63E-20 | -2.36 |
| CAPN11 | 5.15E-17 | -2.36 |
| PDZD2 | 2.07E-15 | -2.36 |
| F8 | 9.34E-29 | -2.37 |
| TAL1 | 2.85E-13 | -2.37 |
| ZFP36 | 3.45E-19 | -2.37 |
| SFRP4 | 3.75E-08 | -2.37 |
| CCDC170 | 2.17E-15 | -2.37 |
| GCOM1 | 4.49E-36 | -2.37 |
| AMIGO2 | 5.29E-15 | -2.37 |
| ADAM33 | 2.77E-09 | -2.37 |
| HGF | 2.04E-19 | -2.37 |
| EDA2R | 1.68E-18 | -2.37 |
| PHYHIP | 5.74E-12 | -2.38 |
| GFRA1 | 3.83E-05 | -2.38 |
| KIT | 1.11E-07 | -2.38 |
| SEMA6B | 2.30E-17 | -2.38 |
| GNAI1 | 1.86E-17 | -2.38 |
| PDE2A-AS2 | 6.33E-13 | -2.38 |
| CXCL2 | 1.27E-10 | -2.38 |
| DKK3 | 4.33E-13 | -2.38 |
| MYH1 | 2.59E-09 | -2.38 |
| TNFRSF10C | 2.52E-25 | -2.39 |
| RASL11A | 1.47E-14 | -2.39 |
| STBD1 | 4.72E-17 | -2.39 |
| RGS22 | 2.04E-11 | -2.39 |
| THRB | 5.02E-20 | -2.40 |
| PRRG3 | 3.13E-09 | -2.40 |
| NAP1L2 | 6.90E-10 | -2.41 |
| ACOX2 | 1.30E-13 | -2.41 |
| LOC102724904 | 1.13E-09 | -2.41 |
| OLFM4 | 1.65E-04 | -2.41 |
| ANTXR2 | 2.03E-22 | -2.41 |
| EBF1 | 2.64E-15 | -2.41 |
| HSPA12A | 1.10E-14 | -2.41 |
| MIR145 | 1.54E-08 | -2.42 |
| LHFPL6 | 3.68E-17 | -2.42 |
| PLCXD3 | 3.40E-06 | -2.42 |
| MOCS1 | 8.66E-24 | -2.42 |
| EBF3 | 1.01E-13 | -2.42 |
| FGL2 | 1.61E-19 | -2.42 |
| ERG | 4.44E-18 | -2.42 |
| CPE | 3.16E-13 | -2.42 |
| PLIN5 | 4.51E-08 | -2.43 |
| LDHD | 7.65E-14 | -2.43 |
| UBXN10 | 1.48E-11 | -2.43 |
| SLC40A1 | 7.82E-11 | -2.43 |
| RASIP1 | 6.23E-22 | -2.43 |
| PLPPR4 | 1.78E-14 | -2.43 |
| C2CD4C | 1.79E-12 | -2.43 |
| LILRB5 | 2.04E-11 | -2.43 |
| DCLK1 | 1.62E-14 | -2.43 |
| LOC101927366 | 8.37E-06 | -2.43 |
| XPNPEP2 | 3.51E-06 | -2.43 |
| CFH | 5.59E-18 | -2.43 |
| LRP2 | 8.76E-06 | -2.43 |
| NUAK1 | 5.81E-21 | -2.43 |
| SYBU | 4.36E-14 | -2.44 |
| TPRG1-AS2 | 1.72E-18 | -2.44 |
| TPPP | 1.08E-19 | -2.44 |
| LCN6 | 9.52E-08 | -2.44 |
| ALDH4A1 | 5.35E-16 | -2.44 |
| KLHL13 | 7.52E-13 | -2.44 |
| LRFN5 | 4.56E-14 | -2.44 |
| PNPLA2 | 1.45E-19 | -2.44 |
| PPP1R16B | 1.88E-10 | -2.44 |
| FGD5 | 5.37E-14 | -2.45 |
| ABCC6 | 8.37E-10 | -2.45 |
| ITPR1 | 5.25E-23 | -2.45 |
| CAT | 2.13E-23 | -2.45 |
| TFF1 | 8.39E-03 | -2.45 |
| PFKFB3 | 2.41E-19 | -2.45 |
| ACER2 | 1.16E-32 | -2.45 |
| LRRC32 | 5.22E-13 | -2.45 |
| LTBP2 | 1.19E-21 | -2.46 |
| ADGRA2 | 2.43E-16 | -2.46 |
| WSCD2 | 4.71E-07 | -2.47 |
| ABHD6 | 9.91E-31 | -2.47 |
| KCNJ8 | 3.83E-18 | -2.47 |
| BRINP1 | 3.91E-07 | -2.47 |
| ISLR2 | 3.33E-14 | -2.47 |
| SHE | 1.32E-23 | -2.47 |
| P2RX6 | 1.69E-10 | -2.47 |
| DACT2 | 1.13E-08 | -2.47 |
| ESAM | 1.32E-17 | -2.48 |
| PDZRN4 | 6.95E-10 | -2.48 |
| NLGN4X | 1.20E-11 | -2.48 |
| MIR7844 | 3.63E-15 | -2.49 |
| RGS5 | 1.92E-12 | -2.49 |
| EDN3 | 3.82E-05 | -2.49 |
| NMUR1 | 4.88E-10 | -2.49 |
| TENM3 | 6.92E-13 | -2.49 |
| AHNAK | 1.66E-30 | -2.49 |
| SYN2 | 3.66E-10 | -2.49 |
| CCDC80 | 1.05E-12 | -2.50 |
| NAV3 | 3.77E-20 | -2.50 |
| FAM149A | 2.33E-15 | -2.50 |
| RBMS3 | 8.73E-17 | -2.50 |
| CXCL14 | 6.78E-09 | -2.51 |
| LMOD1 | 5.82E-11 | -2.51 |
| PDE7B | 9.24E-18 | -2.51 |
| FFAR4 | 2.30E-13 | -2.51 |
| GPER1 | 1.87E-19 | -2.51 |
| KDR | 9.11E-21 | -2.52 |
| CALHM5 | 9.90E-16 | -2.52 |
| LOC100128164 | 6.70E-12 | -2.52 |
| ANO3 | 4.26E-11 | -2.52 |
| FFAR3 | 1.57E-08 | -2.53 |
| SOCS2 | 4.19E-19 | -2.53 |
| EFEMP1 | 2.04E-09 | -2.54 |
| LINC00908 | 5.67E-25 | -2.54 |
| PGR-AS1 | 1.52E-12 | -2.54 |
| FGF2 | 2.51E-17 | -2.54 |
| LINC02343 | 8.75E-07 | -2.54 |
| CX3CL1 | 4.80E-18 | -2.55 |
| HCAR3 | 2.00E-11 | -2.55 |
| MAN1A1 | 1.58E-22 | -2.55 |
| CCM2L | 2.26E-19 | -2.55 |
| OMD | 1.64E-14 | -2.55 |
| SPRY1 | 9.97E-24 | -2.57 |
| SEMA5A | 1.56E-17 | -2.57 |
| PCDH9 | 7.65E-14 | -2.57 |
| CILP | 2.85E-10 | -2.57 |
| SAMD5 | 3.70E-13 | -2.57 |
| ELANE | 8.58E-07 | -2.58 |
| ACKR3 | 7.85E-16 | -2.58 |
| ANO6 | 7.45E-31 | -2.58 |
| HMCN2 | 3.10E-10 | -2.58 |
| LUZP2 | 6.93E-08 | -2.58 |
| BTG2 | 8.12E-25 | -2.58 |
| TFPI2 | 3.13E-07 | -2.58 |
| RNF157 | 7.25E-18 | -2.58 |
| TLR4 | 5.57E-30 | -2.58 |
| TIE1 | 3.86E-17 | -2.59 |
| DLC1 | 2.15E-18 | -2.59 |
| STX11 | 2.58E-19 | -2.60 |
| ALDH1A2-AS1 | 1.06E-08 | -2.60 |
| LINC00993 | 1.52E-03 | -2.60 |
| C1orf115 | 6.20E-18 | -2.60 |
| LOC107984355 | 4.19E-12 | -2.60 |
| PYGL | 4.26E-14 | -2.61 |
| SPAG6 | 1.57E-09 | -2.61 |
| TCEAL7 | 3.70E-17 | -2.61 |
| MLXIPL | 2.21E-08 | -2.61 |
| NATD1 | 1.62E-25 | -2.61 |
| SLC13A2 | 2.76E-04 | -2.61 |
| FGF1 | 9.16E-19 | -2.61 |
| HSPA12A-AS1 | 1.62E-14 | -2.62 |
| RNASE4 | 9.25E-22 | -2.62 |
| SCD | 8.29E-09 | -2.62 |
| FAM47E-STBD1 | 1.35E-18 | -2.62 |
| PGM5P4 | 3.05E-14 | -2.62 |
| CAVIN2-AS1 | 1.15E-08 | -2.62 |
| PPP1R1A | 1.09E-07 | -2.63 |
| GPR42 | 3.06E-08 | -2.63 |
| IRS1 | 1.50E-18 | -2.63 |
| SEMA6D | 7.26E-21 | -2.63 |
| IL6ST | 2.89E-31 | -2.63 |
| LRRC3B | 1.89E-10 | -2.63 |
| KLF15 | 9.96E-11 | -2.64 |
| TLR3 | 1.36E-22 | -2.64 |
| NPY1R | 4.59E-09 | -2.64 |
| MGARP | 2.97E-19 | -2.64 |
| SHISA6 | 4.10E-10 | -2.65 |
| SMIM10L2A | 1.11E-18 | -2.65 |
| HRC | 3.65E-11 | -2.65 |
| CORO2B | 6.92E-23 | -2.65 |
| SPARCL1 | 8.24E-25 | -2.65 |
| KANK3 | 3.25E-21 | -2.66 |
| FAT4 | 2.76E-19 | -2.66 |
| TPO | 4.51E-10 | -2.66 |
| GRIA4 | 9.33E-10 | -2.66 |
| SCGB3A1 | 8.66E-07 | -2.67 |
| SERPINA11 | 2.46E-04 | -2.67 |
| SLC7A10 | 1.73E-09 | -2.67 |
| TFF3 | 1.13E-04 | -2.67 |
| DOCK11 | 1.39E-22 | -2.67 |
| TMEM100 | 4.60E-08 | -2.67 |
| ST6GALNAC3 | 1.51E-30 | -2.67 |
| COL15A1 | 8.57E-19 | -2.67 |
| CCDC178 | 2.31E-11 | -2.67 |
| LINC00671 | 6.17E-13 | -2.67 |
| GIPC2 | 1.23E-10 | -2.68 |
| MYOM1 | 1.37E-17 | -2.68 |
| ANGPT1 | 1.21E-11 | -2.68 |
| CX3CR1 | 3.13E-12 | -2.68 |
| FBN1 | 4.86E-15 | -2.68 |
| ACADL | 6.06E-11 | -2.68 |
| SCTR | 1.95E-12 | -2.69 |
| LINC00989 | 2.65E-12 | -2.69 |
| TESC | 2.52E-17 | -2.69 |
| TBC1D9 | 2.03E-23 | -2.69 |
| ABI3BP | 2.23E-12 | -2.69 |
| GIMAP8 | 1.18E-18 | -2.69 |
| TMEM255A | 6.04E-17 | -2.69 |
| DMRT2 | 4.32E-09 | -2.70 |
| NDN | 6.23E-16 | -2.70 |
| KCNJ12 | 5.61E-14 | -2.70 |
| CSRNP1 | 1.89E-36 | -2.70 |
| KCNA5 | 1.58E-10 | -2.71 |
| BPIFB1 | 9.51E-07 | -2.71 |
| THBS4-AS1 | 3.87E-13 | -2.71 |
| ALAS2 | 1.48E-09 | -2.71 |
| ZCCHC24 | 7.44E-21 | -2.72 |
| SRARP | 3.20E-05 | -2.72 |
| WFIKKN2 | 6.92E-11 | -2.72 |
| RNU6ATAC35P | 1.74E-11 | -2.72 |
| RAPGEF3 | 2.06E-18 | -2.72 |
| SLC17A7 | 7.51E-28 | -2.72 |
| LINC01485 | 1.34E-08 | -2.72 |
| IGSF10 | 6.43E-17 | -2.72 |
| LAMA3 | 1.05E-16 | -2.73 |
| UGT2B15 | 1.30E-06 | -2.73 |
| GSN | 1.12E-23 | -2.73 |
| DCDC2 | 1.12E-07 | -2.74 |
| PARM1 | 4.32E-16 | -2.74 |
| RAI2 | 3.38E-14 | -2.74 |
| CLEC14A | 7.29E-16 | -2.75 |
| TLN2 | 4.01E-32 | -2.75 |
| NTN4 | 1.02E-13 | -2.75 |
| SOX7 | 7.43E-21 | -2.75 |
| SLC1A7 | 1.23E-15 | -2.76 |
| KLHL31 | 7.53E-16 | -2.76 |
| RGS7BP | 5.35E-11 | -2.76 |
| HAS1 | 7.93E-10 | -2.77 |
| LRRN3 | 1.27E-21 | -2.77 |
| IL6 | 3.35E-11 | -2.77 |
| DNASE1L3 | 8.36E-10 | -2.77 |
| UNC13C | 2.86E-22 | -2.77 |
| MEDAG | 2.32E-15 | -2.77 |
| SUCNR1 | 5.35E-15 | -2.77 |
| CLMP | 1.05E-20 | -2.78 |
| SHISA3 | 5.79E-09 | -2.78 |
| FLNC | 6.25E-14 | -2.79 |
| VGLL3 | 1.01E-19 | -2.79 |
| MMP28 | 1.24E-12 | -2.79 |
| SRPX | 1.49E-16 | -2.79 |
| FLNC-AS1 | 3.35E-14 | -2.79 |
| THSD7B | 8.42E-12 | -2.80 |
| SH3D19 | 1.53E-41 | -2.80 |
| EGFL6 | 5.36E-08 | -2.80 |
| SOCS3 | 1.41E-20 | -2.80 |
| KIF26A | 4.22E-14 | -2.80 |
| ZNF366 | 3.87E-22 | -2.80 |
| SEMA3D | 3.71E-15 | -2.80 |
| DDR2 | 3.66E-20 | -2.81 |
| THBD | 1.34E-18 | -2.81 |
| KCTD12 | 1.56E-20 | -2.81 |
| TLL1 | 9.88E-18 | -2.81 |
| SYNPO | 1.32E-17 | -2.81 |
| DRD1 | 2.05E-09 | -2.81 |
| SLC16A12 | 6.91E-11 | -2.82 |
| CEBPA | 3.77E-16 | -2.82 |
| EPDR1 | 6.89E-19 | -2.82 |
| AGR3 | 5.35E-06 | -2.82 |
| PENK-AS1 | 6.73E-13 | -2.82 |
| RETSAT | 6.21E-25 | -2.83 |
| KLF2 | 6.96E-19 | -2.83 |
| ARHGEF15 | 4.34E-19 | -2.83 |
| INMT | 8.64E-10 | -2.83 |
| HLF | 3.80E-15 | -2.83 |
| VIT | 1.60E-12 | -2.85 |
| ATP1A4 | 1.88E-16 | -2.85 |
| TXNIP | 1.37E-20 | -2.85 |
| ZNF423 | 1.97E-18 | -2.85 |
| PRRT4 | 2.13E-08 | -2.85 |
| PEAR1 | 1.31E-19 | -2.85 |
| CXCL12 | 2.63E-17 | -2.86 |
| CYP4F22 | 7.63E-06 | -2.86 |
| C7 | 8.08E-08 | -2.86 |
| SH3BGRL2 | 4.04E-24 | -2.86 |
| CA4 | 1.46E-40 | -2.86 |
| ROBO4 | 1.34E-21 | -2.86 |
| CAV2 | 7.21E-28 | -2.86 |
| FAM241A | 2.15E-28 | -2.86 |
| HOGA1 | 2.20E-16 | -2.86 |
| KLHDC7A | 9.64E-06 | -2.87 |
| TBX15 | 6.92E-23 | -2.87 |
| CHL1-AS1 | 4.20E-13 | -2.87 |
| GASK1B-AS1 | 9.69E-25 | -2.87 |
| ALDH1A1 | 5.88E-11 | -2.87 |
| HAS3 | 5.83E-26 | -2.88 |
| GNG11 | 1.12E-20 | -2.88 |
| FAM180B | 1.96E-12 | -2.88 |
| ITM2A | 7.63E-17 | -2.89 |
| HCAR2 | 6.86E-13 | -2.89 |
| RNF186 | 5.97E-06 | -2.89 |
| DIPK2B | 2.46E-24 | -2.89 |
| TCN1 | 1.80E-06 | -2.89 |
| CNR1 | 1.21E-11 | -2.90 |
| LINC02511 | 3.67E-07 | -2.90 |
| KLHL33 | 2.05E-14 | -2.90 |
| NGFR | 3.98E-13 | -2.90 |
| HSPB8 | 8.48E-17 | -2.91 |
| STS | 9.36E-25 | -2.91 |
| PGAP4 | 2.71E-20 | -2.91 |
| PALMD | 6.40E-20 | -2.92 |
| ADAMTS1 | 6.75E-27 | -2.92 |
| PAK5 | 3.80E-10 | -2.92 |
| MTARC1 | 6.13E-14 | -2.92 |
| ARHGEF7-AS2 | 1.29E-12 | -2.93 |
| ABCG2 | 3.05E-19 | -2.93 |
| PEBP4 | 1.81E-15 | -2.93 |
| NR5A2 | 9.71E-27 | -2.93 |
| SPTBN1 | 1.02E-30 | -2.94 |
| DES | 6.13E-07 | -2.95 |
| EGR3 | 4.18E-15 | -2.95 |
| PRKAR2B | 2.77E-18 | -2.95 |
| DMGDH | 2.75E-20 | -2.95 |
| P2RY12 | 1.60E-13 | -2.95 |
| RNASE7 | 4.08E-12 | -2.96 |
| DOC2B | 5.91E-18 | -2.96 |
| MUCL1 | 3.23E-04 | -2.96 |
| ANKRD29 | 2.80E-17 | -2.96 |
| CCDC152 | 1.72E-22 | -2.96 |
| MFAP4 | 3.01E-12 | -2.96 |
| CDH13 | 4.71E-26 | -2.97 |
| ANGPTL7 | 5.43E-17 | -2.97 |
| IL1RAPL2 | 4.82E-11 | -2.97 |
| IGF1 | 5.70E-15 | -2.97 |
| PTPRB | 9.47E-22 | -2.97 |
| SLC7A4 | 9.35E-08 | -2.98 |
| PAPPA2 | 1.06E-10 | -2.98 |
| TAC1 | 2.77E-08 | -2.98 |
| LOC105373989 | 2.55E-15 | -2.98 |
| MTURN | 8.18E-28 | -2.99 |
| SLC7A2 | 5.43E-17 | -2.99 |
| AKAP12 | 1.90E-20 | -2.99 |
| ADAMTS18 | 3.04E-12 | -2.99 |
| LRRC58-DT | 1.47E-16 | -2.99 |
| KCNE1 | 3.07E-19 | -2.99 |
| MIR22 | 5.14E-23 | -3.00 |
| F13A1 | 5.10E-12 | -3.00 |
| NPY2R | 8.86E-04 | -3.00 |
| CADM2 | 1.95E-13 | -3.00 |
| GSTM5 | 8.54E-16 | -3.00 |
| ALDH2 | 8.28E-21 | -3.01 |
| HSPA12B | 1.03E-25 | -3.01 |
| SLC16A7 | 2.54E-21 | -3.01 |
| EMX2OS | 2.20E-16 | -3.02 |
| CPA3 | 5.72E-12 | -3.02 |
| LINC02202 | 1.45E-19 | -3.02 |
| SCUBE2 | 8.51E-09 | -3.02 |
| SNCG | 8.90E-16 | -3.02 |
| COPG2IT1 | 2.18E-16 | -3.02 |
| PLPP3 | 3.02E-40 | -3.02 |
| TENM2 | 2.35E-15 | -3.03 |
| PRICKLE2-AS1 | 2.08E-21 | -3.03 |
| JAM2 | 9.28E-34 | -3.03 |
| LRP1 | 1.35E-27 | -3.03 |
| CCDC3 | 1.56E-14 | -3.03 |
| CNN1 | 8.59E-14 | -3.04 |
| LAMA2 | 2.06E-28 | -3.04 |
| ADGRF5 | 6.32E-24 | -3.04 |
| GASK1B | 1.33E-32 | -3.04 |
| KCNA1 | 9.31E-11 | -3.04 |
| EGR1 | 1.27E-16 | -3.05 |
| CT62 | 1.06E-08 | -3.05 |
| LIN7A | 2.86E-20 | -3.05 |
| LDB2 | 1.92E-25 | -3.05 |
| ABCA9 | 1.57E-16 | -3.06 |
| LINC00968 | 8.25E-08 | -3.06 |
| FHL5 | 1.07E-10 | -3.06 |
| CYBRD1 | 1.77E-29 | -3.06 |
| PALM | 5.63E-18 | -3.07 |
| KLF4 | 1.76E-17 | -3.07 |
| CD93 | 1.53E-22 | -3.07 |
| CRIM1 | 4.60E-40 | -3.07 |
| MYCT1 | 4.16E-24 | -3.07 |
| MATN2 | 2.51E-23 | -3.08 |
| CACNA2D1 | 2.24E-25 | -3.08 |
| APLNR | 1.00E-16 | -3.08 |
| C1QTNF9 | 1.32E-26 | -3.08 |
| ALDH1L1-AS1 | 2.77E-12 | -3.09 |
| ECRG4 | 3.06E-08 | -3.09 |
| ABCD2 | 2.26E-14 | -3.09 |
| BMPER | 2.08E-12 | -3.09 |
| SYNE3 | 3.23E-23 | -3.09 |
| WSCD1 | 2.48E-21 | -3.10 |
| COL25A1 | 2.23E-19 | -3.11 |
| RDH5 | 6.64E-22 | -3.11 |
| CDH5 | 2.54E-23 | -3.11 |
| PREX2 | 6.75E-21 | -3.11 |
| SLIT3-AS2 | 3.73E-24 | -3.11 |
| PDE11A | 1.35E-12 | -3.12 |
| ADGRL4 | 2.13E-24 | -3.12 |
| AKR1C3 | 4.88E-16 | -3.12 |
| CLDN11 | 1.17E-20 | -3.12 |
| IL6-AS1 | 2.02E-15 | -3.12 |
| COL17A1 | 5.01E-08 | -3.13 |
| LINC02587 | 4.07E-08 | -3.13 |
| FAM110D | 9.60E-29 | -3.13 |
| TAT-AS1 | 5.50E-09 | -3.13 |
| ECM2 | 2.54E-27 | -3.14 |
| IGFBP6 | 2.15E-18 | -3.15 |
| CD209 | 4.67E-16 | -3.16 |
| DUSP1 | 2.44E-27 | -3.16 |
| ABCA9-AS1 | 4.01E-17 | -3.16 |
| SAA2 | 1.86E-12 | -3.16 |
| SYNPO2 | 2.30E-15 | -3.16 |
| PECAM1 | 9.01E-27 | -3.16 |
| GIMAP6 | 2.19E-24 | -3.16 |
| PLAC9 | 1.00E-24 | -3.16 |
| NWD2 | 2.63E-11 | -3.16 |
| PDGFD | 1.97E-31 | -3.17 |
| PLAAT5 | 1.30E-09 | -3.17 |
| EHD2 | 4.58E-26 | -3.17 |
| ABCB5 | 1.27E-14 | -3.18 |
| ESR1 | 1.28E-13 | -3.18 |
| MAB21L1 | 1.50E-17 | -3.18 |
| MYOCD | 2.75E-11 | -3.18 |
| HCAR1 | 1.92E-20 | -3.19 |
| COL14A1 | 5.12E-21 | -3.19 |
| CD34 | 2.96E-23 | -3.19 |
| COL4A6 | 1.31E-14 | -3.20 |
| FMOD | 9.50E-17 | -3.20 |
| ADAMTS15 | 5.09E-16 | -3.21 |
| C1QTNF7 | 4.40E-14 | -3.21 |
| FAM162B | 2.85E-22 | -3.22 |
| TMTC1 | 4.14E-21 | -3.23 |
| CTSG | 1.35E-12 | -3.23 |
| ADRB1 | 1.83E-14 | -3.24 |
| HSD11B1 | 4.02E-19 | -3.25 |
| LINC02568 | 1.90E-09 | -3.25 |
| IL33 | 4.88E-16 | -3.25 |
| ENPP2 | 7.54E-18 | -3.26 |
| LMX1A | 1.80E-08 | -3.26 |
| ALDH1A2 | 6.05E-13 | -3.27 |
| AKR1C4 | 3.16E-20 | -3.27 |
| AK5 | 1.70E-23 | -3.27 |
| SBK3 | 1.30E-13 | -3.27 |
| CITED1 | 1.80E-10 | -3.27 |
| MYRIP | 1.81E-18 | -3.27 |
| ADGRD1 | 1.77E-19 | -3.27 |
| MGLL | 1.63E-20 | -3.27 |
| PIP | 1.99E-05 | -3.27 |
| MRGPRF | 7.72E-23 | -3.28 |
| MMD | 5.95E-26 | -3.28 |
| NGFR-AS1 | 2.70E-16 | -3.29 |
| TP63 | 5.35E-12 | -3.29 |
| ADAMTS5 | 2.04E-36 | -3.29 |
| C4BPA | 3.55E-09 | -3.29 |
| ANGPTL2 | 1.55E-24 | -3.30 |
| SELENOP | 7.58E-26 | -3.30 |
| FOS | 4.35E-22 | -3.30 |
| MAP1LC3C | 6.85E-20 | -3.30 |
| NEUROG2 | 8.90E-10 | -3.31 |
| AVPR1A | 1.21E-20 | -3.31 |
| NLGN4Y | 4.70E-23 | -3.31 |
| KY | 6.19E-26 | -3.32 |
| SAA2-SAA4 | 1.17E-13 | -3.32 |
| EBF2 | 1.22E-21 | -3.32 |
| MYH11 | 2.38E-24 | -3.33 |
| INSYN2B | 4.03E-13 | -3.33 |
| HEPACAM2 | 2.30E-07 | -3.33 |
| SELP | 2.38E-15 | -3.33 |
| APOLD1 | 1.12E-27 | -3.34 |
| GRIK3 | 1.20E-13 | -3.34 |
| LRP1-AS | 1.81E-23 | -3.34 |
| ITGA7 | 1.36E-21 | -3.34 |
| ERBB4 | 1.28E-14 | -3.35 |
| TGFBR3 | 1.85E-26 | -3.36 |
| TRDN | 1.53E-07 | -3.37 |
| ADCY5 | 2.18E-23 | -3.37 |
| KL | 2.59E-15 | -3.38 |
| ZBTB16 | 2.75E-09 | -3.38 |
| MMRN2 | 6.63E-33 | -3.38 |
| GPR146 | 9.49E-38 | -3.38 |
| PCOLCE2 | 5.59E-11 | -3.39 |
| CMA1 | 3.52E-16 | -3.40 |
| ADIRF | 1.36E-24 | -3.41 |
| BMX | 9.76E-21 | -3.42 |
| TSPAN7 | 4.17E-23 | -3.42 |
| SCN7A | 3.89E-12 | -3.42 |
| ASPA | 1.34E-20 | -3.42 |
| AVPR2 | 8.11E-18 | -3.43 |
| SLC4A4 | 2.27E-27 | -3.43 |
| AQP7B | 2.91E-14 | -3.43 |
| HSD11B1-AS1 | 2.81E-19 | -3.43 |
| DGAT2 | 2.77E-20 | -3.44 |
| BHMT2 | 1.80E-17 | -3.44 |
| PGM5P3-AS1 | 5.89E-12 | -3.45 |
| AQP1 | 9.07E-28 | -3.45 |
| MEOX1 | 3.03E-16 | -3.46 |
| LOC112267859 | 1.06E-13 | -3.46 |
| CLDN5 | 1.46E-18 | -3.47 |
| NAT8L | 1.94E-15 | -3.47 |
| CYP4B1 | 1.49E-11 | -3.47 |
| AQP7P1 | 1.37E-13 | -3.47 |
| TNXA | 2.82E-16 | -3.47 |
| SSTR1 | 4.39E-12 | -3.47 |
| COL6A6 | 1.07E-21 | -3.48 |
| EDNRB | 2.47E-23 | -3.48 |
| SLIT3 | 5.34E-21 | -3.48 |
| OGN | 1.57E-19 | -3.48 |
| DCN | 1.13E-22 | -3.49 |
| STEAP4 | 8.43E-20 | -3.49 |
| LINC01697 | 1.37E-15 | -3.50 |
| TEK | 2.24E-25 | -3.51 |
| CASQ2 | 1.23E-09 | -3.51 |
| TAT | 1.21E-09 | -3.51 |
| CCDC69 | 4.64E-20 | -3.51 |
| S1PR1 | 4.67E-27 | -3.52 |
| SAA1 | 4.00E-15 | -3.53 |
| LEPR | 3.32E-37 | -3.53 |
| GRIA1 | 2.11E-14 | -3.54 |
| EPAS1 | 3.60E-28 | -3.54 |
| RXRG | 2.51E-13 | -3.55 |
| NRIP3-DT | 7.37E-16 | -3.55 |
| MEOX2 | 7.75E-17 | -3.55 |
| PDE3B | 3.63E-16 | -3.55 |
| PTPRQ | 6.22E-11 | -3.56 |
| AKR1C1 | 4.70E-20 | -3.56 |
| SCN2B | 1.34E-27 | -3.56 |
| GYS2 | 9.74E-20 | -3.58 |
| PGM5P4-AS1 | 1.96E-19 | -3.59 |
| CHL1 | 3.18E-26 | -3.59 |
| RELN | 4.52E-18 | -3.59 |
| PI16 | 5.87E-12 | -3.61 |
| GABRA2 | 6.33E-20 | -3.61 |
| GRPR | 9.91E-13 | -3.62 |
| CCN5 | 5.65E-18 | -3.62 |
| LOC102723475 | 7.48E-19 | -3.62 |
| LIFR | 2.56E-29 | -3.62 |
| FGF10-AS1 | 5.10E-19 | -3.63 |
| CLIC5 | 7.25E-28 | -3.63 |
| PCDH19 | 4.70E-24 | -3.64 |
| ABCC9 | 6.09E-31 | -3.66 |
| PGR | 3.46E-15 | -3.66 |
| SOX17 | 3.14E-24 | -3.67 |
| ACACB | 1.12E-31 | -3.67 |
| ARHGAP20 | 8.00E-24 | -3.69 |
| MIR936 | 1.65E-12 | -3.69 |
| LRRN4CL | 6.52E-26 | -3.70 |
| GLDN | 1.44E-19 | -3.72 |
| PGM5 | 4.38E-28 | -3.72 |
| FGF10 | 1.87E-18 | -3.74 |
| FZD4 | 1.39E-35 | -3.74 |
| ANGPTL1 | 2.82E-23 | -3.75 |
| ADRB2 | 2.04E-23 | -3.75 |
| CCL15-CCL14 | 2.10E-19 | -3.75 |
| CCL14 | 2.88E-19 | -3.76 |
| CBLN1 | 8.96E-11 | -3.77 |
| TGFBR2 | 3.10E-50 | -3.78 |
| ABLIM3 | 1.60E-21 | -3.79 |
| PENK | 1.69E-11 | -3.80 |
| CLDN19 | 2.10E-18 | -3.81 |
| TNN | 6.70E-20 | -3.81 |
| SLC2A4 | 2.00E-22 | -3.81 |
| MYZAP | 7.24E-31 | -3.82 |
| TNS1 | 2.56E-29 | -3.83 |
| CPED1 | 2.74E-27 | -3.83 |
| AADAC | 1.91E-12 | -3.83 |
| CLEC3B | 1.06E-24 | -3.83 |
| LINC01537 | 4.53E-28 | -3.85 |
| KCNIP2-AS1 | 1.17E-32 | -3.86 |
| SCGB2A2 | 4.76E-07 | -3.87 |
| PGM5-AS1 | 2.03E-25 | -3.88 |
| CCDC85A | 3.52E-32 | -3.89 |
| LOC101929216 | 1.67E-12 | -3.89 |
| OXTR | 4.09E-26 | -3.90 |
| AKR1C2 | 1.14E-15 | -3.91 |
| BTNL9 | 2.01E-20 | -3.94 |
| NDNF | 2.86E-22 | -3.94 |
| MYMX | 1.24E-26 | -3.94 |
| GHR | 3.42E-29 | -3.94 |
| ADGRD2 | 3.96E-11 | -3.94 |
| PAMR1 | 4.53E-28 | -3.95 |
| ACKR1 | 6.03E-17 | -3.96 |
| ADRA2A | 1.61E-27 | -3.96 |
| PPARG | 3.33E-34 | -3.96 |
| SCGN | 5.30E-12 | -3.97 |
| AOX1 | 2.70E-25 | -3.97 |
| SEMA3G | 2.15E-25 | -3.98 |
| MME | 1.01E-34 | -3.99 |
| PDE2A | 2.88E-29 | -4.00 |
| PLXNA4 | 3.41E-19 | -4.01 |
| CDO1 | 1.49E-24 | -4.01 |
| NPR1 | 1.54E-38 | -4.01 |
| SLC7A3 | 1.27E-16 | -4.02 |
| CAV1 | 6.34E-41 | -4.05 |
| APOB | 1.03E-17 | -4.07 |
| SORBS1 | 3.81E-37 | -4.08 |
| SLC14A2 | 3.87E-25 | -4.09 |
| CPA1 | 3.48E-22 | -4.10 |
| TMEM37 | 9.53E-31 | -4.11 |
| LYVE1 | 8.71E-24 | -4.11 |
| G0S2 | 4.73E-27 | -4.12 |
| SCN4A | 1.14E-21 | -4.13 |
| GLP2R | 4.86E-27 | -4.13 |
| C2CD4B | 5.07E-22 | -4.13 |
| MMRN1 | 5.72E-19 | -4.15 |
| SCN4B | 2.52E-30 | -4.15 |
| FOSB | 5.95E-32 | -4.17 |
| LHCGR | 6.29E-17 | -4.17 |
| PDK4 | 7.52E-26 | -4.20 |
| PTGER3 | 5.94E-39 | -4.21 |
| ANGPT4 | 1.61E-21 | -4.23 |
| SMYD1 | 2.19E-15 | -4.24 |
| SPX | 3.46E-20 | -4.24 |
| CFD | 5.64E-20 | -4.26 |
| LPL | 1.23E-22 | -4.29 |
| ATOH8 | 1.43E-26 | -4.29 |
| KCNC2 | 7.80E-14 | -4.31 |
| SCGB1D2 | 6.51E-09 | -4.33 |
| KCNIP2 | 6.20E-36 | -4.33 |
| TNXB | 6.25E-24 | -4.35 |
| THBS4 | 2.14E-27 | -4.36 |
| VEGFD | 1.31E-27 | -4.36 |
| LINC01279 | 5.26E-30 | -4.38 |
| GPC3 | 4.79E-22 | -4.40 |
| RAB3C | 1.81E-34 | -4.40 |
| SVEP1 | 7.90E-30 | -4.43 |
| ALDH1L1 | 1.84E-20 | -4.44 |
| ADRA1A | 9.06E-26 | -4.44 |
| NNAT | 1.20E-31 | -4.45 |
| SGCG | 5.28E-17 | -4.46 |
| ATP1A2 | 1.45E-19 | -4.46 |
| VWF | 3.77E-40 | -4.46 |
| ACSM5 | 7.66E-31 | -4.48 |
| GALNT15 | 1.38E-41 | -4.49 |
| TNMD | 1.17E-19 | -4.50 |
| GPX3 | 6.08E-37 | -4.55 |
| HSPB6 | 1.98E-20 | -4.57 |
| GDF10 | 1.01E-15 | -4.57 |
| ACVR1C | 3.12E-34 | -4.57 |
| SLC19A3 | 8.47E-36 | -4.60 |
| MASP1 | 8.58E-23 | -4.62 |
| CLSTN2 | 6.69E-30 | -4.62 |
| CAVIN2 | 3.16E-27 | -4.62 |
| ABCA8 | 1.03E-25 | -4.63 |
| LINCADL | 1.77E-24 | -4.63 |
| GYG2 | 1.49E-32 | -4.63 |
| AOC3 | 4.56E-34 | -4.67 |
| LINC02884 | 1.62E-25 | -4.68 |
| CRHBP | 3.09E-35 | -4.71 |
| AGTR1 | 8.29E-36 | -4.72 |
| ADCYAP1R1 | 4.62E-44 | -4.79 |
| TRHDE | 1.37E-25 | -4.82 |
| AOC4P | 2.55E-37 | -4.88 |
| CES1P2 | 4.22E-22 | -4.92 |
| HBA1 | 2.23E-25 | -4.93 |
| HBA2 | 5.28E-26 | -4.97 |
| GPIHBP1 | 1.58E-31 | -5.00 |
| CSF3 | 3.72E-16 | -5.01 |
| THRSP | 2.65E-32 | -5.01 |
| LVRN | 1.05E-34 | -5.02 |
| ITIH5 | 3.05E-33 | -5.02 |
| GPAM | 4.63E-51 | -5.03 |
| KLB | 2.84E-44 | -5.04 |
| TMEM252 | 3.37E-23 | -5.05 |
| FRMD1 | 2.07E-28 | -5.06 |
| FHL1 | 3.55E-37 | -5.10 |
| HEPN1 | 9.27E-37 | -5.10 |
| CIDEA | 4.44E-21 | -5.13 |
| PIGR | 1.40E-13 | -5.13 |
| SCARA5 | 2.85E-26 | -5.17 |
| TMEM132C | 6.09E-19 | -5.18 |
| LGALS12 | 2.97E-23 | -5.22 |
| NPY2R-AS1 | 1.55E-18 | -5.22 |
| MAOA | 1.36E-39 | -5.22 |
| ADH1A | 4.85E-37 | -5.23 |
| TRHDE-AS1 | 1.19E-22 | -5.28 |
| AQP7 | 3.56E-23 | -5.33 |
| HSD17B13 | 3.29E-42 | -5.36 |
| HEPACAM | 5.08E-41 | -5.37 |
| DPT | 4.25E-41 | -5.39 |
| ADH1C | 3.65E-27 | -5.46 |
| HSPB7 | 2.37E-34 | -5.51 |
| BPIFB2 | 5.27E-17 | -5.52 |
| RBP4 | 4.37E-25 | -5.52 |
| CD36 | 5.71E-34 | -5.53 |
| CHRDL1 | 1.34E-38 | -5.60 |
| LIPE | 4.90E-47 | -5.63 |
| ADH1B | 3.04E-23 | -5.66 |
| CES1P1 | 2.28E-27 | -5.67 |
| FABP4 | 1.03E-25 | -5.71 |
| CES1 | 2.43E-35 | -5.92 |
| HBB | 2.75E-34 | -5.92 |
| LINC02996 | 3.26E-24 | -5.99 |
| TIMP4 | 4.57E-36 | -6.08 |
| PCK1 | 9.62E-25 | -6.10 |
| CIDEC | 2.31E-33 | -6.12 |
| C14orf180 | 1.07E-23 | -6.23 |
| CPB1 | 7.38E-20 | -6.24 |
| TRARG1 | 8.37E-25 | -6.28 |
| CD300LG | 4.22E-31 | -6.35 |
| GPD1 | 3.63E-41 | -6.55 |
| PLIN4 | 4.52E-43 | -6.72 |
| GLYAT | 4.71E-45 | -6.75 |
| ADIPOQ | 2.00E-30 | -6.75 |
| PLIN1 | 9.65E-40 | -6.78 |
| ADIPOQ-AS1 | 8.12E-32 | -7.15 |
| MYOC | 8.05E-36 | -7.51 |
| LEP | 5.81E-59 | -8.28 |

DEG, differentially expressed genes; TNBC, triple negative breast cancer; FC, fold change; FDR, false discovery rate.
